# Targeting CD155 in lung adenocarcinoma: A5 nanobody-based therapeutics for precision treatment and enhanced drug delivery

**DOI:** 10.1038/s41392-025-02301-z

**Published:** 2025-07-10

**Authors:** Kyunghee Noh, Soyeon Yi, Hyeran Kim, Jieun Lee, Suhyeon Kim, Wonbeak Yoo, Eunkyeong Jung, Jinsol Choi, Hwangseo Park, Seungha Hwang, Jin Young Kang, Kwang-Hyun Park, Heewon Park, Yong-kyu Lee, Eun-Kyung Lim, Taejoon Kang, Juyeon Jung

**Affiliations:** 1 Bionanotechnology Research Center, Korea Research Institute of Bioscience and Biotechnology (KRIBB), 125 Gwahak-ro, Yuseong-gu, Daejeon, 34141 Republic of Korea; 2 Department of Nanobiotechnology, KRIBB School of Biotechnology, UST, 217 Gajeong-ro, Yuseong-gu, Daejeon, 34113 Republic of Korea; 3 College of Pharmacy, Kyung Hee University, Seoul, 02447 Republic of Korea; 4 Personalized Genomic Medicine Research Center, Korea Research Institute of Bioscience and Biotechnology (KRIBB), 125 Gwahak-ro, Yuseong-gu, Daejeon, 34141 Republic of Korea; 5 College of Pharmacy, Korea University, Sejong, 30019 Republic of Korea; 6 Deparment of Bioscience and Biotechnology, Sejong University, 209 Neungdong-ro, Kwangjin-gu, Seoul, 05006 Republic of Korea; 7 Department of Chemistry, Korea Advanced Institute of Science and Technology (KAIST), Daejeon, Republic of Korea; 8 Critical Diseases Diagnostics Convergence Research Center, Korea Research Institute of Bioscience and Biotechnology (KRIBB), Daejeon, 34141 Republic of Korea; 9 Department of Chemical and Biomolecular Engineering, Korea Advanced Institute of Science and Technology (KAIST), Daejeon, 34141 Republic of Korea; 10 Department of Chemical and Biological Engineering, Korea National University of Transportation, Chungju, 27469 Republic of Korea; 11 School of Pharmacy, Sungkyunkwan University, 2066 Seobu-ro, Jangan-gu, Suwon, 16419 Republic of Korea

**Keywords:** Drug development, Molecular medicine

## Abstract

This study presents a novel approach targeting CD155, an overexpressed protein in lung adenocarcinoma (LUAD), using nanobodies with exceptional precision and efficacy. The significant upregulation of CD155 in LUAD, associated with poor patient outcomes, highlights its potential as a therapeutic target. An anti-CD155 nanobody (A5 Nb) is developed that binds to CD155-positive lung cancer cells with high affinity (A5 Nb *K*_d_ = 0.23 nM). The complementarity-determining region of A5 Nb forms hydrophobic interactions and hydrogen bonds with CD155, promoting selective binding and stabilization of A5 Nb-CD155 complex. This interaction inhibits focal adhesion signaling by downregulating paxillin (PXN), leading to a >50% reduction in cell migration. Additionally, A5 Nb conjugated to liposomes loaded with doxorubicin (A5-LNP-DOX) demonstrates a 2- to 3-fold increase in uptake and cytotoxicity in CD155-positive A549 cells, suggesting its potential as a targeted drug delivery system. Therapeutic efficacy was further validated in both lung orthotopic mouse models and lung cancer organoid xenografts, where A5-LNP-DOX exhibited robust antitumor effects and selective targeting. The CD155-PXN axis emerges as a clinically relevant target, correlating with poor outcomes in patients with lung cancer. This study highlights the therapeutic potential of A5 nanobodies in targeting CD155-overexpressing lung cancer cells and offers insights for future developments in lung cancer therapeutics.

## Introduction

Lung cancer is the most frequently diagnosed type of cancer and is the leading cause of cancer-related deaths globally in both men and women.^1^ Among the various histological subtypes, non-small cell lung cancer (NSCLC) accounts for ~85% of all cases, with small cell lung cancer (SCLC) comprising the remainder.^2,3^ Targeted therapies have transformed the treatment landscape of NSCLC, particularly through inhibitors of oncogenic drivers such as epidermal growth factor receptor (EGFR) and anaplastic lymphoma kinase (ALK).^4–6^ However, clinical limitations such as the development of drug resistance, limited applicability across patient populations, and adverse effects continue to hinder long-term outcomes.^7,8^ Moreover, the poor tumor penetration of many biologics and the systemic toxicity of small-molecule chemotherapies further complicate effective disease management. These issues highlight the urgent need for novel therapeutic strategies that combine high specificity with improved delivery efficiency. Such approaches must also account for tumor heterogeneity and drug resistance mechanisms, which remain major challenges in achieving durable responses. Additionally, many patients do not benefit from existing targeted therapies due to a lack of actionable mutations or molecular heterogeneity within the tumor, leading to suboptimal treatment outcomes. An effective approach must therefore not only target cancer-specific biomarkers but also ensure adequate drug accumulation in tumor tissue while minimizing off-target effects.^9,10^

CD155, also known as the poliovirus receptor (PVR), has recently emerged as a promising target for immunotherapy due to its role in enhancing anti-tumor responses.^11^ Its overexpression in several malignancies, including lung adenocarcinoma (LUAD), is associated with poor prognosis.^12,13^ CD155 interacts with co-stimulatory (CD226) and co-inhibitory (TIGIT, CD96) receptors on T cells and NK cells, modulates immune responses and frequently resulting in a suppressed antitumor immunity in the tumor microenvironment.^14^ Beyond its immune functions, CD155 is also implicated in tumor cell adhesion, migration, and proliferation through intracellular pathways involving focal adhesion complexes, making it a compelling dual-purpose target for both immunotherapeutic modulation and selective drug delivery.^15,16^ However, most existing research has focused primarily on CD155 mediated immunoregulatory functions, and its potential as a therapeutic delivery target and the modulation of focal adhesion signaling pathways, which play crucial roles in cancer progression remains largely unexplored. Recent studies have begun to elucidate CD155’s involvement in epithelial-mesenchymal transition (EMT) and cytoskeletal reorganization, further supporting its therapeutic relevance.^17,18^ Furthermore, targeting CD155 may offer a strategy to impair both immune evasion and the intrinsic invasiveness of tumor cells, making it a uniquely advantageous molecular entry point. By capitalizing on these biological features, CD155-based delivery systems may also overcome limitations faced by traditional antibody-drug conjugates and checkpoint inhibitors.^19,20^ Addressing this unmet need may expand the utility of CD155 beyond immune checkpoint blockade and enable a new class of targeted therapies.

Nanobodies (Nbs), the single-domain antigen-binding fragments derived from camelid heavy-chain antibodies, offer unique advantages over conventional antibodies in drug delivery.^21^ With a molecular size approximately one-tenth that of conventional monoclonal antibodies, Nbs demonstrate excellent solubility, thermal stability, deep tissue penetration, and suitability for large-scale production.^22^ Despite containing only three complementarity-determining regions (CDRs), the VHH domain could achieve high binding affinity in the nanomolar to picomolar range.^23^ These unique properties make Nbs particularly attractive for tumor-targeted drug delivery, enabling their efficient integration into nanoparticle and liposomal platforms. Nanobody technology is rapidly advancing due to its unique structural and functional advantages, these properties have opened new avenues in both therapeutic and diagnostic applications. The clinical potential and safety of nanobody-based therapeutics have already been validated by the approval of Caplacizumab, the first nanobody drug authorized by both the European Medicines Agency (EMA) and the U.S. Food and Drug Administration (FDA).^24^ This approval underscores the translational viability of nanobody platforms and supports their expansion into oncology. In addition, ongoing preclinical and clinical studies in oncology continue to demonstrate the versatility of nanobody formats in both imaging and drug delivery applications, further supporting their integration into advanced therapeutic systems. Importantly, nanobodies can be produced in microbial systems at reduced cost and with higher scalability than full-length antibodies, making them accessible tools for global cancer care. Their compatibility with modular design also facilitates multifunctional payload delivery, enabling simultaneous targeting, imaging, and therapy.^25,26^

In this study, we aimed to develop and evaluate anti-CD155 nanobody-conjugated liposomes as a targeted drug delivery system for LUAD. We hypothesized that these conjugates would enhance the delivery of anticancer drugs to CD155-expressing tumors, thereby improving therapeutic outcomes. Our findings demonstrate that A5-LNP-DOX significantly improves tumor targeting and antitumor efficacy in physiologically relevant LUAD models, including orthotopic and patient-derived organoid xenografts. These results highlight its potential as a nanobody-based strategy for targeted lung cancer therapy. Taken together, this strategy provides a versatile platform that may be adapted to enhance the efficacy of other chemotherapeutics, offering a broadly applicable framework for nanobody-guided drug delivery in oncology.

## Results

We initially analyzed CD155 expression across various cancer types using flow cytometry with two different antibodies (Supplementary Fig. 1a). We mined data from 21 TCGA cancer types using OncoLnc (http://www.oncolnc.org) to examine the log-rank *p*-value in Cox regression analysis, comparing CD155 expression across tumors. Cox regression analysis was performed using TCGA datasets. Our analysis revealed that CD155 expression in LUAD ranked first among the 21 cancer types based on survival *p*-values and FDR correction (Supplementary Table 1). High CD155 expression in LUAD patients was associated with poor outcomes and significantly shorter overall survival (*p* = 0.004) (Supplementary Fig. 1b), and was also consistently elevated in LUAD compared to normal lung tissue across public microarray datasets GSE116959, GSE19188, and GSE32863 (Supplementary Fig. 1c). We further confirmed this trend by evaluating CD155 protein levels in normal lung and LUAD tissues through immunohistochemistry, revealing higher expression in LUAD tissues (*p* = 0.0001) (Supplementary Fig. 1d).

### Generation and characterization of nanobodies binding to CD155 and functional effect of nanobodies

**Fig. 1:**
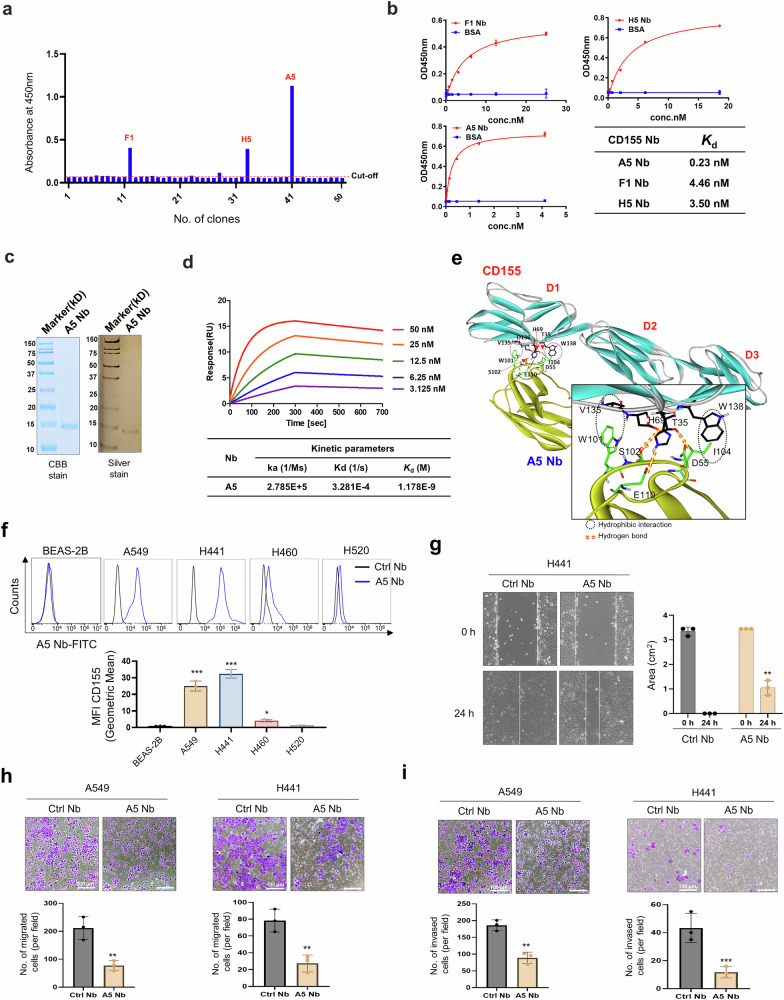
Screening and characterization of CD155 specific nanobodies. **a** Phage-displayed nanobody library is screened against immobilized CD155 antigen to identify high-affinity binders through iterative biopanning cycles. Identification of anti-CD155 nanobodies *via* ELISA. Specificity of binding for 50 phage clones screened against CD155. Three clones with higher absorbance values than the control was selected as potential candidates. Cut-off value = 0.056. **b** ELISA was used to compare the reactive sensitivity to CD155 with different gradient concentrations of anti-CD155 Nbs (F1, H5, and A5). The table displays the binding affinities (*K*_d_). **c** The purified recombinant proteins of A5 Nb separated by SDS-PAGE and stained with Coomassie Blue (CBB) and Siver stain. M: Marker. The molecular weight is indicated on the left. **d** A5 Nb binding to CD155 measured by SPR. Two-fold serial dilutions of CD155 from 50 nM to 3.125 nM injected onto the captured A5 Nb. Kinetic data from one representative experiment were fit to a 1:1 binding model. Summary of SPR affinity measurements. The equilibrium dissociation constant (Kd) is presented. Each concentration in the image above represents the mean value of three separate experiments. **e** Most probable binding pose of A5 Nb (yellow) with respect to CD155 (green). Intermolecular hydrogen bonds are represented by red dotted lines, while hydrophobic interaction contacts are denoted by black dotted circles. **f** The expression level of CD155 was measured by flow cytometry in BEAS-2B, A549, H441, H460, and H520 cell lines treated with CD155 A5 Nb. Isotype was used Ctrl Nb. **g** Representative images of scar and recovering of wounded areas (marked by white lines) on confluence monolayers of H441 cells at 0 and 24 h with CD155 A5 Nb (10 μg/mL) or Ctrl Nb (10 μg/mL) treatment and evaluation of scar wound healing in %. **h** Transwell migration assay. A549 and H441 cells were seeded on transwell insert and incubated with A5 Nb (10 μg/mL) or Ctrl Nb (10 μg/mL) for 24 h. **i** For the invasion assay, the membrane was pre-coated with 50 μL Matrigel. A549 and H441 cells were seeded on matrigel and incubated with A5 Nb (10 μg/mL) or Ctrl Nb (10 μg/mL) for 36 h. Scale bar = 100 μm for (**h**) and (**i**). Data represented as mean values ± SD, determined by two-tailed Student’s *t*-test *: *vs*. Ctrl Nb, *p < 0.05, **p < 0.01, and ***p < 0.001

A nanobody (Nb) library containing ~10^11^ unique variants with diversified CDR regions was screened to identify Nbs that specifically bind to CD155, resulting in the selection of 50 clones with strong binding affinity. To obtain clones with distinct base sequences, the sequences of the 50 clones were analyzed, and three clones (F1, H5, and A5) were selected for secondary screening (Fig. 1a). The binding affinity of these three clones to CD155 was evaluated, revealing nanomolar-range affinities (Fig. 1b). Among them, A5 Nb was selected based on its optimal kinetic parameters. The A5 Nb expressed in this study was composed of ~125 amino acids, corresponding to a theoretical molecular weight of ~15 kDa. Its purity was verified by SDS-PAGE analysis, followed by visualization using Coomassie Brilliant Blue and silver staining methods. (Fig. 1c). Surface plasmon resonance (SPR) was employed to determine the binding affinity of CD155 to the purified A5 Nb. After immobilization A5 Nb on a Biacore CM5 chip, various concentrations of purified CD155 were injected over the sensor surface. SPR analysis demonstrated a dissociation constant (*K*_d_) of 1.17 nM for the interaction between the A5 Nb and CD155 (Fig. 1d).

We investigated the structural features associated with the biochemical potency of the A5 nanobody (Nb) against CD155. As shown in Fig. 1e, molecular docking simulations predicted the most probable binding conformation of the A5 Nb to CD155. Notably, the Ser102 and Glu110 within the CDR of A5 Nb were predicted to form hydrogen bonds with Asp136 and His69 of CD155, respectively, suggesting specific molecular recognition. Structural modeling of the A5 Nb–CD155 complex suggested the presence of an additional hydrogen bond formed between the Asp55 residue of A5 Nb and Thr35 of CD155, indicating a potential contribution to binding stability. The interaction was further stabilized by hydrophobic contacts, particularly between Trp101 and Ile104 in the CDR of the A5 Nb and the side chains of Val135 and Trp138 in CD155. These hydrophobic interactions likely contribute significantly to the overall stability of the complex, especially due to their proximity to three hydrogen bonds formed between the A5 Nb and CD155. The strong nanomolar affinity of A5 Nb for CD155 is corroborated by structural evidence showing multiple stabilizing hydrogen bonds and hydrophobic constacts at the interface.

A5 Nb-targeting of CD155 was evaluated by flow cytometry for binding to lung cancer cell lines that expressed high CD155 levels. Efficient Nb binding was observed in the A549 and H441 cell lines, with binding levels 20–30 times higher than those in the normal human bronchial epithelial cell line BEAS-2B (Fig. 1f). We examined the effects of A5 Nb on in vitro wound healing, migration, and invasion. A5 Nb significantly affected wound healing (Fig. 1g), migration (Fig. 1h), and invasion (Fig. 1i) in the A549 and H441 cell lines, which expresses high levels of CD155. A5 Nb treated A549 and H441 cells showed over a 50% reduction in cell migration and invasive capacity, completely blocking CD155-mediated functional effects. Therefore, we suggest that A5 Nb is the best candidate due to its strong binding ability to lung cancer cell lines and its ability to inhibit cell migration and invasion. However, A5 Nb had no effect on the in vitro wound healing (Supplementary Fig. 2a), migration (Supplementary Fig. 2b) or invasion (Supplementary Fig. 2c) of the CD155 low-expressing H460 and H520 lung cancer cell lines. These results suggest that A5 Nb specifically targets and inhibits the migration of CD155-overexpressing cancer cells.

Taken together, A5 Nb specifically binds to CD155 and selectively inhibits migration and invasion in CD155-overexpressing lung cancer cells. These results highlight its potential as a targeted therapeutic agent for CD155-positive tumors.

### Effect of the A5 Nb on focal adhesion-related protein regulation

**Fig. 2:**
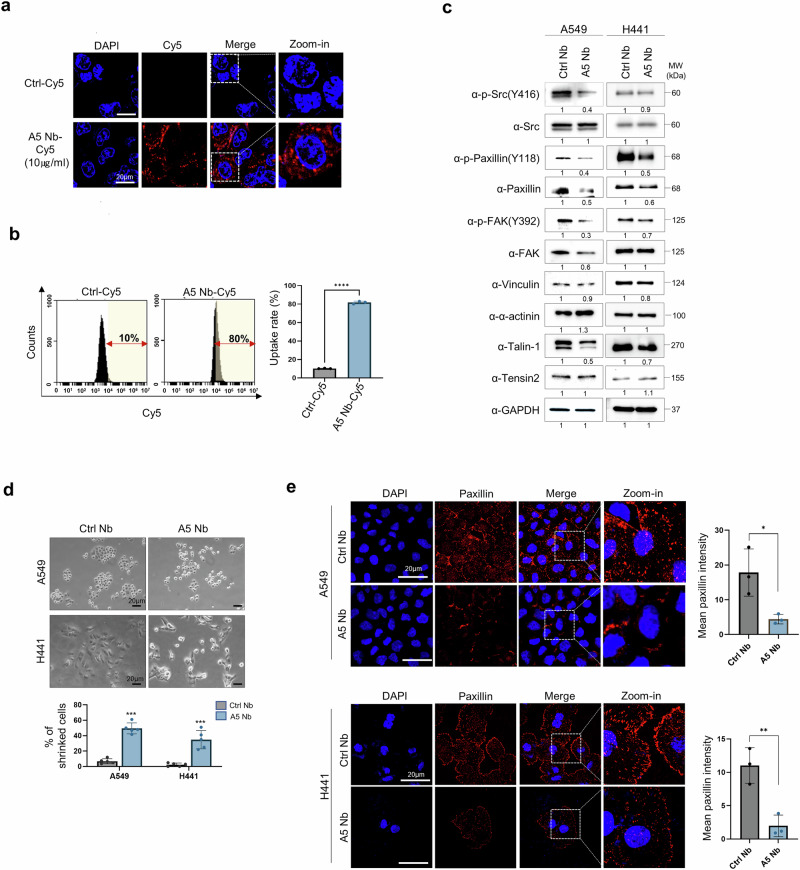
Internalization of CD155-targeted nanobody in CD155 overexpressed cancer cells and regulation of paxillin expression. **a** Cellular internalization images of fluorescently labeled nanobodies obtained by confocal laser scanning microscopy. A5 Nb conjugated with Cy5 and treated in A549 cells; scale bar indicated 20 μm. **b** % of cellular uptake was analyzed using flow cytometry. **c** pSrc/Src, pPaxillin/Paxillin, and pFAK/FAK pathway and focal adhesion complex proteins (Vinculin, α-actinin, Talin-1, and Tensin2) was measured in A549 and H441 cells after A5 Nb treatment *versus* Ctrl Nb. Quantified intensities are shown below each Western blot image. **d** Cellular morphology of A549 and H441 after A5 Nb treatment observed under an inverted light microscope. Quantification of shrinked cells was performed using ImageJ. Data represent mean ± SD of three independent experiments. **e** Representative images show expression of paxillin after treatment of A5 Nb in A549 and H441 cells compared to Ctrl Nb for 24 h. Nuclei were visualized with DAPI staining. Scale bar = 20 μm, A bar graph was used to present the average paxillin fluorescence intensity (*n* = 3). Data represented as mean values ± SD, determined by two-tailed Student’s *t*-test *: *vs*. Ctrl Nb, *p < 0.05, **p < 0.01, and ***p < 0.001

To evaluate the CD155 selectivity of A5 Nb, we investigated the cellular uptake and internalization of A5 Nb. Both Ctrl and A5 Nbs were labeled with Cy5 fluorescent dye and added to A549 cells for 24 h, followed by fluorescence imaging and flow cytometric analysis. A significant accumulation of fluorescence was observed in the membrane and cytoplasm, indicating that A5 Nb could be internalized and transported into the cytoplasm (Fig. 2a). Flow cytometry was performed to quantitatively assess the uptake of A5 Nb by A549 cells. As shown in Fig. 2b, an uptake rate of over 80% was observed in Cy5-labeled A5 Nb (A5 Nb-Cy5) incubated A549 cells. A conventional CD155 antibody was used as a comparator to evaluate the functional effects of the nanobody (Supplementary Fig. 3a, b). Similar to the nanobody, the conventional antibody was internalized into the cytoplasm (Supplementary Fig. 3a), showing an uptake rate of ~25% (Supplementary Fig. 3b). We then investigated whether the internalization and targeting of A5 Nb could affect cell migration properties, as previously demonstrated. The role of CD155-mediated epithelial-mesenchymal transition (EMT) in lung cancer cells was confirmed using qRT-PCR analysis to assess the differences in the expression levels of genes related to cell migration and EMT in Ctrl Nb- and A5 Nb-treated cells. However, no significant changes in the expression levels of these genes were observed in A5 Nb- or CD155 Ab-treated cells compared to those treated with control (Supplementary Fig. 4a, b).

CD155 has been implicated as a key regulator of cancer cell motility. During the migratory process, it is directed to the front edge of the moving cell, aligning with actin cytoskeleton components and integrin-αv.^12^ This spatial organization contributes to cell scattering through the activation of Src and FAK signaling cascades.^27^ We also examined changes in focal adhesion kinase signaling in CD155-high expressing A549 and H441 cells treated with A5 Nb. We observed downregulation of both phospho-PXN and total PXN in these cells (Fig. 2c). We focused on reducing total PXN expression, as its reduction was associated with a corresponding downregulation of phosphorylated PXN. The focal adhesion complex, consisting of multi-proteins such as vinculin, α-actinin, PXN, talin, and tensin, connects the actin cytoskeleton to the extracellular matrix (ECM) *via* integrin receptors activation.^28,29^ Talin-1 expression was decreased in A5 Nb-treated cells compared to Ctrl Nb treatment (Fig. 2c).

PXN plays a critical role in cell spreading, cytoskeletal organization, and adhesion.^30^ Downregulation of PXN by A5 Nb treatment led to a high incidence of cell shrinkage, which was typically associated with a lack of focal adhesion complexes (Fig. 2d). Moreover, A5 Nb-treated A549 and H441 cells exhibited a 4- to 5-fold reduction in PXN localization at nascent membrane adhesions (Fig. 2e). These results were consistently observed with the CD155 antibody (Supplementary Fig. 3c–e). To investigate whether PXN is functionally correlated with CD155 overexpression in lung cancer, we examined the effects of PXN on cell phenotypes. SiRNA-mediated silencing of PXN significantly reduced A549 cell migration and invasion (Supplementary Fig. 5a–c) and led to cell shrinkage (Supplementary Fig. 5d). These findings suggest that the expression of the FA protein, PXN, is significantly altered by blocking CD155 signaling, inducing cell edge shrinkage, and directly inhibiting cell migration.

Several studies have reported that CD155 activates the AKT/mTOR and MEK/ERK pathways and regulates tumor progression.^31,32^ However, we observed no effect on phospho-AKT and phospho-ERK levels in A5 Nb-treated A549 and H441 cells compared to Ctrl Nb treatment (Supplementary Fig. 6a). In addition, A5 Nb did not significantly alter Src/PXN/FAK activation or expression in CD155 low-expressing H460 and H520 lung cancer cell lines (Supplementary Fig. 6b). Interestingly, our findings with A5 Nb were consistently recapitulated when cells were treated with a 10-fold higher concentration of a conventional CD155 antibody. These included intracellular uptake, reduced PXN localization at the plasma membrane, cell shrinkage phenotype (Supplementary Fig. 3c–e), and unchanged EMT marker (Supplementary Fig. 4b) and AKT/ERK pathway (Supplementary Fig. 6c) in CD155-high cell lines. In contrast, no changes the focal adhesion-related proteins were observed in CD155-low cell lines (Supplementary Fig. 6d). Taken together, A5 Nb selectively disrupts focal adhesion dynamics by downregulating PXN in CD155-overexpressing lung cancer cells, leading to impaired cell adhesion and migration.

### CD155/PXN overexpression is associated with tumor progression and poorer overall survival in patients with lung cancer

**Fig. 3:**
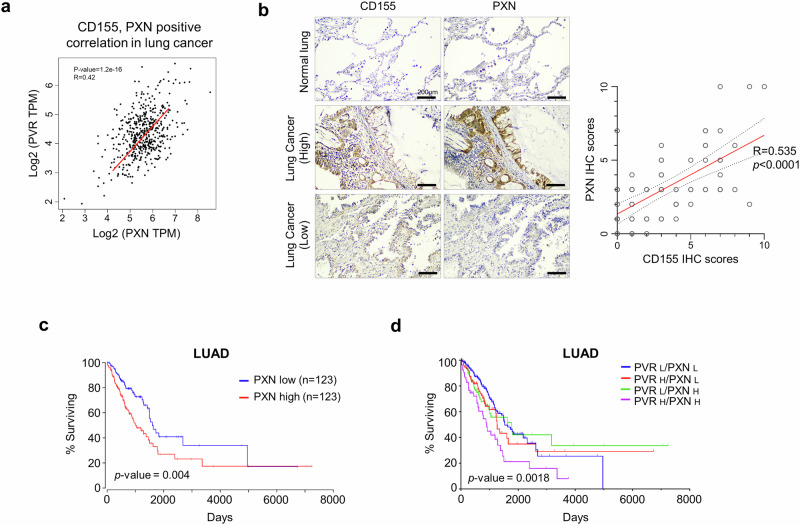
CD155 and PXN were upregulated in LUAD and predicts poor prognosis. **a** Pearson’s correlation analysis of the CD155 (PVR)/PXN genes was conducted using the GEPIA tool. PVR and PXN were positively correlated (*p* = 1.2e − 16; *R* = 0.42). **b** CD155 and PXN expression in normal lung tissues and lung cancer tissues. The TMA slide was immuno-stained with anti-CD155, and anti-paxillin antibodies. The plot depiction of IHC staining scores with same matched patients. Scale bar = 200 μm. **c** Survival outcomes based on Kaplan-Meier survival analysis of patients from The Cancer Genome Atlas (TCGA) according to PXN expression levels (*p* = 0.004). **d** Kaplan-Meier curve of overall survival in patients with lung cancer stratified according to CD155 and PXN mRNA expression levels (PVR^low^/PXN^low^ *vs*. PVR^high^/PXN^high^; *p* = 0.0018)

To validate the correlation between PXN and CD155, we used the GEPIA database to analyze the relationship between the PXN and CD155 genes. As shown in Fig. 3a, PXN and CD155 levels were positively correlated (*p* < 1.2e-16, *R* = 0.42).

To determine the potential clinical relevance of CD155 and PXN overexpression, we performed IHC staining for CD155 and PXN in a tissue microarray of tumor samples from patients with lung cancer. CD155 and PXN were significantly more highly expressed in tumor samples compared to normal samples, as shown in Fig. 3b. Moreover, in the same lung cancer samples from the tissue microarray, CD155 and PXN consistently exhibited similar expression patterns that both proteins were highly expressed in the high-expression group and markedly reduced in the low-expression group (Fig. 3b, middle and bottom). Importantly, the level of CD155 was strong positively correlated with that of PXN (*p* < 0.0001, *R* = 0.554) in lung cancer. The patients with lung adenocarcinoma in the TCGA database who had high PXN expression showed poorer outcomes and shorter overall survival (*p* = 0.004) (Fig. 3c). Notably, patients with both high CD155 and high PXN levels displayed significantly worse overall survival than patients with low levels of both markers (Fig. 3d, *p* = 0.0018). Overall, we conclude that the CD155-PXN axis is conserved in patients with lung cancer and serves as a potent therapeutic target.

### Characterization of LNP-DOX and A5-LNP-DOX

**Fig. 4:**
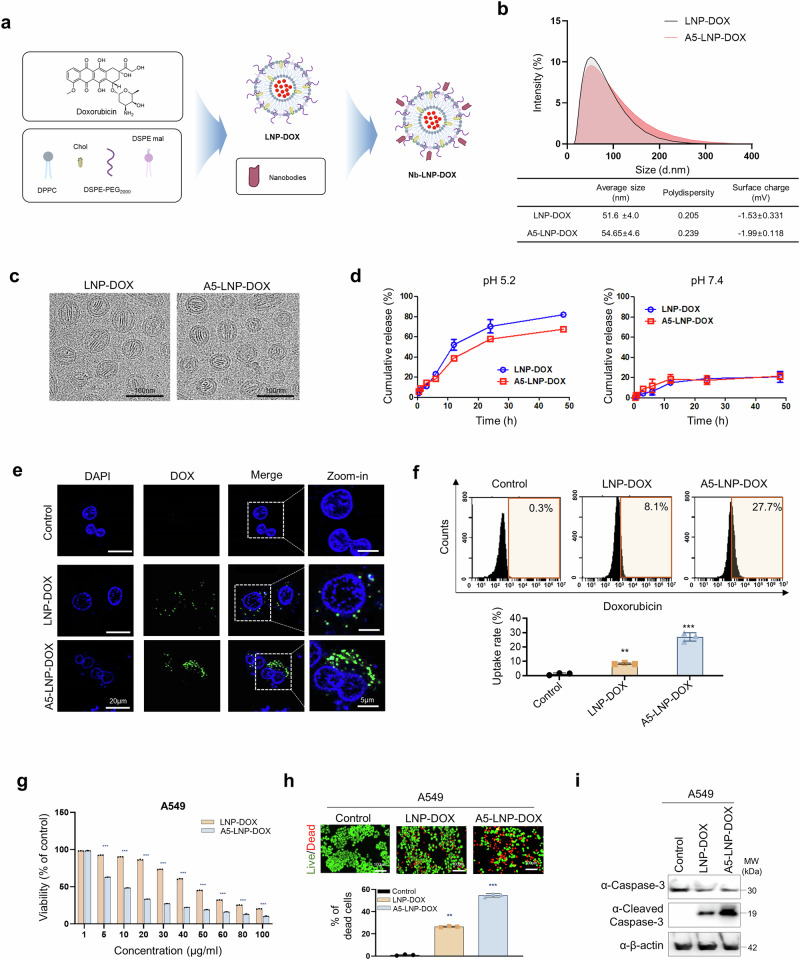
Liposome Characterization. **a** Flowchart of the synthesis of A5-LNP-DOX. **b** Particle size analysis of liposome by dynamic light scattering analysis. (Created by BioRender) **c** TEM images of liposome. Scale bar indicated 100 nm. **d** In vitro drug release profiles of doxorubicin from liposome. **e** Confocal imaging was performed to analyze the intracellular regions of liposomes. A549 cells were exposed to LNP-DOX and A5-LNP-DOX (DOX:green) for 4 h. The scale bars indicated 20 μm and 5 μm (zoom-in). **f** Cellular uptakes were evaluated by flow cytometry. All LNP-DOX concentration is 200 μg/mL. Data represented as mean values ± SD, determined by two-tailed Student’s *t*-test. *: *vs*. Control, **p < 0.01, and ***p < 0.001. **g** In vitro cytotoxicity was assessed on A549 cells for LNP-DOX or A5-LNP-DOX. Cells were treated with LNPs at various concentrations for 8 h, after which the medium was replaced and cells were further incubated for 24 h. The LNPs oncentration varied at 1, 5, 10, 20, 30, 40, 50, 60, 80, and 100 μg/mL. Data represent mean ± SD. (*n* = 3) *: *vs*. LNP-DOX, ***p < 0.001. **h** A549 cells were treated with LNP-DOX and A5-LNP-DOX for 8 h and then the medium was replaced and cells were cultured for an additional 24 h followed by live/dead staining. The cells were stained with calcein and propidium iodide resulting in the appearance of live cells in green and dead cells in red. Scale bar = 100 μm. Data represented as mean values ± SD, determined by two-tailed Student’s *t*-test. *: *vs*. Control, **p < 0.01, and ***p < 0.001. **i** Detection cleaved caspase-3 in apoptotic cells by western blots

Doxorubicin (DOX)-loaded liposomes capable of tumor penetration, referred to as LNP-DOX, were generated through DPPC lipid modification. The liposomes were obtained by mixing DPPC, followed by loading DOX using active drug loading, and then conjugating them with thiolated A5 Nb, resulting in A5-LNP-DOX (Fig. 4a). This process achieved a DOX encapsulation efficiency exceeding 65% and an encapsulation capacity >10%. The physicochemical properties of the particles were characterized using a size analyzer and transmission electron microscopy (TEM), which provided data on particle size, polydispersity index, surface charge, and morphology (Fig. 4b, c). The results indicated that the particle size of A5-LNP-DOX (54.65 ± 4.6 nm) was comparable to that of LNP-DOX (51.6 ± 4.0 nm), with both formulations exhibiting a spherical shape. These findings suggest that the incorporation of the A5 Nb coating did not significantly alter the physicochemical properties of the liposomes. Additionally, the negative zeta potential of LNP-DOX (-1.53 ± 0.331 mV) and A5-LNP-DOX (-1.99 ± 0.118 mV) supports the suitability of these formulations for drug delivery applications.

In vitro drug release under sink conditions was studied using a dialysis method. The release profiles of DOX from LNP-DOX and A5-LNP-DOX were assessed under neutral (pH 7.4) and acidic (pH 5.2) conditions. After 48 h of incubation, DOX release was 81% from LNP-DOX and 67% from A5-LNP-DOX under acidic conditions, representing ~4- and 3.3-fold increases, respectively, compared to the release at pH 7.4 (Fig. 4d). This enhanced release under acidic conditions is attributed to the protonation of phospholipid head groups, which leads to destabilization of the bilayer structure and increased permeability, thereby facilitating the release of encapsulated drugs.^33,34^

To achieve efficient induction of apoptosis in cancer cells, the anticancer drug DOX must be effectively targeted for intracellular delivery. The intracellular localization of LNP-DOX and A5-LNP-DOX in A549 and H441 cells was investigated using confocal microscopy. After 4 h of incubation, DOX fluorescence was predominantly observed in the cytosol of A5-LNP-DOX treated cells. The intensity of DOX-associated green fluorescence was markedly higher in the A5-LNP-DOX group compared to the LNP-DOX group, indicating effective active targeting *via* A5 Nb (Fig. 4e and Supplementary Fig. 7a). Quantitative analysis of cellular uptake in A549 cells revealed that ~27.7% of cells internalized A5-LNP-DOX, whereas only 8.1% internalized LNP-DOX (Fig. 4f). Similar results were in H441 cells (Supplementary Fig. 7b). However, in CD155-negative BEAS-2B cells, intracellular localization and uptake were negligible for both formulations (Supplementary Fig. 7a, b). These results confirm that A5-LNP-DOX selectively targets CD155-overexpressing cells and is internalized *via* CD155-mediated endocytosis, leading to effective cytoplasmic delivery of DOX and supporting its mechanistic basis for antitumor efficacy.

Furthermore, we evaluated the cytotoxicity of LNP-DOX and A5-LNP-DOX on A549 and H441 cells. Both LNP-DOX and A5-LNP-DOX exhibited dose-dependent cytotoxic effects, with A5-LNP-DOX displaying enhanced potency compared to LNP-DOX (Fig. 4g and Supplementary Fig. 7c). Cell viability was further assessed using live/dead staining with LNP-DOX or A5-LNP-DOX (10 μg/mL). After treatment, a higher count of red-stained dead cells was detected in A549 and H441 cells treated with A5-LNP-DOX compared to those treated with LNP-DOX (Fig. 4h and Supplementary Fig. 7d). Moreover, active caspase-3 were significantly higher in A5-LNP-DOX-treated cells (Fig. 4i and Supplementary Fig. 7e), indicating increased apoptosis. Overall, A5-LNP-DOX treated cells demonstrated higher dead cell counts and apoptosis markers than LNP-DOX treated cells. However, BEAS-2B cells showed no significant changes in cytotoxicity, live/dead staining, and active caspase-3 (Supplementary Fig. 7c–e). Taken together, A5-LNP-DOX demonstrated superior intracellular delivery and cytotoxicity in CD155-overexpressing lung cancer cells compared to non-targeted LNP-DOX. These results highlight the potential of A5 Nb-conjugated liposomes as an effective and selective drug delivery system for targeted cancer therapy.

### CD155-targeting nanobody-liposomes enhance tumor accumulation

To evaluate tumor-targeting efficiency, A549 tumor-bearing mice were intravenously injected with LNP-ICG or A5-LNP-ICG (8 mg/kg), and whole-body fluorescence imaging was performed at 24 h and 48 h post-injection (Supplementary Fig. 8). A5-LNP-ICG exhibited stronger and more sustained fluorescence signals at tumor sites compared to LNP-ICG.

Ex vivo analysis at 48 h confirmed this tumor-selective accumulation, showing significantly higher fluorescence intensity in tumors treated with A5-LNP-ICG. Notably, no detectable fluorescence signal was observed in major organs such as the liver, heart, lungs, spleen, and kidneys. Quantitative analysis revealed a 3.6-fold increase in tumor accumulation relative to LNP-ICG (*p* < 0.05), supporting the CD155-targeted delivery capability of A5-LNP.

### Therapeutic efficacy of CD155-targeted nanobody-liposomes in an orthotopic lung cancer model

**Fig. 5:**
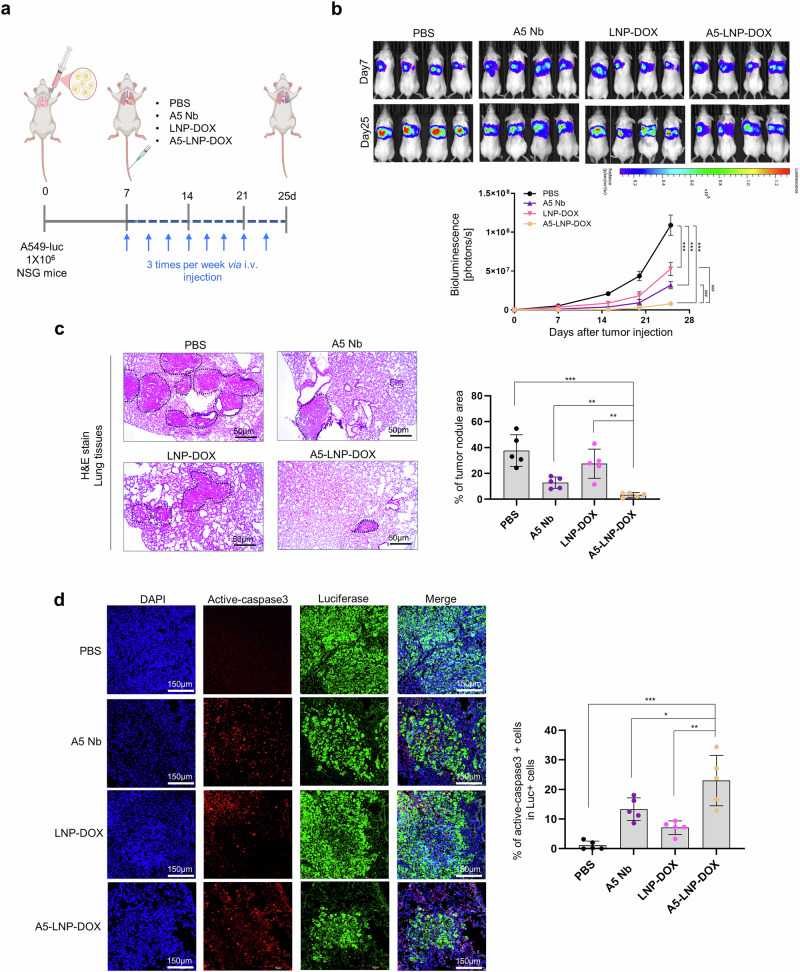
Development of the orthotopic A549-luc tumor model. **a** Experimental design of lung orthotopic tumor development and treatment of PBS, A5 Nb (5 mg/kg), LNP-DOX (4 mg/kg), and A5-LNP-DOX (4 mg/kg). **b** Tumor growth was assessed by bioluminescence on days 7, 15, 20 and 25 after A549-luc cell injection into the lung left lobe. The bioluminescence intensity is expressed in photons/s for the thoracic area. Data were expressed as mean ± SEM. The sample size is *n* = 5. *: *vs*. PBS, ***p < 0.001, ^#^: *vs*. A5-LNP-DOX, ^###^*p* < 0.001. **c** H&E staining image of tumors from PBS-, A5 Nb-, LNP-DOX-, and A5-LNP-DOX-treated group. Scale bar = 150 μm. The black dashed lines in the H&E stained tissue indicate the tumor-formed regions. The bar graph showed % of tumor regions in lung tissues from PBS-, Ctrl Nb-, A5 Nb-, LNP-DOX-, and A5-LNP-DOX-treated group. *n* = 5. **d** Active-caspase-3 and luciferase staining of tumors from PBS-, Ctrl Nb-, A5 Nb-, LNP-DOX-, and A5-LNP-DOX-treated group. The bar graph showed % of active-caspase3 in luciferase positive cells. For statistical analysis, five randomly selected tumors per group were stained and five random fields per tumor were scored. Scale bar = 150 μm, Data represented as mean values ± SD, determined by two-tailed Student’s *t*-test. *: *vs*. A5-LNP-DOX, *p < 0.05 **p < 0.01 and ***p < 0.001. The mouse icon of (**a**) created by BioRender

To assess the antitumor efficacy of A5-LNP-DOX in physiologically relevant lung microenvironment, we performed an orthotopic lung cancer model using luciferase-expressing A549 cells. Tumor progression was monitored over 25 d by bioluminescence imaging (BLI), and mice were treated intravenously with PBS, A5 Nb, LNP-DOX, or A5-LNP-DOX (DOX-equivalent dose = 4 mg/kg) three times per week (Fig. 5a). Following treatment, compared to PBS, all treatment groups including A5 Nb, LNP-DOX, and A5-LNP-DOX showed reduced tumor growth as indicated by serial BLI measurements. Notably, A5-LNP-DOX exhibited the most pronounced tumor suppression, with bioluminescence intensity significantly lower than that of both A5 Nb and LNP-DOX monotherapies, confirming its superior antitumor efficacy (Fig. 5b).

At day 25, lungs were harvested from all experimental groups for histological evaluation. H&E staining revealed extensive tumor infiltration in the PBS group, with dense tumor regions occupying ~40% of the total lung area. In contrast, tumor burden was substantially reduced in the A5 Nb and LNP-DOX groups, with tumor areas of 13% and 27%, respectively. Notably, A5-LNP-DOX treatment resulted in the lowest tumor burden, with only 4% of the lung occupied by dense tumor tissue (Fig. 5c). To assess treatment-induced apoptosis, active caspase-3 staining was performed on Luc-expressing tumor cells. The A5-LNP-DOX group exhibited significantly higher levels of caspase-3 positive cells compared to all other groups, supporting the enhanced apoptotic response induced by A5-LNP-DOX (Fig. 5d). These findings support A5-LNP-DOX as an effective CD155-targeted therapy with superior antitumor activity in lung cancer.

### Antitumor effects of A5-LNP-DOX in human lung cancer organoid xenograft models

**Fig. 6:**
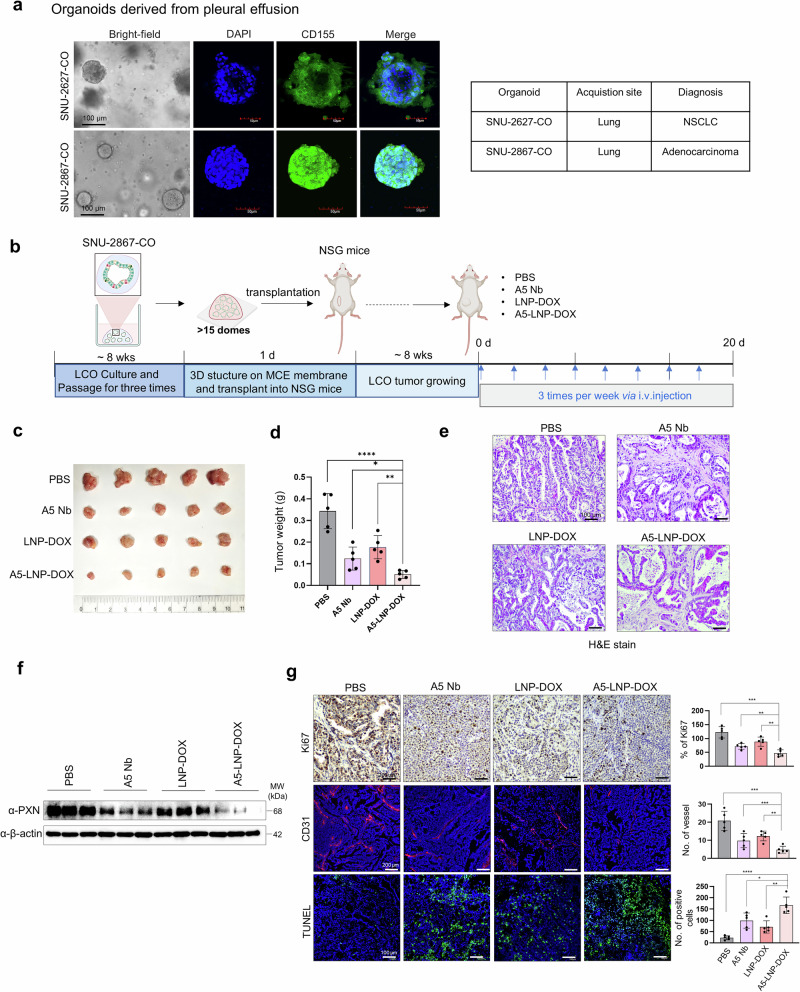
Xenograft mouse model used LCOs. **a** Bright-field microscopy images of SNU-2627-CO and SNU-2867-CO. Scale bar = 100 μm. The information of LCOs; SNU-2627-CO;non-small cell lung carcinoma, SNU-2867-CO; lung adenocarcinoma. **b** Experimental design of LCO culture, 3D structure on MCE membrane and an antitumor effect in vivo was observed for PBS, A5 Nb (5 mg/kg), LNP-DOX (4 mg/kg), and A5-LNP-DOX (4 mg/kg). **c**, **d** Images of LCO tumors harvested at the endpoint and tumor weights. Data were expressed as mean ± SEM. The sample size is *n* = 5. *: *vs*. A5-LNP-DOX, *p < 0.05, **p < 0.01, and ****p < 0.0001. **e** H&E staining image of LCO tumors from PBS-, A5 Nb-, LNP-DOX-, and A5-LNP-DOX-treated group. Scale bar = 100 μm. **f** Paxillin expression (**g**). Ki67 IHC (**g** top) and CD31 IHC (**g** middle) staining of tumors from PBS-, Ctrl Nb-, A5 Nb-, LNP-DOX-, and A5-LNP-DOX-treated group. Detection of apoptosis by TUNEL assay (**g** bottom) in tumor tissue. For statistical analysis, five randomly selected tumors per group were stained and scored. Scale bar = 200 μm (Ki67 and CD31) and 100 μm (TUNEL) for (**g**). Data represented as mean values ± SD, determined by two-tailed Student’s *t*-test. *: *vs*. A5-LNP-DOX, *p < 0.05, **p < 0.01, ***p < 0.001, and ****p < 0.0001. The LCO and mouse icon of (**b**) created by BioRender

Patient-derived lung cancer organoids (LCOs), SNU-2627-CO and SNU-2867-CO, were generated from malignant pleural effusion (MPE) samples of pathologically confirmed NSCLC patients^35^ and utilized to establish xenograft models. Immunofluorescence analysis revealed that SNU-2867-CO exhibited markedly higher CD155 expression than SNU-2627-CO, validating it as a suitable LCO for CD155-targeted therapy (Fig. 6a).

To evaluate in vivo efficacy, SNU-2867-CO organoids were transplanted into NSG mice and treated with PBS, A5 Nb, LNP-DOX, or A5-LNP-DOX. After 8 weeks, the A5-LNP-DOX group showed the most pronounced tumor suppression. Tumor weights in the A5-LNP-DOX group were ~42%, 64%, and 75% lower compared to those in the LNP-DOX, A5 Nb, and PBS groups, respectively (Fig. 6b–d). These results underscore the superior antitumor efficacy of A5-LNP-DOX over both monotherapies and the untreated control. Histological analysis of LCO-derived tumors by H&E staining confirmed that the xenografts retained key pathological features of lung adenocarcinoma, including acinar and lepidic growth patterns.^36,37^ These architectural features closely resembled those observed in LUAD patient tissues, supporting the clinical relevance of the LCO-based model. In contrast, tumors from the A5-LNP-DOX group exhibited a marked disruption of these structures, indicating that treatment significantly impaired tumor morphology (Fig. 6e).

Consistent with in vitro findings, A5 Nb- and A5-LNP-DOX treated LCO tumors exhibited reduced PXN expression compared to PBS controls, as shown by western blot analysis (Fig. 6f). Moreover, A5-LNP-DOX treatment markedly suppressed tumor cell proliferation and angiogenesis, as indicated by a decrease in Ki67-positive cells and reduced CD31-labeled microvessel density (Fig. 6g, top and middle, respectively). Notably, TUNEL staining demonstrated a ~ 7-fold increase in apoptotic tumor cells in the A5-LNP-DOX group compared to PBS, highlighting apoptosis as a key mechanism underlying its therapeutic effect (Fig. 6g, bottom). We believe that A5-LNP-DOX as a clinically relevant and promising therapeutic approach with strong potential for further development in CD155-overexpressing lung cancer.

### CD155-targeting nanobody-liposomes suppress tumor growth without systemic toxicity

To complement the findings from the orthotopic and organoid models, we further evaluated A5-LNP-DOX in a subcutaneous A549 xenograft model. Consistent with previous results, A5-LNP-DOX treatment significantly suppressed tumor growth and reduced PXN expression, cell proliferation, and microvessel density (Supplementary Fig. 9a–e).

To further evaluate the biosafety of CD155-targeted nanobody-based therapies, systemic toxicity was assessed across all treatment groups. As shown in Supplementary Fig. 10a, there were no significant differences in body weight among the PBS, Ctrl Nb, A5 Nb, and LNP-DOX groups. A5-LNP-DOX treated mice exhibited a modest ( ~ 10%) reduction in body weight compared to PBS controls; however, this change was transient and not associated with signs of distress or morbidity. Nevertheless, there were no significant differences in serum GOT (AST) and GPT (ALT) levels among groups, including A5-LNP-DOX, indicating maintained liver function (Supplementary Fig. 10b). Histopathological examination of major organs, including the lungs, liver, kidneys, spleen and heart, revealed no evidence of tissue damage or inflammation in any treatment group (Supplementary Fig. 10c), further supporting the absence of systemic toxicity.

Collectively, these results demonstrate that A5-LNP-DOX delivers potent antitumor effects without inducing organ toxicity or compromising physiological integrity. The selective delivery of doxorubicin *via* CD155-targeted nanobody-liposomes enables effective tumor suppression while maintaining an excellent safety profile in vivo.

## Discussion

Recent studies have shown that the interaction between CD155 and TIGIT is a pivotal regulator of immune responses, exerting inhibitory effects on T cell activation, cytokine production, and cytotoxicity, thereby facilitating immune evasion by tumors. Within the CD155/TIGIT trans-dimer complex, specific sequence motifs, such as the AX6G motif (residues 76–83 in CD155, 66–74 in TIGIT) located in the C’C” loop, and the T(F/Y)P motif in the FG loop (residues 127–129 in CD155 and 112–114 in TIGIT), orchestrate characteristic lock-and-key interactions.^38^ The concave “lock” is formed by the AX_6_G motif in the C’C” loop, creating a hydrophobic pocket, while the convex “key” consists of an aromatic residue within the FG loop (F128 in CD155 and Y113 in TIGIT), which securely engages with the hydrophobic lock pocket on the opposing molecule.^39,40^ Therapeutic blockade of this pathway using monoclonal antibodies has recently gained traction. NTX-1088, a first-in-class anti-CD155 antibody currently under evaluation in a Phase I clinical trial (NCT05378425), has been reported to enhance immune activation and promote immune-mediated tumor cell killing both in vitro and in vivo.^41,42^ However, the mechanism by which NTX-1088 regulates TIGIT, CD96, and KIR2DL5A remains unclear, and the antibody concentration used for CD155 blockade in these studies has not been disclosed, making it difficult to fully evaluate its therapeutic efficacy.

In contrast to conventional antibodies, nanobodies (Nbs) offer several advantages for clinical translation, including small size, high solubility, robust tissue penetration, and ease of large-scale production.^43,44^ In this study, we developed a high-affinity nanobody (A5 Nb) from a synthetic phage display library and demonstrated its ability to modulate tumor phenotypes through inhibition of focal adhesion signaling. A5 Nb significantly inhibited migration and invasion of CD155-overexpressing lung cancer cells at concentrations more than tenfold lower than those required for conventional antibodies, suggesting superior potency and potential for safer dosing strategies (Supplementary Fig. 3, 4). Notably, A5 Nb achieved over 80% cellular uptake in A549 cells, far surpassing 25% uptake of a conventional CD155 antibody (Supplementary Fig. 3a, b), underscoring its superior efficiency for targeted drug delivery. Mechanistically, A5 Nb treatment markedly downregulated PXN, a key focal adhesion scaffold protein, and reduced its localization at nascent adhesions, thereby impairing cell migration and cytoskeletal dynamics. These effects were specific to CD155-high cells and were not observed in CD155-low cells. Furthermore, patient tissue analyses revealed that co-overexpression of CD155 and PXN was associated with poor prognosis in LUAD, underscoring the clinical relevance of the CD155-PXN axis (Fig. 3). Although CD155 has been reported to regulate tumor progression through several oncogenic pathways, including the AKT/mTOR and MEK/ERK pathways in specific cancer types such as cervical and colorectal cancers,^18,32^ our findings suggest that CD155 may also function through non-canonical and context-dependent signaling mechanisms. In our study, A5 Nb treatment did not alter ERK or AKT phosphorylation but significantly reduced PXN expression in CD155-overexpressing lung cancer cells (Supplementary Fig. 6a). Consistently, in hepatocellular carcinoma (HCC) models, modulation of CD155 had no effect on ERK activation but led to changes in p38 MAPK signaling.^45^ In contrast, in glioma cells, siRNA-mediated knockdown of CD155 did not affect ERK or AKT phosphorylation but specifically disrupted Src/PXN signaling.^17^ These findings indicate that CD155-driven signaling may vary across tumor types and involve alternative regulatory networks beyond the canonical AKT/ERK signaling.

To enhance therapeutic efficacy, A5 Nb was conjugated to doxorubicin-loaded liposomes (A5-LNP-DOX) for targeted drug delivery. A5-LNP-DOX accumulated in the tumor interstitial space and demonstrated potent antitumor activity in both in vitro and in vivo settings. Beyond conventional subcutaneous xenograft models, its efficacy was further validated in two advanced systems: a lung orthotopic model that recapitulates the native tumor microenvironment and a patient-derived lung cancer organoid (LCO) xenograft model that reflects clinical tumor heterogeneity. Notably, A5-LNP-DOX consistently exhibited strong therapeutic effects across all models. These findings support its clinical relevance and highlight A5-LNP-DOX as a promising nanobody-based drug delivery platform with favorable biocompatibility and efficacy against CD155-positive tumors. A notable limitation of our study is that the organoid xenograft model was established using only one patient-derived lung cancer organoid. Although this model successfully retained key histological and functional features of human tumors and offered valuable insight into the clinical applicability of A5-LNP-DOX, the use of a single organoid line may not adequately reflect the inter-patient variability commonly observed in lung cancer. This constraint should be considered when interpreting the efficacy data obtained from the organoid model, as tumor response may differ in organoids derived from other patients with diverse genetic backgrounds or molecular subtypes.

Although our preclinical assessments, including tissue histology and blood analyses, indicated that both A5 Nb and A5-LNP-DOX are safe, A5 Nb does not bind to murine CD155 (Supplementary Fig. 11). Therefore, future studies employing human CD155 transgenic (hCD155-Tg) models would be valuable for more accurately evaluating potential on-target, off-tumor effects in vivo. Another theoretical limitation of targeting CD155 is the presence of tumor-derived soluble CD155 (sCD155), which could act as a decoy and potentially reduce therapeutic efficacy. To address this, we analyzed CD155 isoform expression in CD155-high cell lines and found that membrane-bound CD155α was predominantly expressed, whereas soluble isoforms (CD155β and γ)^16^ were minimal (Supplementary Fig. 12). While sCD155 expression was negligible in our models, its potential role in modulating therapeutic efficacy in select clinical scenarios cannot be entirely ruled out and may warrant consideration in future translational studies. In preparation for clinical application, further studies should also address pharmacokinetics, immunogenicity, and scalable manufacturing of A5-LNP-DOX. The successful use of A5-LNP-DOX demonstrates the potential of targeted drug delivery systems in improving therapeutic outcomes. Clinical trials are needed to assess the efficacy and safety of A5-LNP-DOX in diverse cancer types and patient populations. Additionally, exploring the impact of CD155 and PXN expression on tumor progression could provide further insights into developing more effective treatment strategies.

## Material and method

### Library construction

The synthetic nanobody library used a humanized nanobody scaffold (hNbBcll10FGLA), and was produced through a CDR randomization process. The lengths of CDR1 and CDR2 were fixed at 9 and 6 amino acids, respectively, whereas CDR3 maximized diversity with a length of 6–25 amino acids. A synthetic nanobody library was prepared using cloning. The pkb-nanobody phaid vector (KRIBB-nanobody vector) was cut using restriction enzymes ApaI (New England Biolabs, MA, USA) and HindIII-HF (New England Biolabs), and then synthetic VHH genes were inserted. After cloning, the recombinant plasmids were transformed by electroporation (voltage 2.5 kV, resistance 200 Ω, capacitance 25 μF) in the E. Coli strain TG1. The diversity of the libraries was verified using next-generation sequencing (NGS). Analysis of individual nanobody sequences revealed that ~93.6% were distinct and non-redundant.

### Purification of nanobodies

The VHH gene was cloned into the TGEX-SCblue plasmid (Antibody Design Lab, CA, USA) and expressed in Expi293F cells. Transient transfection was performed using 293fectin reagent, following the manufacturer’s instructions, and cells were incubated at 37 °C with 5% CO_2_. After 120 h, supernatants were collected by centrifugation at 3,000 × g for 10 min, filtered through a 0.22 μm membrane, and stored at 4 °C. Nanobody purification was performed using Protein A agarose resin, with elution carried out using 0.1 M glycine buffer (pH 2.7), followed by immediate neutralization with 1 M Tris-HCl (pH 8.0). The molecular weight and purity of the nanobodies were evaluated by SDS-PAGE under reducing conditions, followed by protein staining.

Silver staining kits (Thermo Fisher Scientific, MA, USA) and Coomassie Brilliant Blue R-250 Staining Solution kits (Bio-Rad, CA, USA) were used for visualizing protein bands on SDS-PAGE gels. Proteins (0.5 ng per sample) were loaded onto SDS-PAGE gels and electrophoresed at 120 V for 1 h. After electrophoresis, the gels were carefully separated and subjected to silver staining or Coomassie staining according to the manufacturers’ protocols.

### Screening of antibody against CD155 antigen

A nanobody synthetic library containing 10^11^ uniquely mutated sequences from three CDRs was used for nanobody selection. Three rounds of biopanning were performed as described previously.^46,47^ Briefly, 100 μg/mL of purified CD155 antigens were coated on an immune tube for overnight at 4 °C and blocking was performed with 5% skim milk (Thermo fisher scientific, MA,USA) in Dulbecco’s phosphate buffered saline (DPBS) for 1 h at 37 °C. 1 mL of nanobody phage libraries (10^11^ ~ 10^12^ PFU/mL) was added blocked immune tube for 2 h at 37 °C binding. The CD155-coated tube was washed 10 times with DPBS-Tween20 (PBST, 0.05%), and the remaining phage was eluted with 1 mL of 100 mM trimethylamine (Sigma Aldrich, MO, USA), followed by 10-min incubation. The eluted phage was neutralized 1 M Tris-HCl (pH 7.5) and infected to 10 mL of *E. coli* strain TG1 cell (OD_600_ = 0.6) and incubated for 30 min at 37 °C. With the increase in the number of pannings, the number of washings was increased by 10 times, though the experimental method was same. Clones amplified in the 3^rd^ panning were randomly selected and single colonies were cultured to OD_600_ = 0.6, incubated with IPTG and cultured at 28 °C for 12 h. The supernatant was subjected to indirect ELISA to select CD155-specific nanobody colonies by measuring absorbance at 450 nm using a microplate reader (Tecan for Life Sciences, Mannedorf, Switzerland).

### Preparation of liposome

1,2-Dipalmitoryl-sn-glycero-3-phosphocholine (DPPC), cholesterol (Chol), 1,2-distearoyl-sn-glycerol-3-phosphoethanolamine-N-[methoxy(polyethylene glycol)-2000] (DSPE-PEG2000) and 1,2-distearoyl-sn-glycero-3-phosphoethanolamine-N-[maleimide(polyethylene glycol)-2000] (DSPE-mal) were purchased from Avanti Polar Lipids and used without further purification. N-succinimidyl-S-acetylthioacetate (SATA, Thermo fisher scientific) was purchased from Thermo Fisher Scientific. Liposomes were prepared using a conventional thin-film hydration method. Various lipids (DPPC:Chol:DSPE-PEG2000:DSPE-mal) with a molar ratio of 75:20:4.5:0.5 were dissolved in chloroform in a dry round-bottom flask. The solvents were evaporated using rotary evaporation for 60 min. The resulting thin lipid film was hydrated using 250 mM ammonium sulfate buffer and hydrated at 60 °C for 30 min, followed by five cycles of freezing and thawing. The large vesicles were extruded through 50 nm polycarbonate membranes using an Avanti mini-extruder for 21 cycles. Then, a pH gradient was created through the removal of extra-liposomal ammonium sulfate salts by 30 kDa molecular weight cutoff (MWCO) centricon. The pH gradient served to actively load DOX (20 mg/mL) at 65 °C for 2 h.

### Conjugation of nanobody to liposome

The A5 nanobody was modified with SATA at a molar ratio 1:8 (A5 Nb:SATA), followed by deacetylation to generate free thiol groups. Deacetylation was performed by incubating the STAT-derivatized Nb with hydroxylamine solution (0.5 M NH_2_OH, 0.5 M HEPES, 25 mM EDTA, and pH 7.2–7.5) to achieve a final hydroxylamine concentration of 0.05 M for 2 h. The sulfhydryl content of the deacetylated derivatives was quantified using the Ellman’s reagent method. SATA derivatized and deacetylated Nbs were incubated with liposomes overnight at 4 °C. Unbound nanobodies were removed by ultrafiltration using an Amicon Ultra 100 kDa centrifugal filter unit. To confirm the conjugation of Nbs to liposomes, a micro bicinchoninic acid (BCA) assay was performed on diluted liposomes. The micro-BCA assay was used to measure the Nb concentration of the functionalized liposomes; for these experiments, the Nb-functionalized liposome stocks were diluted by a factor of 20 to 200 in PBS, and the micro-BCA assay was performed to measure the protein mass concentration in the diluted liposome samples. To account for the low contribution of lipids to the micro-BCA assay, we performed micro-BCA measurements on non-functionalized DSPE-PEG-maleimide-containing liposomes. The contribution of lipids was subtracted to determine the true number of Nbs conjugated to the liposomes.

### Characterization of liposome

The particle size and polydispersity index were measured by dynamic light scattering (DLS) using a Zetasizer (Zetasizer Ultra-Red, Malvern Instrument Ltd., Worcestershire, UK). The samples were adequately diluted, and the experiments were performed at 25 °C. A minimum of three different batches were assessed to determine the average value and standard deviation of the particle diameter and zeta potential.

The morphological characteristics of the drug-loaded nanoparticles were determined using transmission electron microscopy (TEM).^48^ Briefly, the nanoparticle dispersion was diluted, and a drop was placed on a carbon-coated copper grid. The particles were stained with 2% phosphotungstic acid (PTA). The particles were then dried using an infrared lamp (IR) and viewed under a microscope.

High-performance liquid chromatography (HPLC) was used to identify and quantify DOX in liposomal formulations. Purified liposomes were lyophilized, dispersed in 0.5 mL of acetonitrile, and incubated in cold water for 2 h. The suspension was diluted with an equal volume of DI water and centrifuged at 24,104 × g for 20 min to obtain a clear supernatant. The supernatant was filtered through a 0.2 μm syringe filter and analyzed by HPLC. HPLC analysis was performed by the Agilent 1260 system, equipped with a C18 column (25 cm × 4.6 mm, particle size: 5 μm). DOX was eluted with a 90:10 mixture of water and acetonitrile at a flow rate of 1 mL/min and detected at 269 nm.

$${\rm{Drug\; Loading\; Capacity}}( \% )=\frac{{\bf{Amount\; of\; drug\; in\; liposome}}}{{\bf{Total\; amount\; of\; liposomes}}}\times 100$$

$${\rm{Encapsulation\; efficiency}}\left( \% \right)=\frac{{\bf{Amount\; of\; entrapped\; drug}}}{{\bf{Amount\; of\; drug\; added\; initialy}}}\times 100$$

### In vitro drug release study

Liposomes equivalent to 115 μg/mL of DOX was placed in Float-A-Lyzer G2 dialysis device with a MWCO 3.5 kDa. The device was incubated in 20 mL of PBS (pH 7.4) or sodium acetate (pH 5.2) at 37 °C with constant agitation. At predetermined time points, 0.8 mL of the release medium was sampled and replaced with a fresh buffer. The sampled buffer was filtered with a syringe filter (0.22 μm pore size) and analyzed by HPLC.

### Internalization assay and cellular uptake

A549 cells were incubated with DOX loaded LNP and A5-LNP at 37 °C for 4 h Following incubation, cells were washed with PBS and fixed with 4% paraformaldehyde. The cells were stained with DAPI (Invitrogen, CA, USA). All fluorescent images were acquired *via* the utilization of Floview confocal microscopy (Olympus, MA, USA). The cells were treated with LNP and A5-LNP under identical conditions to those described above. After treatment, the cells were washed and harvested, followed by fluorescence quantification using a flow cytometer (BD Accuri C6; BD Bioscience, CA, USA).

### Development of Orthotopic Lung Cancer Model

Twenty 6–7 week-old NOD-scid gamma (NSG) mice (The Jackson Laboratory, ME, USA) were used for all experiments and housed in a pathogen-free animal facility at the Korea Research Institute of Bioscience and Biotechnology (KRIBB). Mice were maintained on a 12 h light/dark cycle with ad libitum access to food and water. The mouse was positioned with the left lung facing upward and the area was shaved using a 0.5 mm beard shaver under isoflurane anesthesia (Butler Schein, OH, USA). After the surgical site was sterilized using 70% isopropyl alcohol, a 1 cm incision was made on the left side of the chest, followed by dissection of the soft tissue to expose the thoracic ribs and intercostal space. Ten microliters of 1 × 10^6^ A549-luc cells were drawn into a 29 G 1/2” insulin syringe and the needle was swiftly advanced to a depth of ~5 mm into the left lung lobe between the 6th and 7th ribs, and the cell suspension was injected. The incisions on the chest, muscle, and skin were then sutured closed in layers. Post-surgery, the mice were placed under a heat lamp for 2 to 5 min to warm up until they regained consciousness and displayed normal activity and functionality, after which they were returned to their original cages. Mice were monitored daily for any visible signs and symptoms, including bradypnea, tachypnea, reduced movement, weight loss, and wound dehiscence. Tumor growth was assessed by bioluminescence.

### Lung cancer organoid cultures and passaging

SNU-2627-CO and SNU-2867-CO was established from malignant pleural effusion derived from non-small cell lung cancer.^35^ LCOs (Lung cancer oragnoids) were purchased from Korean Organoid biobank (Seoul, Republic of Korea). The oragnoid culture medium information are provided in Supplementary Methods. The culture medium was refreshed every two to 3 days depending on growh rate. To passage the organoids, the BME dome was broken up using TrypLE Express (GIBCO) and pipetted into a 15 mL conical tube. The dome was further disrupted by forceful pipetting, then incubated at 37 °C for 10 min. Subsequently, three volumes of basal medium containing 10% FBS were added to the suspension. The domes were centrifuged at 10 × g for 3 min, and the supernatant was removed. Once BME was removed, the dissociated organoid pellet was resuspended in basal medium + Matrigel (1:3) and resseded at 1:3–1:4 rations to allow the formation of new oragnoids. The new dome (50 μL) was seeded in 6 well plate and incubated at 37 °C for 10 min. After the dome had solidified, 3 mL of the culture medium was gently added to the each well to cover the domes and organoids were incubated in a 37 °C and 5% CO_2_ incubator.

### Human lung cancer organoid xenograft tumor model

To engraft LCOs per one-mouse, LCOs cultured in 2 wells of 6-well plates over 2 weeks (dimeter of LCO reached up 100–150 μm) were havested and separated from Matirgel by centrifugation at 250 × g for 30 min at 4 °C. To maintain the LCOs with 3D strucuture, the LCOs separated from Matrigel were resuspended in 50 μl new Matrigel. The Matrigel containing LCOs was solidified on mixed cellulose ester membranes (9 μm pore, Millipore, MA, USA) for 10 min.^49^

Thirty 6–7-week-old NSG mice (The Jackson Laboratory) were used for all experiments and housed in a pathogen-free animal facility at the KRIBB. The mouse was positioned with the dosal facing upward and the upper left thigh area was shaved using a 0.5 mm beard shaver under isoflurane anesthesia (Butler Schein). After the surgical site was sterilized using 70% isopropyl alcohol, a 1 cm incision was made on the upper left thigh of the dorsal side, followed by dissection of the skin and insertion of the solidified LCOs membrane. The membrane is positioned facing the outer skin. After suturing, the mice were placed under a heat lamp for 5 min to warm up until they regained consciousness and displayed normal activity and functionality, after which they were returned to their original cages. Tumor formation is monitored for ~3 months.

### Ethics

All experimental procedures involving human-derived materials and animals were conducted in accordance with institutional guidelines. The use of human-derived materials, including commercially obtained cell lines and tissue microarrays, was approved for exemption from IRB review by the Korea Research Institute of Bioscience and Biotechnology (IRB No. P01-202408-02-007). All animal experiments, including housing and care, were approved by the KRIBB Institutional Animal Care and Use Committee (IACUC No. KRIBB-AEC-23030) and conducted in full compliance with its ethical guidelines.

### Statistical analysis

Data are displayed as the mean ± SEM or mean ± SD and were analyzed with Prism version 10.4.0 (GraphPad Software, Inc., San Diego, CA, USA). The unpaired Student’s t-test was used for comparisons between two groups. We used one-way ANOVA with Tukey’s post-hoc tests to compare multiple groups. A *p*-value < 0.05 was considered significant.

## Supplementary information

Supplementary_Materials_

WB uncut

## Data Availability

All data supporting the findings of this study are available within the article and its Supplementary Information files.
