# Supplementary material for: Targeting CD155 in lung adenocarcinoma: A5 nanobody-based therapeutics for precision treatment and enhanced drug delivery
Source: Signal Transduct Target Ther. 2025 Jul 10;10:218. doi: 10.1038/s41392-025-02301-z (PMC12241526; doi:10.1038/s41392-025-02301-z)
Supplement: Supplementary file 1 — Supplementary_Materials_ [file 41392_2025_2301_MOESM1_ESM.docx]

Supplementary Materials for

Targeting CD155 in lung adenocarcinoma: A5 nanobody-based therapeutics for precision treatment and enhanced drug delivery

Kyunghee Noh, Soyeon Yi, Hyeran Kim, Jieun Lee, Suhyeon Kim, Wonbeak Yoo, Eunkyeong Jung, Jinsol Choi, Hwangseo Park, Seungha Hwang, Jin Young Kang, Kwang-Hyun Park, Heewon Park, Yong-kyu Lee, Eun-Kyung Lim, Taejoon Kang, Juyeon Jung

Correspondence to: jjung@kribb.re.kr

**This PDF file includes:**

Materials and Methods

Figures. S1 to S12

Tables S1 to S4

Materials and Methods

**Enzyme-linked immunosorbent assay (ELISA)**

Immuno microwells (Thermo Fisher Scientific, MA,USA) were coated with the purified antigen (100 ng/well) in 50 mM sodium carbonate buffer (pH 9.6) at 4 °C overnight, blocked with skim milk (4%) in PBS and washed with PBST (0.05% Tween-20 in PBS). The Immuno 96-well plate was washed with PBST, followed by nanobody cross-adsorption and incubation at 37 °C for 1 h. The plates were washed with PBST, goat anti-human IgG (Fab specific) antibody (Sigma Aldrich, MO, USA) was added, incubated at 37 °C for 1 h, washed with PBST, and treated with HRP conjugated anti-goat antibody. After incubation for 1 h at 37 °C, the plate was washed with PBST and color was developed with the addition of 100 μL of TMB solution and 50 μL of H_2_SO_4_ (1 M). The absorbance at 450 nm was measured using a microtiter plate reader (Tecan for Life Sciences, Mannedorf, Switzerland). Affinity was determined as the antigen concentration required to inhibit 50% of binding activity, and binding affinity (*K*_d_) value was calculated using the PRISM software.

Data acquisition and processing

Gene Expression Profiling Interactive Analysis (GEPIA) (http://gepia2.cancer-pku.cn/) is a developed interactive web server for estimating the RNA sequencing expression data, based on 9,736 tumors and 8,587 normal samples from the Cancer Genome Atlas (TCGA) and Genotype-Tissue Expression (GTEx) dataset projects. This database was used to assess gene correlation based on the expression levels of CD155, and PXN.

**OncoLnc database analysis**

Clinical and transcriptomic data for lung adenocarcinoma (LUAD) patients were obtained from the OncoLnc database (<https://www.oncolnc.org>), which integrates The Cancer Genome Atlas (TCGA) datasets. Patients were stratified into two groups based on PXN and CD155 expression levels using the median expression value as the cutoff (top 50% vs bottom 50%). Overall survival data were extracted and analyzed using GraphPad Prism (version 10.4.0). Kaplan–Meier survival curves were plotted, and statistical significance between groups was determined using the log-rank (Mantel–Cox) test. Raw values and survival metadata used for this analysis are provided in Supplementary table 2.

Microarray data

Gene expression profiling datasets, including GSE116959 (Normal lung tissue; n = 10, lung cancer tissue; n = 57), GSE19188 (Normal lung tissue; n = 65, lung cancer tissue; n = 91), and GSE32863 (Normal lung tissue; n = 58, lung cancer tissue; n = 58)were downloaded from the Gene Expression Omnibus (GEO, <https://www.ncbi.nlm.nih.gov/geo/>).

Immunohistochemistry staining

CD155 protein expression in lung cancer and normal lung tissues were verified by immunohistochemistry using the Human Tissue Atlas, an online tool for genome-wide analysis (<http://www.proteinatlas.org/>)^1^.

Cell lines and cell culture

The human lung cancer cell lines (BEAS-2B, A549, H441, H460, and H520) were obtained from the American Type Culture Collection (ATCC, VA, USA), and cultured in RPMI 1640 medium (RPMI) supplemented with 10% fetal bovine serum (FBS), 100 μg/mL penicillin and 100 μg/mL streptomycin in a humidified incubator at 37°C under a 5% CO_2_ atmosphere.

Surface plasmon resonance (SPR) analysis

Binding affinity was determined using a Biacore T200 instrument (Cytiva, MA,USA) with a CM5 chip and HBS-EP+ buffer. Nanobodies were immobilized *via* standard amine coupling, and serially diluted antigens (0–500 nM) were injected to assess association and dissociation. Kinetic parameters were fitted using a 1:1 Langmuir binding model via the Biacore T200 Evaluation Software. Experimental procedures adhered to previously established protocols.^2^

**Homology modeling of A5 Nb structure and docking simulations with CD155**

Since no structural data were available for A5 Nb, its three-dimensional (3D) structure was generated through homology modeling. The process began by using the amino acid sequence of A5 Nb as input for a BLAST search (https://blast.ncbi.nlm.nih.gov/Blast.cgi) to identify structural homologs. This search led to the discovery of a humanized camelid single-domain antibody with a known three-dimensional structure, available in the Protein Data Bank under the accession code 3EAK,which was used as the structural template for modeling the A5 Nb. The accuracy of the A5 Nb structural model was ensured by the high degree of amino acid sequence identity (77%) between the target and the chosen template, enabling the generation of a reliable and high-quality 3D structure.^3^ The latest version of the MODELLER software (<https://salilab.org/modeller/>) was used for homology modeling. Structural optimization was performed using the conjugate gradient method, followed by molecular dynamics simulations to minimize violations of spatial restraints. The three-dimensional atomic coordinates of gap regions were modeled from a randomly distorted structure spanning the two anchoring points. The finalized structural model of A5 Nb was subsequently used to investigate its potential binding interactions with CD155 through protein-protein docking simulations. The finalized structural model of A5 Nb was subsequently employed to investigate potential binding interactions with CD155 using protein–protein docking simulations.

To explore the binding modes between A5 Nb and CD155, we performed protein-protein docking simulations. The receptor model was derived from the X-ray crystal structure of CD155 in complex with the first immunoglobulin domain of CD96 (PDB entry: 6ARQ).^4^ In these simulations, the CD155 structure was held rigid throughout the docking process, while the homology-modeled structure of A5 Nb was used as the flexible ligand. This approach allowed the A5 Nb model to explore various orientations and conformations to identify the most favorable binding configurations relative to the fixed receptor. All docking simulations were carried out using the RosettaDock program,^5^ employing a multi-scale Monte Carlo-based algorithm. Initially, A5 Nb was overlaid onto the immunoglobulin domain of CD96 within the CD155-CD96 complex structure. The orientation of A5 Nb relative to CD155 was optimized by rigid-body translation and rotation, followed by sidechain refinement using rotamer packing and gradient-based minimization. The binding energy calculation utilized a multifaceted scoring function that included van der Waals interactions, electrostatics with reduced weighting, implicit Gaussian solvation, orientation-specific hydrogen bonding, and side-chain rotamer probability terms.^6^ From the 1,000 binding poses generated by docking simulations, the conformation exhibiting the lowest binding energy was chosen as the final structure of the A5 Nb–CD155 complex.

Apoptosis and Live/Dead cell staining

A549, H441, and BEAS-2B cells (1 × 10^6^ cells/well) were seeded in a 100 mm cell culture dish. After 1 d, LNP-DOX (10 μg/mL) and A5-LNP-DOX (10 μg/mL) were treated. To visualize cell viability, cells were stained with calcein-AM and propidium (PI) solution using a live/dead staining kit (Thermo Fisher Scientific), and then fluorescence images were confirmed.

Real-time quantitative reverse transcription-polymerase chain reaction (qRT-PCR) analysis

Quantitative real-time reverse-transcription polymerase chain reaction (qRT-PCR) was performed using 50 ng of total RNA isolated from cells using the RNeasy Mini Kit (Qiagen, MD, USA). Complementary DNA (cDNA) was synthesized from 0.5-1.0 μg of total RNA using a Verso cDNA kit (Thermo Fisher Scientific). qPCR analysis was performed in triplicate using the reported primers (Supplementary Table 3) and Power SYBR Green PCR Master Mix (Applied Biosystems, MA, USA) with Applied Biosystems QuantStudio 1 (Applied Biosystems). Quantification was performed by using the 2^−ΔΔCT^ method normalizing to control for percent fold changes.

Western blotting

Cells were lysed in RIPA buffer (50 mM Tris-HCl, pH 8.0, 150 mM NaCl, 5 mM EDTA, and 0.1% SDS), and the cell debris was cleared by centrifugation at 15,000 ×g for 10 min. The lysates were boiled in SDS sample buffer for 5 min. Proteins were resolved using sodium dodecyl sulfate-polyacrylamide gel electrophoresis (SDS-PAGE) and transferred onto a polyvinylidene fluoride membrane (Millipore, MA, USA). The membranes were blocked with 5% skim milk in TBST (0.5% Tween-20 in Tris-buffered saline) at 25 °C for 1 h. The membranes were incubated with the appropriate antibodies in 5% skim milk either for 1 h at 25 °C or overnight at 4°C, and then with secondary antibody in TBST for 1 h at 25 °C. Proteins were detected using a chemiluminescence kit (Intron Biotech, Seoul, Republic of Korea and Millipore).

Flow cytometry analysis

All flow cytometry experiments were performed at 4°C. Cells were blocked with PBS/2% BSA solution for 30 min to avoid non-specific binding. Nbs (10 µg/mL), CD155 antibody (Invitrogen #MA5-13493), or CD155-PE antibody (BioLegend, Cat# 337507) were added to cells in PBS/2% BSA and incubated for 30 min. After three washes with PBS/2% BSA, cells were incubated for 30 min with secondary antibodies. After washing thrice in PBS, binding was detected by flow cytometry (BD FACSCalibur, BD Biosciences, NJ, USA). Control Nb (Ctrl Nb) and mouse IgG served as a negative control. Data were analyzed using Cell Quest Pro software (BD Biosciences). Goat Anti-human IgG FC-FITC antibody was used for Nbs, and anti-mouse IgG Alexa 488 antibody (Molecular Probes, OR, USA) was used as the antibody.

Cell migration assay and wound healing assay

We examined the migration of lung cancer cells in the presence Ctrl Nb or A5 Nb with the use of 8-µm pores transwell inserts (Corning Inc, MA, USA). After treatment with either Ctrl Nb or A5 Nb (10 µg/mL), lung cancer cells (1.0 × 10^5^ cells in RPMI serum-free medium with Nbs (10 µg/mL) were seeded into the upper chamber of the transwell. The insert was then placed in a 24-well plate containing RPMI medium with 10% FBS in the lower chamber as a chemoattractant. After cells were allowed to migrate for 24 h in a humidified chamber, those that had migrated were stained with a 0.5% crystal violet (w:v) in 20% methanol and counted by light microscopy in five random fields (×200 original magnification) per sample. The experiments were performed in duplicate and repeated thrice.

For the wound healing assay, the cells were cultured in SPL Scar Block (SPL Life Sciences, Seongnam, Republic of Korea). Scar Block is composed of 500 μm-thick walls to generate cell free gaps. When the cells were confluent, the block was removed from the plate and culture medium was added. Cellular migration was monitored and photographed at 0, 24 h, and 40 h with nanobodies (10 µg/mL).

Matrigel invasion assay

The Matrigel invasion assay was performed in a chamber system consisting of polycarbonate membrane inserts with 8-µm pores (Corning Inc.) placed in 24-well cell culture insert companion plates. The inserts were coated with a thin layer of 0.5 mg/mL Matrigel Basement Membrane Matrix (BD Biosciences). An invasion assay was conducted 48 h after the Nbs were added to the cells. The cells (1 × 10^4^ cells in 200 µL of growth medium without FBS) were placed in the upper chamber, and 0.5 mL of growth medium containing 10% FBS was placed in the lower chamber. The cells were incubated at 37 °C and allowed to invade through the Matrigel layer for 48 h. After incubation, the insert membranes were fixed with 4% paraformaldehyde for 15 min. The cells on the upper surface were removed using cotton-tipped swabs, and the invading cells on the lower surface were stained with 0.5% crystal violet (w:v) in 20% methanol for 60 min. Stained cells were counted under an inverted microscope (five fields per membrane). The experiments were performed in duplicate and repeated thrice.

Immunohistochemical staining

Cells were washed with PBS (pH 7.4, 37 °C), fixed with 4% paraformaldehyde for 15 min at 37 °C, and then permeabilized with 0.1% Triton X-100 (v/v) for 15 min at 4 °C. After fixation, the cells were washed twice with PBS and incubated for 30 min at 25 °C with 2% (w/v) BSA solution. Primary antibodies were added and incubated overnight at 4 °C. After incubation, the cells were washed thrice with PBS for 10 min, followed by incubation with secondary antibodies. After 1 h of incubation at RT, the cells were washed thrice with PBS for 5 min. The cells were mounted with Vectashield mounting medium (Fluoroshield, Abcam, MA, USA), and fluorescence images were acquired using a confocal microscope. PXN (1:200) primary antibody and anti-rabbit IgG Alexa 594 secondary antibody (1:500, Molecular Probes) were used.

Distribution of A5-LNP

Five-week-old female BALB/c nude mice (Japan SLC Inc., Hamamatsu, Japan) were subcutaneously injected with 5 × 10^6^ A549 cells. Mice were randomly divided into groups after the tumor volume reached 400 mm^3^. Five tumor-bearing mice in each group were imaged prior to intravenous injection of ICG-labeled LNP (LNP-ICG)^7^ and ICG-labeled A5-LNP (A5-LNP-ICG) at a dosage of 8 mg/kg, and images were captured at 24 h and 48 h after injection. Images of the three mice were captured using the IVIS-200 system (PerkinElmer, MA, USA).

**Bioluminescence Imaging**

Bioluminescence imaging was performed using an IVIS Lumina in vivo imaging system (Caliper LifeSciences, MA, USA). NSG mice were anesthetized by 2% isoflurane inhalation followed by orthotopic injection of D-luciferin (150 mg/kg) *via* intraperitoneal (i.p.) injection. Bioluminescent images were acquired 15 min after luciferin injection.^8^

**Lung cancer organoid culture**

The Organoids were incubated in a 37 ℃ and 5% CO_2_ culture incubator. The culture medium consisted of 40% W/V basal culture medium (advanced Dulbecco’s Modified Eagle Medium/Ham’s F-12; GIBCO, Cat#P75850694), 50% W/V L-WRN conditioned medium(Sigma-Aldrich, Cat# SCM105), 1 × GlutaMAX supplement (GIBCO, Cat# 35050061), HEPES (10 mM) (GIBCO, Cat# 15630056) 1 × B27 (GIBCO, Cat# 17504-044), human EGF (50 ng/mL) (Pepprotech, Cat# AF-100-15), nicotinamide (10 mM) (Sigma-Aldrich, Cat# N3376), N-acetylcysteine (1.25 mM) (Sigma-Aldrich, Cat# A9165), A83-01 (500 nM) (Sigma-Aldrich, Cat# SML0788), and 1 × Antibiotic-Antimycotic (GIBCO, Cat# 15240062).

Study of anti-tumor efficacy

Female BALB/c nude mice (Japan SLC Inc.), approximately 6–8 weeks old, were subcutaneously inoculated with 5 × 10^6^ A549 cells. When the tumor volume reached ~100 mm^3^, mice were randomly divided into groups. Each group (n > 5) was administered 5 mg/kg of A5 Nb, LNP-DOX or A5-LNP-DOX (A5 concentration 100 μg/mL) by tail vein every 2 d for 16 d, and the control group was administered the same amount of PBS or Ctrl Nb. Body weight and tumor diameter were measured at specific time intervals. Blood and major organs were collected 16 d after drug administration. Serum levels of urea nitrogen glutamic oxaloacetic transaminase (GOT; AST), and glutamic pyruvate transaminase (GPT; ALT) were analyzed. Organs (liver, heart, lungs, spleen, and kidneys) were sectioned and stained with hematoxylin and eosin (H&E) for histological examination.

Immunohistochemical staining

Frozen tumor sections were air-dried and fixed in cold acetone for 10 min. Sections were blocked with 2.5% goat serum in PBS for 30 min. The sections were then incubated with an anti-Ki67 antibody (1:200; Thermo Fisher Scientific), or anti-CD31 antibody (1:200; Thermo Fisher Scientific) overnight at 4°C. After washing with PBS, the sections were incubated with anti-mouse IgG Alexa Fluor 488 and anti-rabbit IgG Alexa Fluor 594 secondary antibodies (1:500; Molecular Probes).

For statistical analyses, sections from five randomly selected tumors per group were stained, and five random fields per tumor were scored. Images were taken at ×200 or ×100 magnification.

Human lung cancer tissue array (#LC811a) was purchased from US Biomax Inc. (Derwood, MD, USA), tissue array slides were deparaffinized, antigen-retrieved and permeabilized as already described and stained with anti-CD155 (1:100; Thermo Fisher Scientific), and PXN (1:200; Cell signaling technology, Danvers, MA, USA).

**Quanlitative analysis of CD155 expression**

For analysis of CD155 isoforms, amplifications were performed with 2 μl cDNA in a total volume of 50 μl PCR mixture with primers CD5 and CD15 (Sequences in Supplementary table 4). PCR amplifications were carried out for 35 cycles (30 s at 92 °C; 30 s at 60 °C; and 1 min at 72 °C) followed by a 3 min final extension at 72 °C. Amplified fragments were visualized after electrophoresis on 10% polyacrylamide gel electrophoresis with 1 × SYBR Green I Nucleic Acid Gel Stain (Thermo Fisher Scientific). ^9^


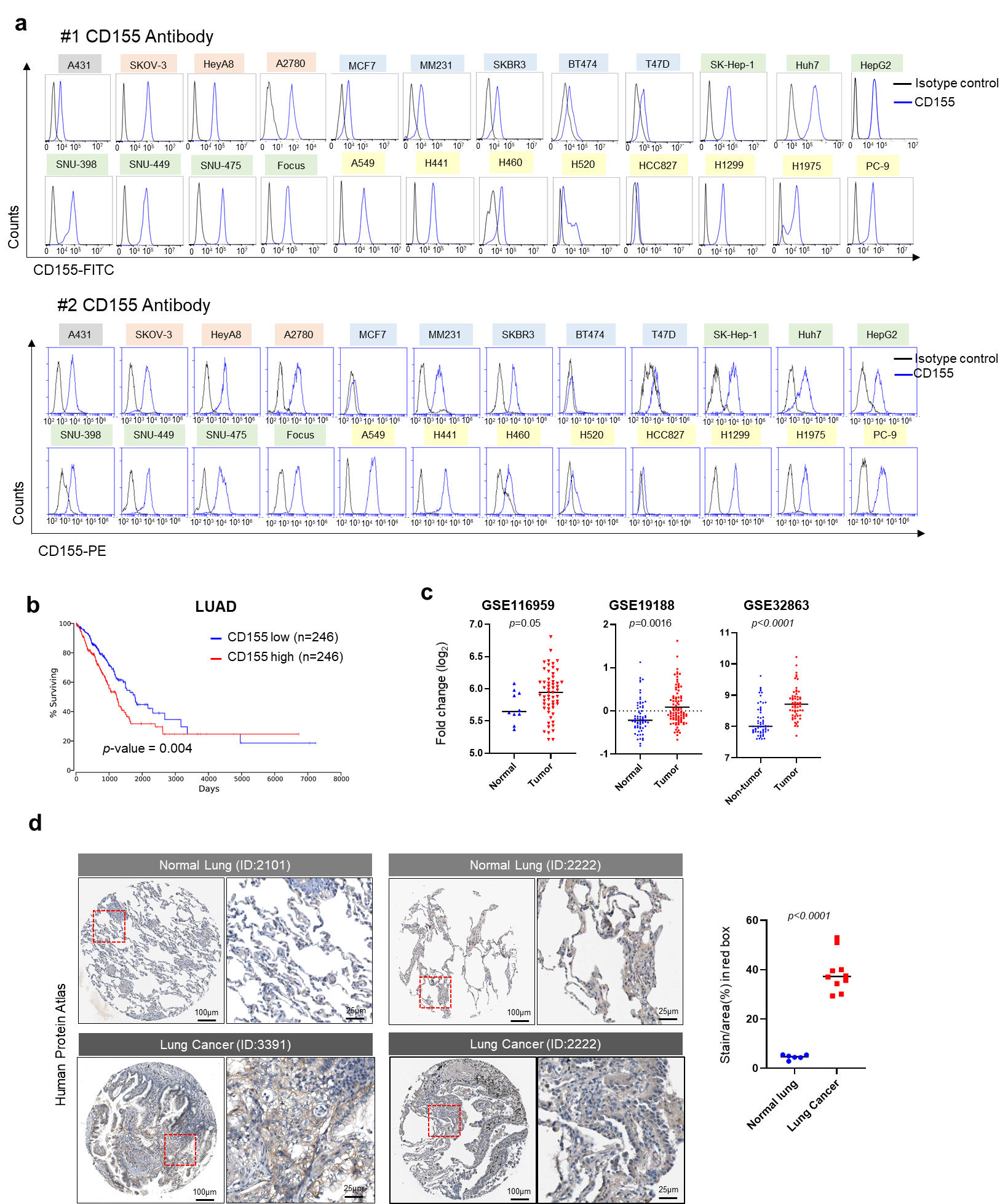


**Supplementary Figure 1.** Validation of CD155 expression in public database

**a.** The expression level of CD155 was measured by flow cytometry with two different CD155 antibodies (#1 CD155 antibody; Invitrogen, Cat#MA5-13493, #2 CD155 antibody; BioLegend, Cat# 337507) in various cancer cell lines. Mouse IgG1 was used as an isotype control. Epidermoid carcinoma (A431), ovarian cancer (SKOV-3, HeyA8, and A2780), breast cancer (MCF7, MDA-MB-231, SKBR3, BT474, and T47D), hepatocellular carcinoma (SK-Hep-1, Huh7, HepG2, SNU398, SNU-449, SNU-475, and Focus), lung cancer (A549, H441, H460, H520, H1299, HCC827, H1299, H1975, and PC-9) **b.** Kaplan-Meier survival analysis of lung cancer patients from The Cancer Genome Atlas (TCGA) according to CD155 expression levels (*p* = 0.004). **c.** The expression of CD155 according to GEO database. GSE116959 (Normal lung tissue; n = 10, lung cancer tissue; n = 57, *p* = 0.05), GSE19188 (Normal lung tissue; n = 65, lung cancer tissue; n = 91, *p* = 0.0016), GSE32863 (Normal lung tissue; n = 58, lung cancer tissue; n = 58, *p* < 0.0001) **d**. Representative immunohistochemistry images of CD155 in normal lung tissue and lung cancer tissue (Human Protein Atlas). The scale bar represents 100 μm and 25 μm. The percentage of DAB-stained area was calculated from three randomly selected regions per sample. *p*-value determined by two-tailed Student’s t-test.


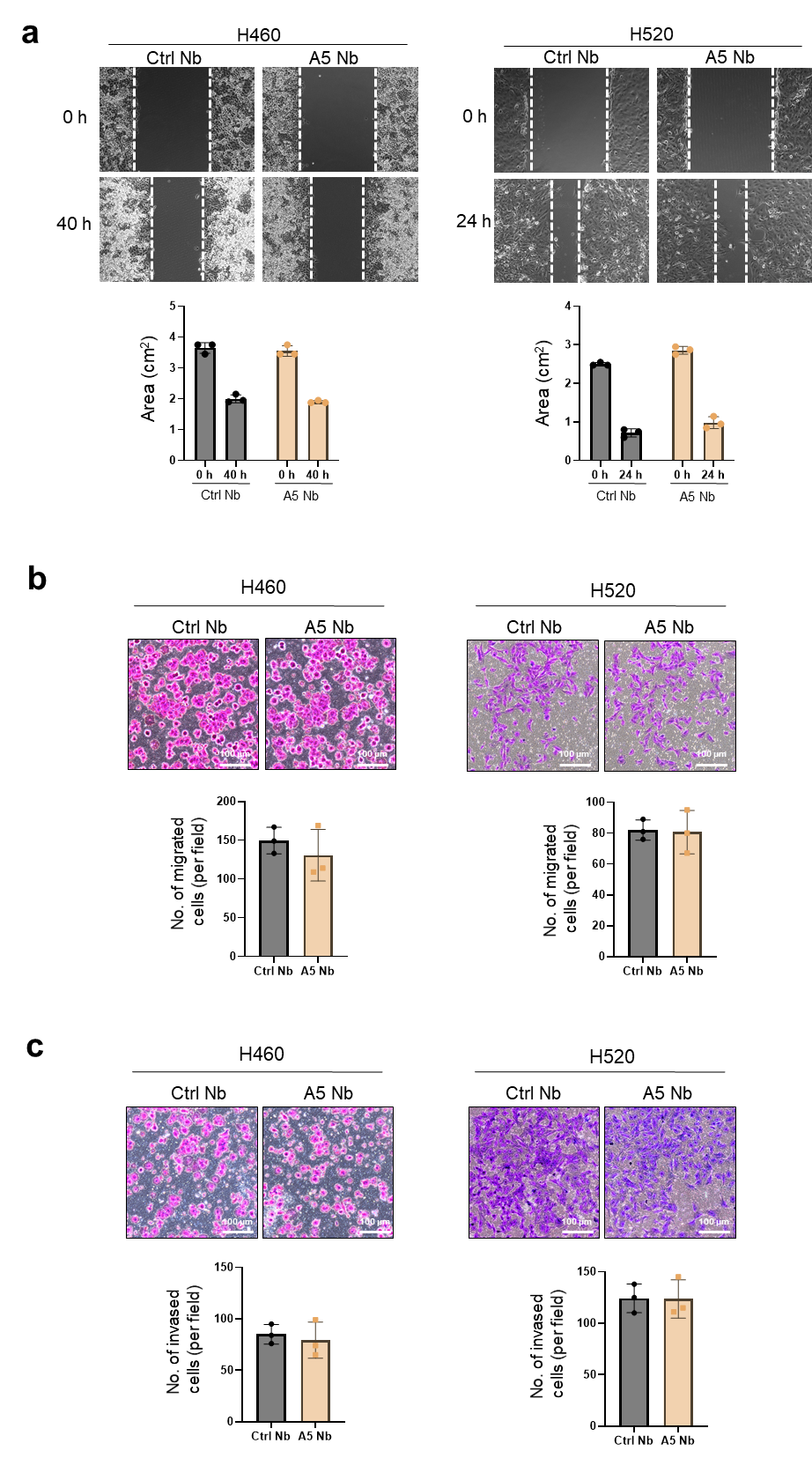


**Supplementary Figure 2.** No Effect of CD155 nanobody on CD155-low expressed lung cancer cell migration and invasion.

**a**. Representative images of scar and recovering of wounded areas (marked by white lines) on confluence monolayers of H460 and H520 cells at 0, 40 h or 0, 24 h with A5 Nb (10 μg/mL) or Ctrl Nb (10 μg/mL) treatment and evaluation of scar wound healing in %. **b**. Transwell migration assay. H460 and H520 cells were seeded on transwell insert and incubated with A5 Nb (10 μg/mL) or Ctrl Nb (10 μg/mL) for 24 h. **c**. For the invasion assay, the membrane was pre-coated with 50 μL Matrigel. H460 and H520 cells were seeded on matrigel and incubated with A5 Nb (10 μg/mL) or Ctrl Nb (10 μg/mL) for 36 h. Scale bar = 100 μm Data represented as mean values ± SD, determined by two-tailed Student’s t-test.

**
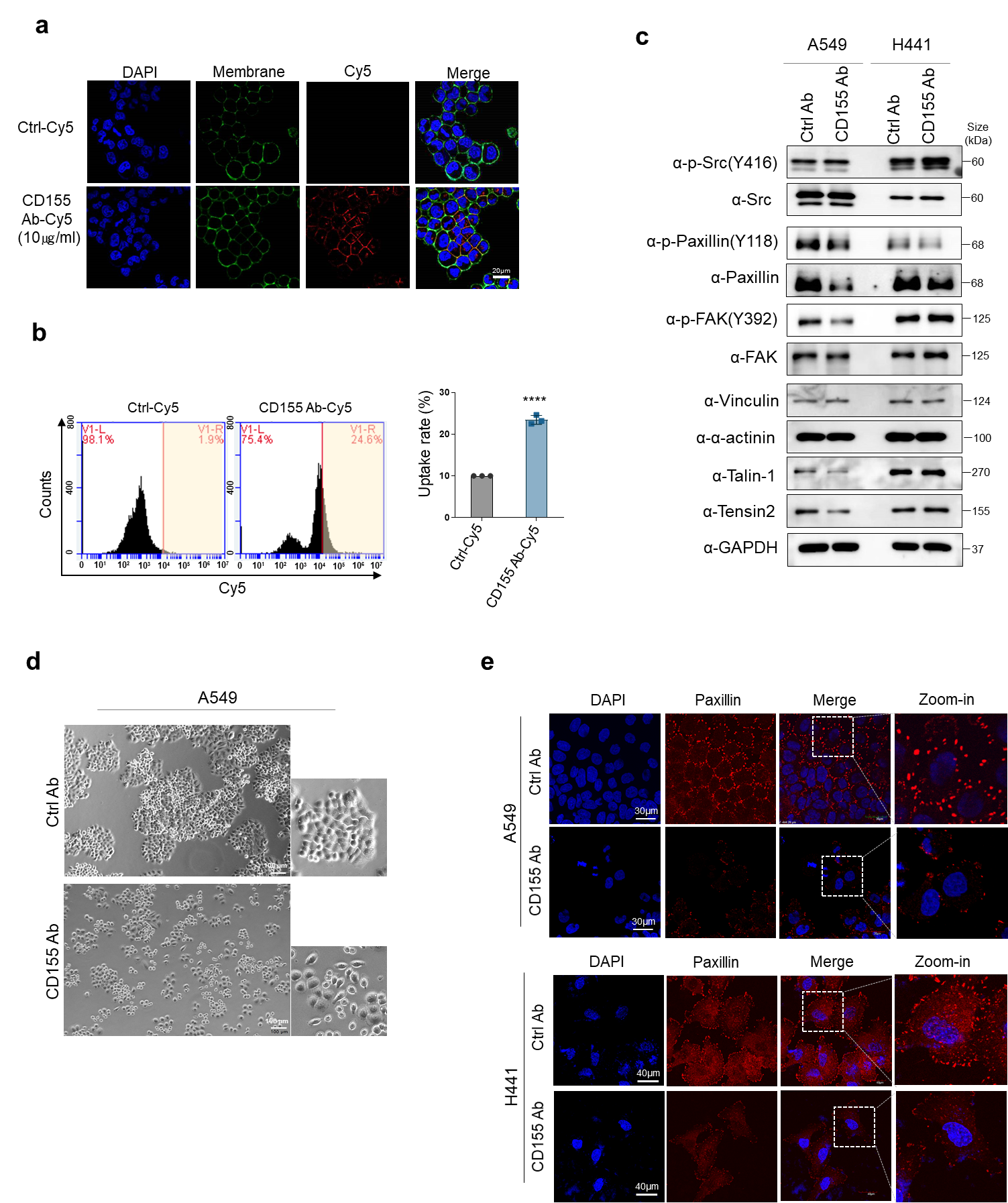
**

**Supplementary Figure 3.** Internalization of CD155-targeted CD155 antibody overexpressed cancer cells

**a.** Cellular internalization images of fluorescently labeled nanobodies obtained by confocal laser scanning microscopy. CD155 Ab conjugated with Cy5 and treated in A549 cells; scale bar indicated 20 μm. **b.** % of cellular uptake was analyzed using flow cytometry with Ctrl-Cy5 and CD155 Ab-Cy5. Data represented as mean values ± SD, determined by two-tailed Student’s t-test *: *vs.* Ctrl-Cy5, *****p* < 0.0001. Concentration of CD155 Ab is 10 μg/mL. **c.** pSrc/Src, pPaxillin/Paxillin, and pFAK/FAK pathway and focal adhesion complex proteins (Vinculin, α-actinin, Talin-1, and Tensin2) was measured in A549 and H441 cells after CD155 Ab (100 μg/mL) treatment *versus* Ctrl Ab. **d.** Cellular morphology of A549 and H441 after CD155 Ab (100 μg/mL) treatment observed under an inverted light microscope. **e.** Representative images show expression of paxillin after treatment of CD155 Ab (100 μg/mL) in A549 and H441 cells compared to Ctrl Ab for 24 h. Nuclei were visualized with DAPI staining. Scale bar = 30 μm (A549) and 40 μm (H441).


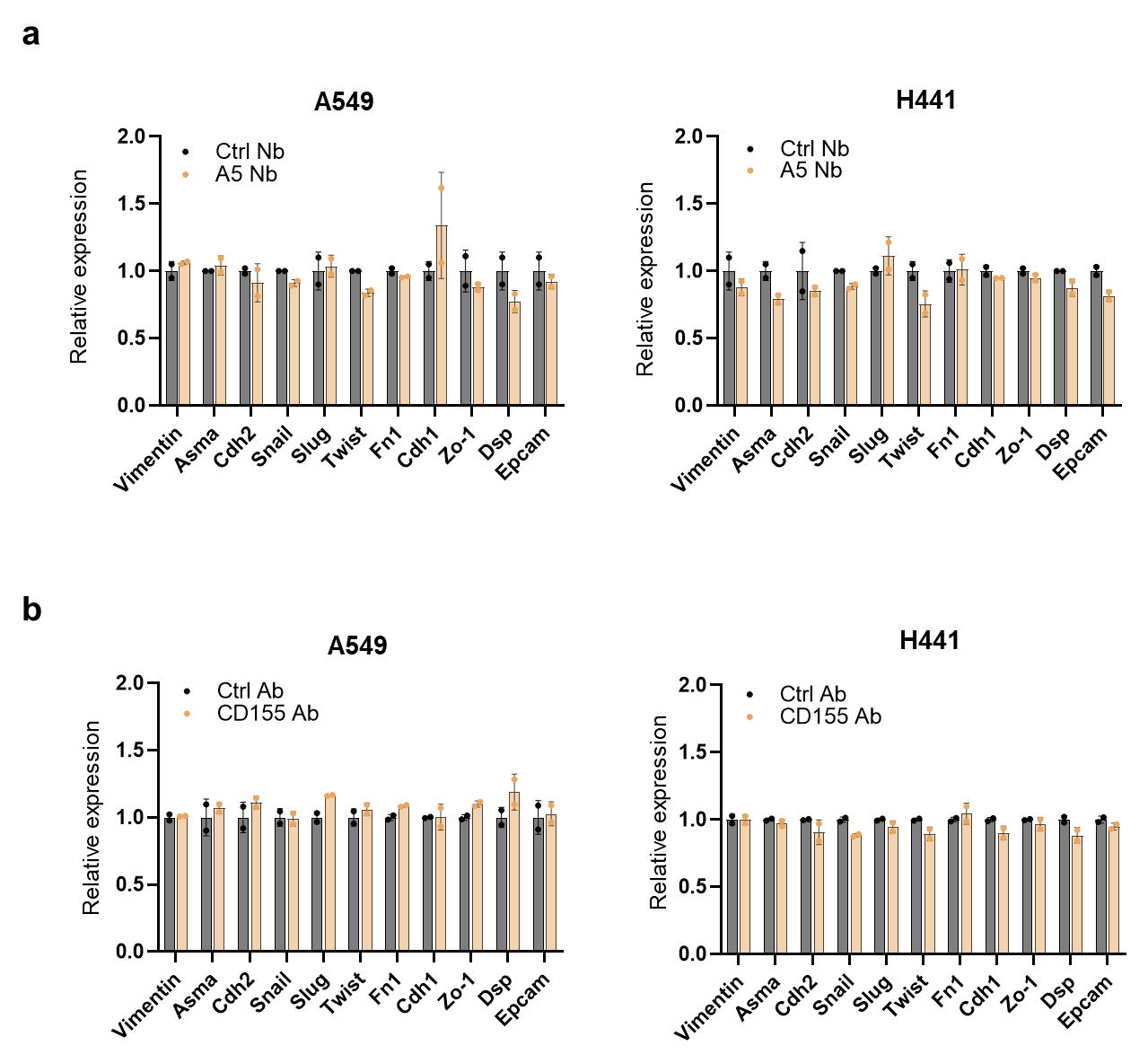


**Supplementary Figure 4.** CD155 blocking of lung cancer cells results in no change of EMT related genes.

qPCR results of EMT-related gene mRNA expression in A549 and H441 cells treated with A5 Nb compared to Ctrl Nb (a) and CD155 Ab compared to Ctrl Ab (b) for 24 h. Nb concentration is 10 μg/ml and Ab concentration is 100 μg/ml. Data represented as mean values ± SD, determined by two-tailed Student’s t-test.


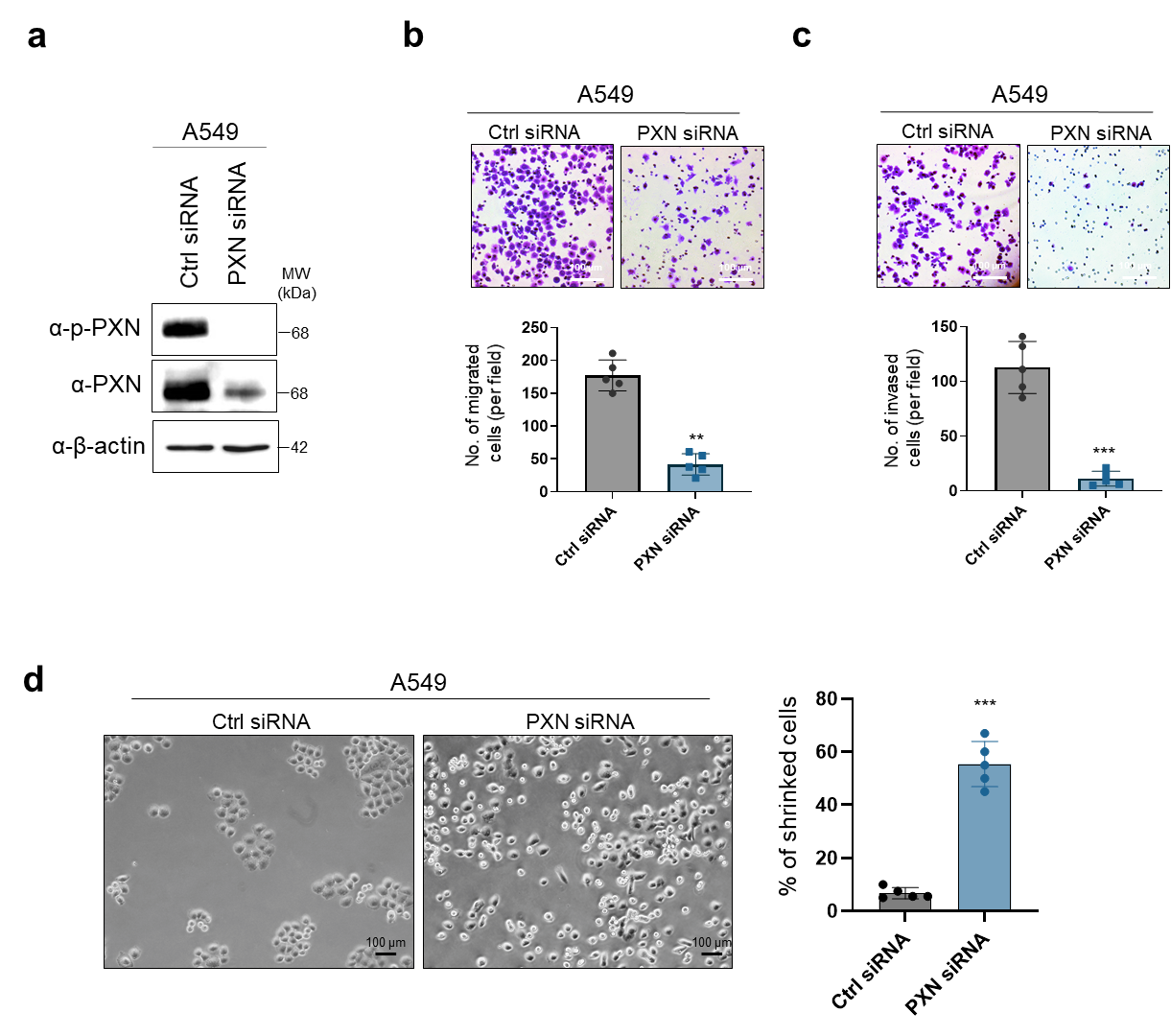


**Supplementary Figure 5.** Silenced of PXN regulated cell migration and invasion in lung cancer.

**a**. PXN expression in A549 cells after silencing of PXN was assessed by Western blotting in siPXN- or siCtrl-transfected A549 cells. Migration (**b**) and invasion (**c**) ability assessed by transwell assay. **d.** Cellular morphology of A549 after silencing of PXN observed under an inverted light microscope. Quantification of shrinked cells was performed using ImageJ. Scale bar = 100 μm. Data represent means ± SD, determined by two-tailed Student’s t-test *: *vs.* Ctrl. siRNA, ***p* < 0.01, and ****p* < 0.001.


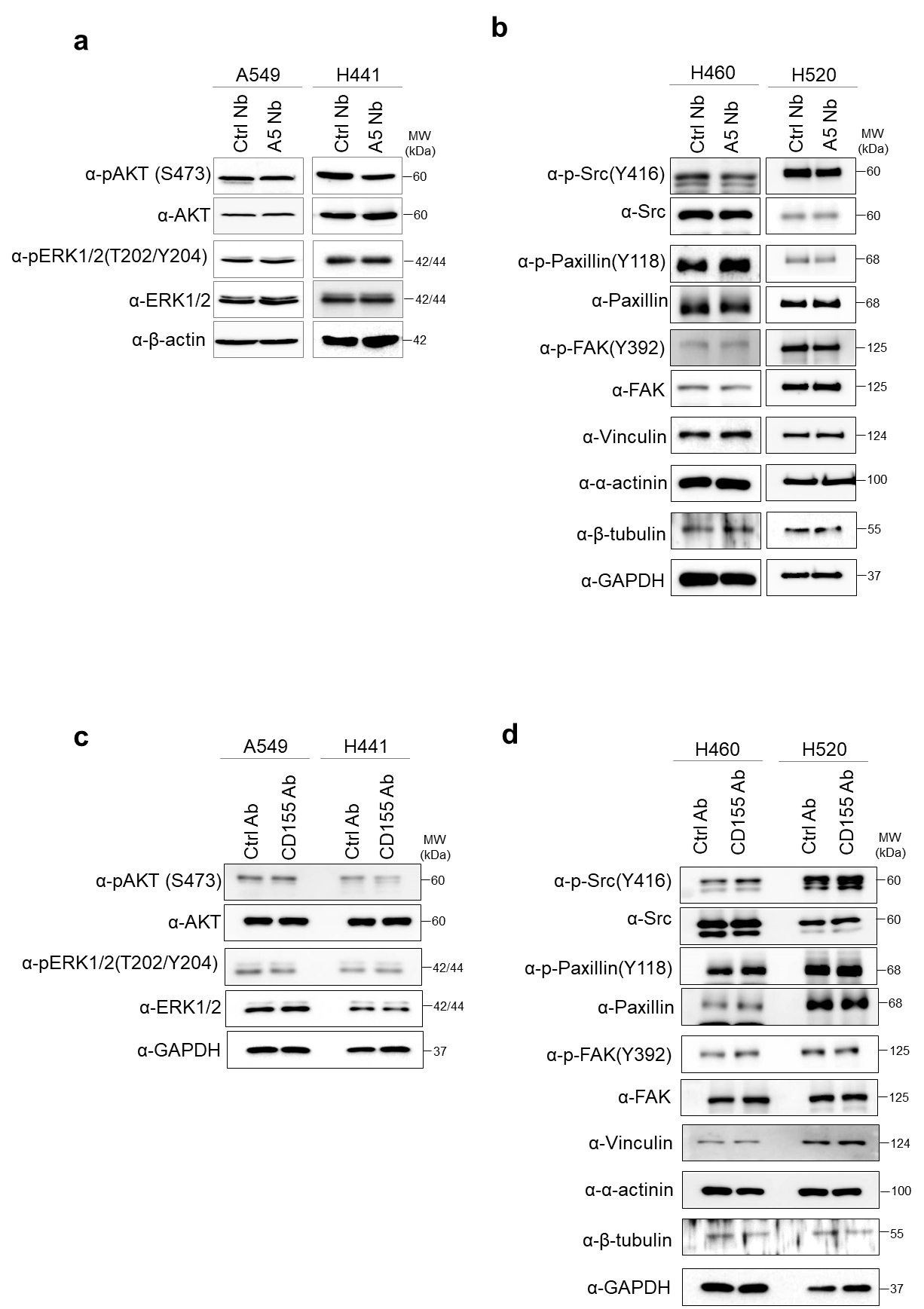


**Supplementary Figure 6.** CD155 blocking results in no change of AKT/ERK signaling and CD155 low expressed lung cancer no effect of Src/PXN/FAK signaling despite A5 Nb treatment.

**a** and **c**. AKT/ERK pathway was measured by Western blotting in A5 Nb treated A549 and H441 cells compared to Ctrl Nb treated (**a**) and CD155 Ab treated condition (**c**). **b** and **d**. pSrc/Src, pPaxillin/Paxillin, pFAK/FAK pathway and focal adhesion complex proteins (Vinculin, α-actinin, and β-tubulin) was measured in H460 and H520 cells after A5 Nb treatment *vs.* Ctrl Nb (**b**) and CD155 Ab treated condition (**d**). Nbs concentration are 10 μg/ml and Abs contrantion are 100 μg/ml.


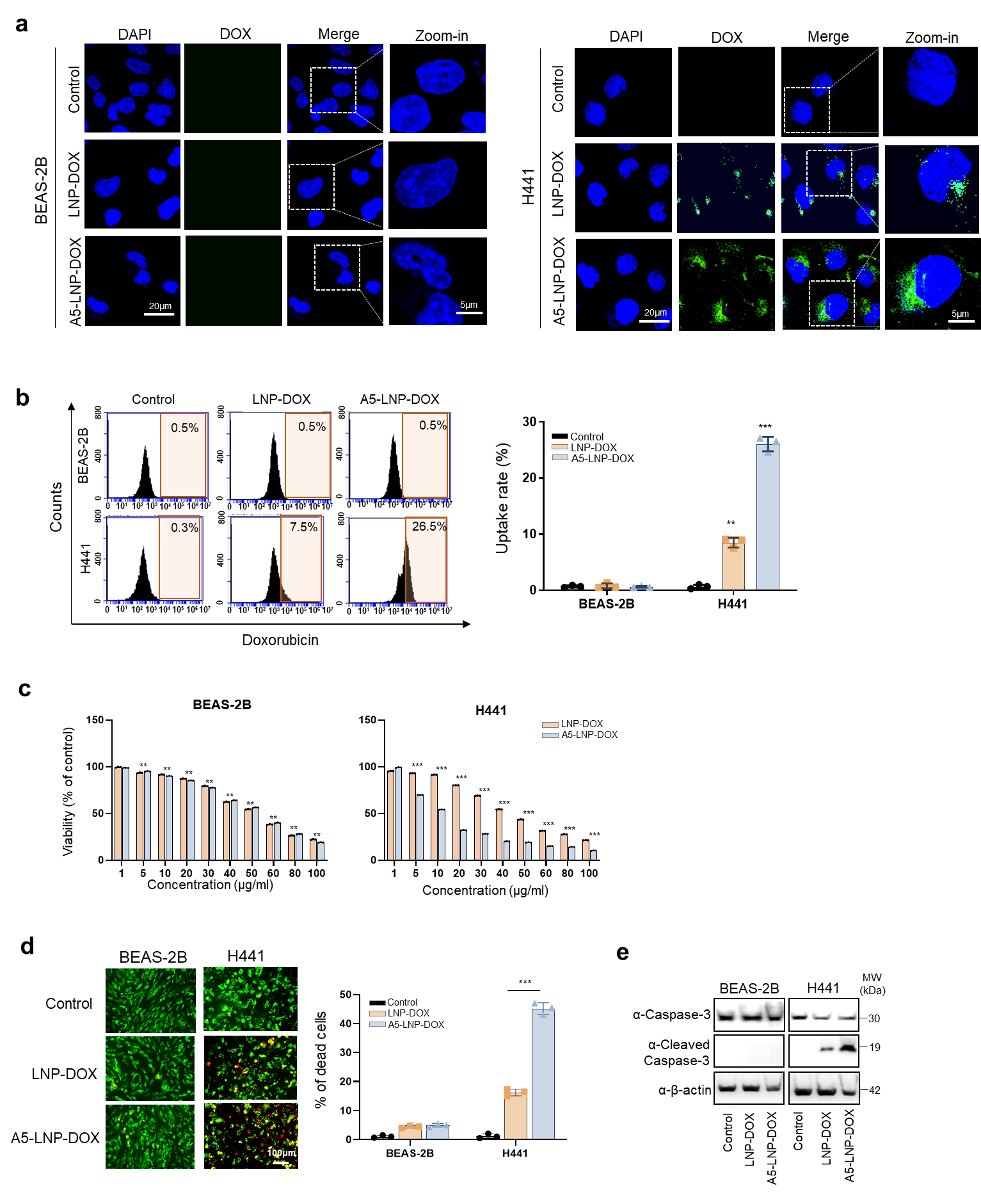


**Supplementary Figure 7.** A5-LNP-DOX inhibited the viability of CD155-high expressing H441 cells, but did not significantly affect CD155-negative BEAS-2B cells.

**a.** Confocal imaging was performed to analyze the intracellular regions of liposomes. BEAS-2B and H441 cells were exposed to LNP-DOX and A5-LNP-DOX (green) for 4 h. The scale bars indicated 20 μm and 5 μm (zoom-in). **b.** Cellular uptakes were evaluated by flow cytometry. All LNP-DOX concentration is 200 μg/mL **c.** *In vitro* cytotoxicity was assessed on H441 and BEAS-2B cells for LNP-DOX or A5-LNP-DOX. Cells were treated with LNPs at various concentrations for 8 h, after which the medium was replaced and cells were further incubated for 24 h. The LNPs concentration varied at 1, 5, 10, 20, 30, 40, 50, 60, 80, and 100 μg/mL. Data represent mean ± SD. (n=3) *: *vs.* LNP-DOX, ***p* < 0.01, ****p* < 0.001. **d**. H441 and BEAS-2B cells were treated with LNP-DOX and A5-LNP-DOX for 8 h and then the medium was replaced and cells were cultured for an additional 24 h followed by live/dead staining. The cells were stained with calcein and propidium iodide resulting in the appearance of live cells in green and dead cells in red. Scale bar = 100 μm **e**. Detection cleaved caspase-3 in apoptotic cells by western blots. Data represented as mean values ± SD, determined by two-tailed Student’s t-test *: *vs.* Control, ***p* < 0.01, and ****p* < 0.001.


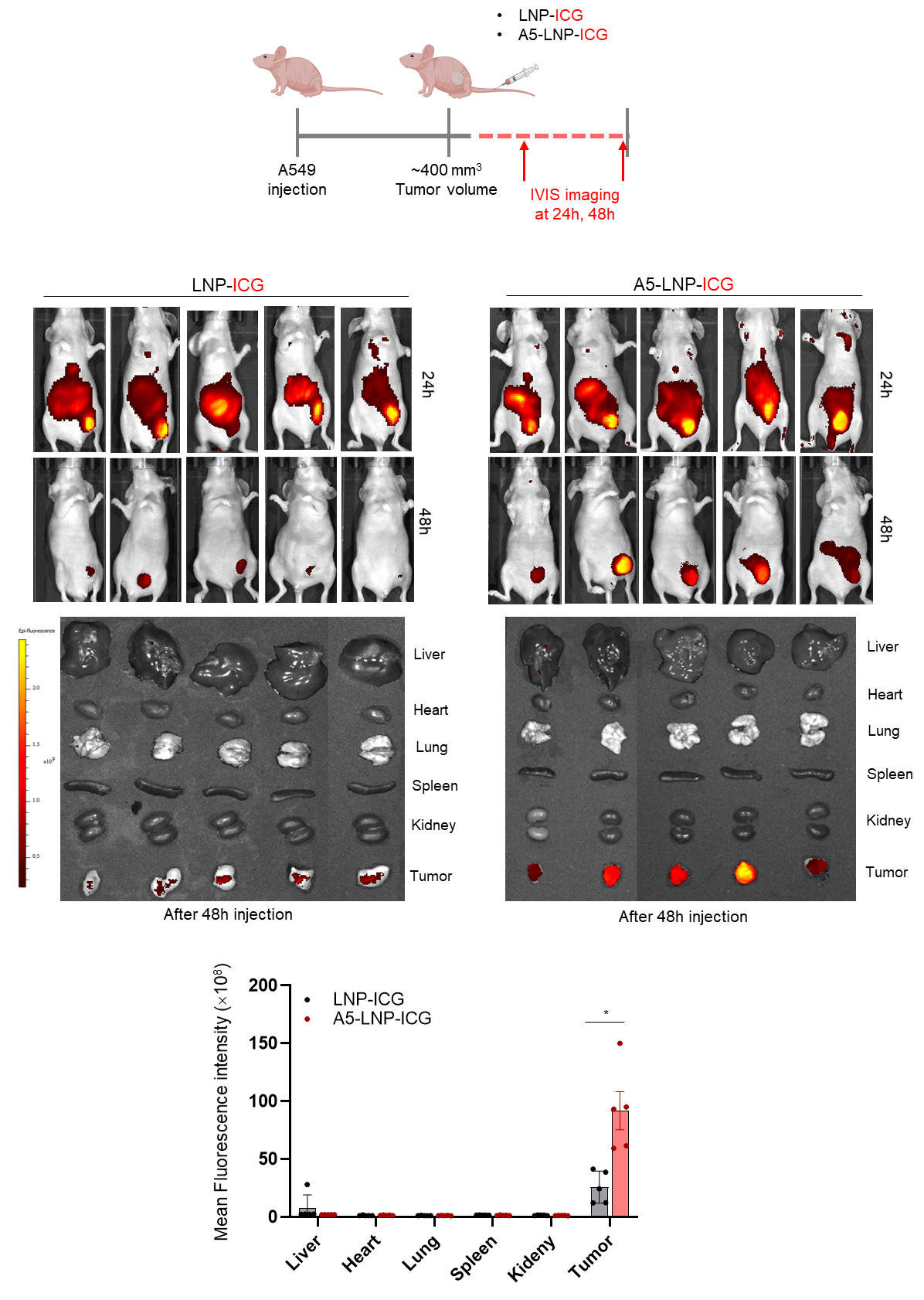


**Supplementary Figure 8.** *In vivo* biodistribution of LNP-ICG and A5-LNP-ICG.

After subcutaneous transplantation of tumor cells, A549 tumor bearing nude mice were intravenously administered the LNPs. After subcutaneous transplantation of A549 tumor cells, xenograft nude mice (n = 5 per group) were intravenously administered LNP-ICG or A5-LNP-ICG. Whole-body fluorescence imaging was conducted at 24 and 48 h post-injection to assess *in vivo* biodistribution and tumor accumulation. At 48 h, mice were sacrificed and major organs (liver, heart, lungs, spleen, kidneys) and tumors were harvested for *ex vivo* imaging. Fluorescence intensity in each organ and tumor was quantified and is presented in the bar graph as mean ± SEM. Statistical analysis was performed using two-tailed unpaired Student’s t-test. *: *vs.* LNP-ICG, **p* < 0.05.


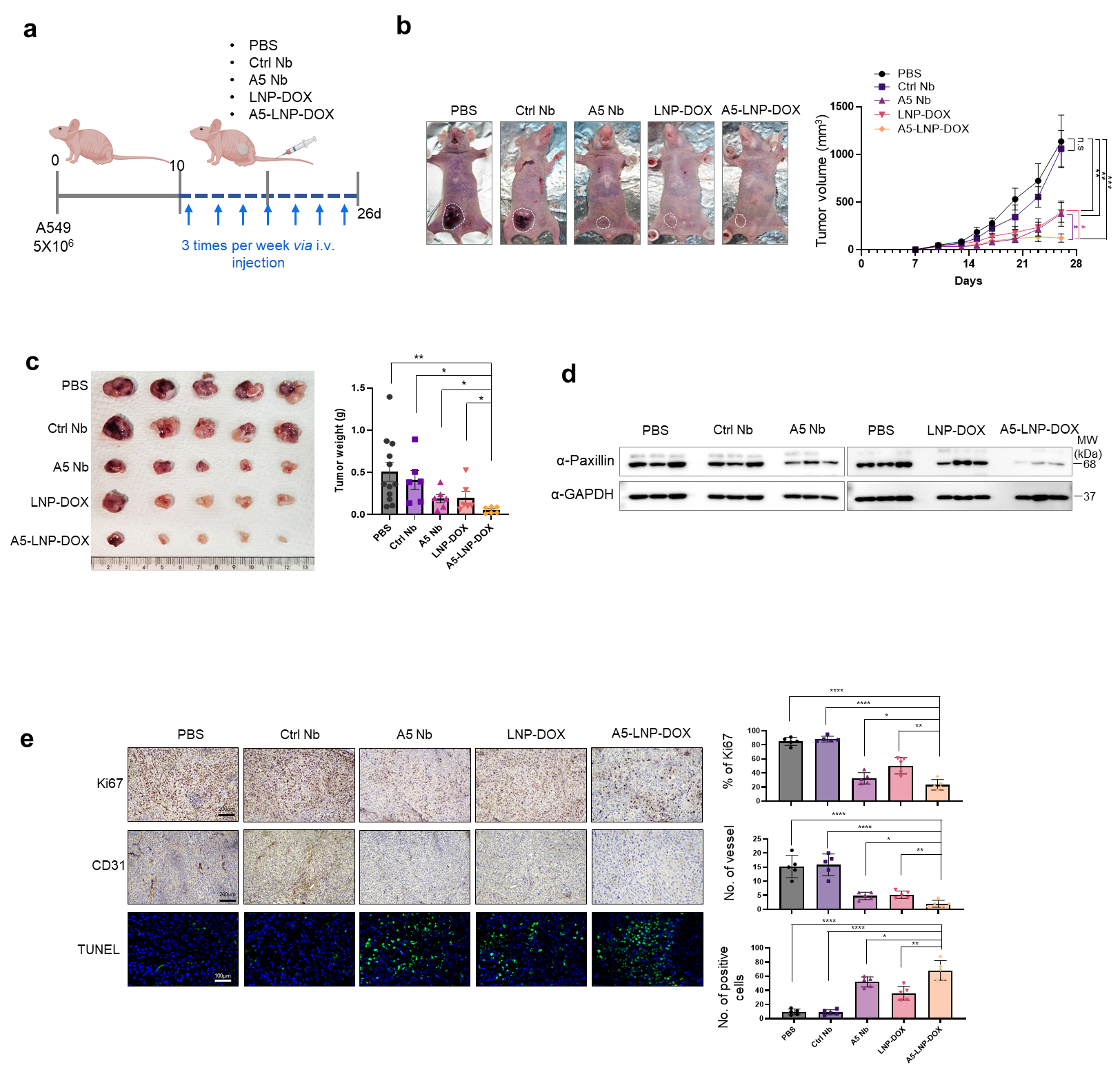


**Supplementary Figure 9.** *In vivo* antitumor efficacy of LNP-DOX and A5-LNP-DOX.

**a.** An antitumor effect *in vivo* was observed for PBS, Ctrl Nb (5 mg/kg), A5 Nb (5 mg/kg), LNP-DOX (4 mg/kg), and A5-LNP-DOX (4 mg/kg). **b.** Tumor volume was measured after injection (3 times per week) of the tumor cells, and the growth curves of the tumors were recorded. **c.** Images of tumors harvested at the endpoint and tumor weights. Data were expressed as mean ± SEM. The sample size was n = 12 for the PBS group and n = 6 for all other groups. *: *vs.* PBS, **p* < 0.05, ***p* < 0.01, ****p* < 0.001, and *****p* < 0.0001, ^#^: *vs.* A5-LNP-DOX, ^#^*p* < 0.05. **d.** Paxillin expression. **e.** Ki67 IHC (**e top**) and CD31 IHC (**e middle**) staining of tumors from PBS-, Ctrl Nb-, A5 Nb-, LNP-DOX-, and A5-LNP-DOX-treated group. Detection of apoptosis by TUNEL assay (**e bottom**) in tumor tissue. For statistical analysis, five randomly selected tumors per group were stained and five random fields per tumor were scored. Scale bar = 200 μm (Ki67 and CD31) and 100 μm (TUNEL) for **e**. a and b mouse icon created by BioRender. Data represented as mean values ± SD, determined by two-tailed Student’s t-test for B and E. *: *vs.* A5-LNP-DOX, **p* < 0.05, ***p* < 0.01, ****p* < 0.001, and *****p* < 0.0001.


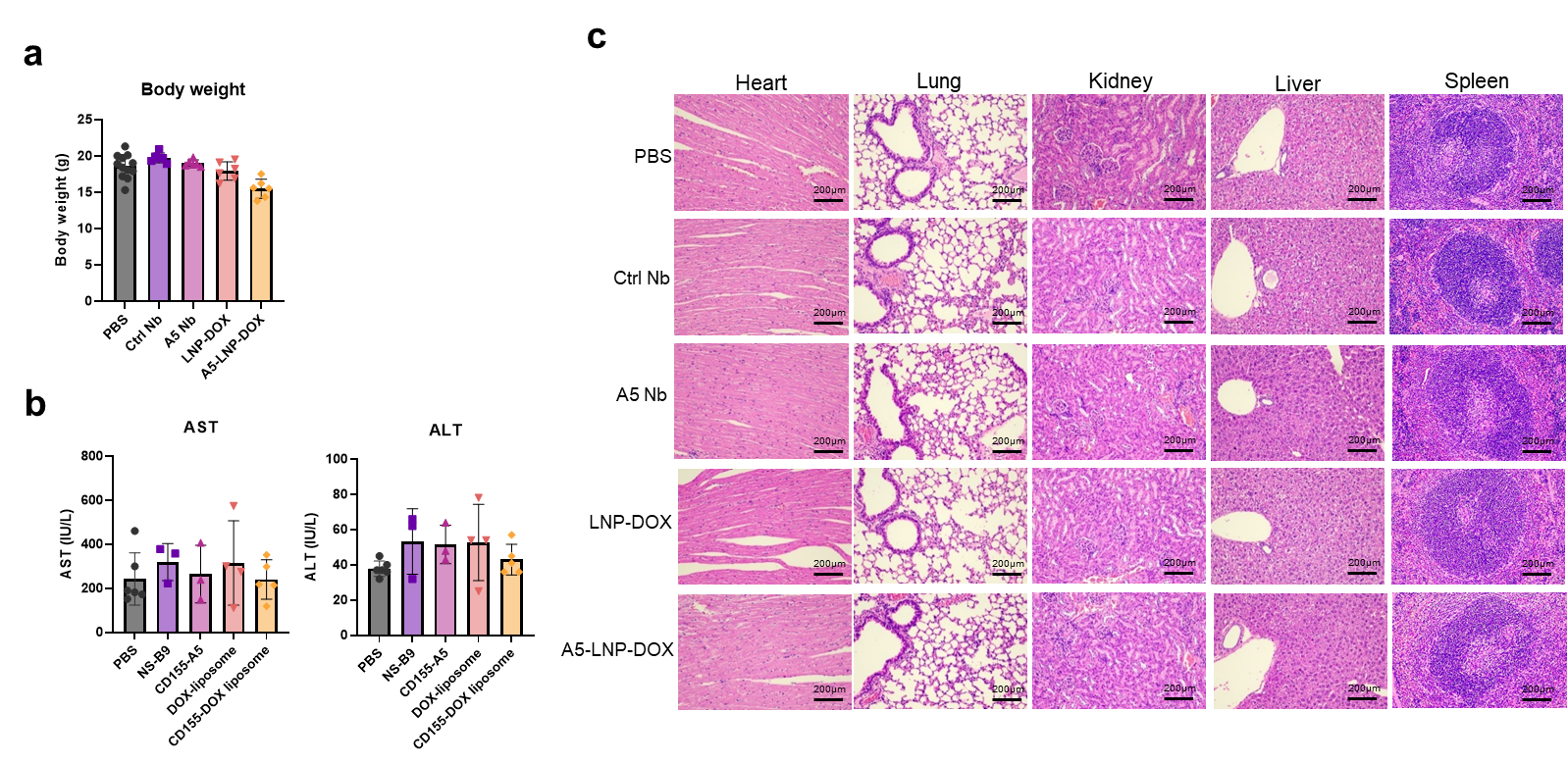


**Supplementary Figure 10.** *In vivo* toxicity evaluation of antitumor reagents

**a.** Quantification of body weight after treatment with PBS, Ctrl Nb, A5 Nb, LNP-DOX, and A5-LNP-DOX. **b.** Measurement of AST and ALT in the serum of each group of mice (n = 5 per group). **c.** H&E staining of the heart, lungs, kidneys, liver and spleen tissues. Images were captured at ×200 magnification. The scale bar represents 200 μm.


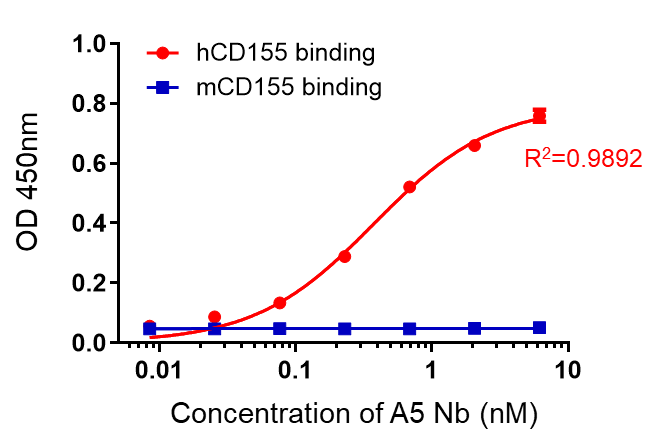


**Supplementary Figure 11.** The specificity of A5 Nb to human CD155 and mouse CD155 antigen was detected by ELISA. The above experiments were performed in triplicate and data were presented as mean ±SD. R² value represents the binding curve fit between human CD155 and A5 Nb.


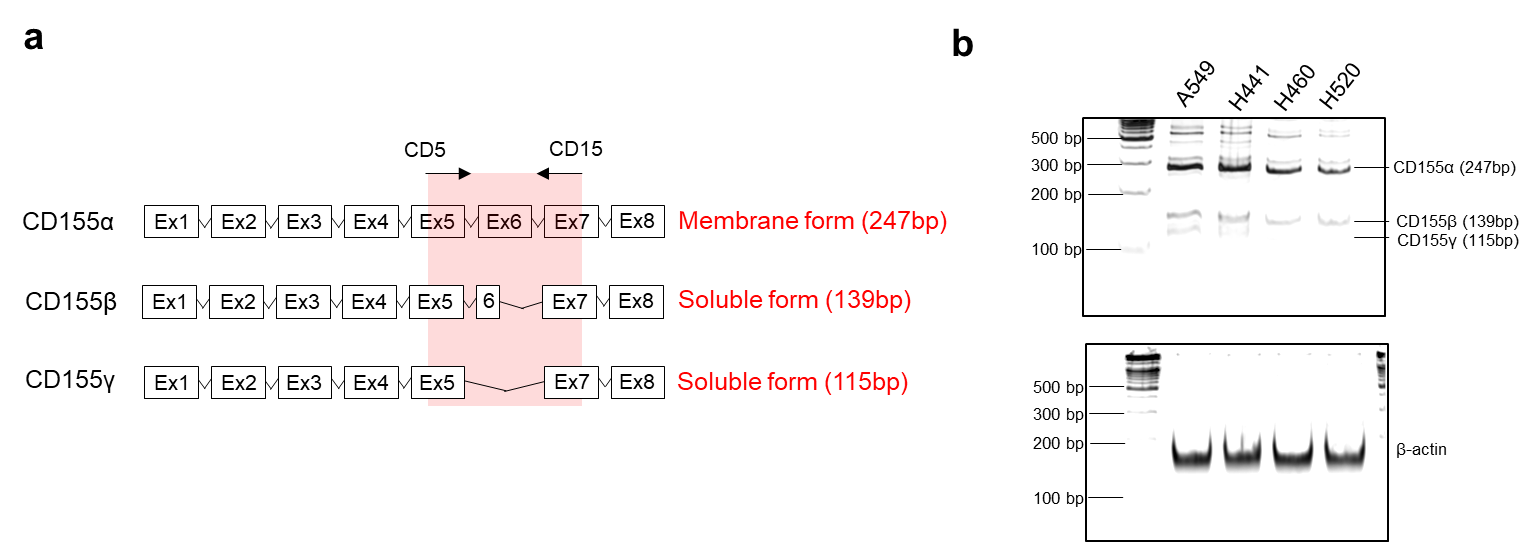


**Supplementary Figure 12.** **a**. Schematic representation of the different CD155 transcript variants. Oligonucleotide primers CD5 and CD15, used for qualitative analysis of CD155 expression, are located on each side of the different splice sites. The products size are 247 bp (CD155α), 139 bp (CD155β), and 115 bp (CD155γ). **b**. CD155α, CD155β, and CD155γ transcripts showing in A549, H441, H460, and H520 cell lines**.** β-actin transcripts is loading control.

Supplementary Table1.

| **Cancer** | **COX Coefficient** | **P-Value** | **FDR Corrected** | **Rank** | **Median Expression** |
| --- | --- | --- | --- | --- | --- |
| LUAD | 0.263 | 4.40E-04 | 1.81E-02 | 409 | 1192.33 |
| KIRP | 0.576 | 1.50E-03 | 1.33E-02 | 1844 | 1240.44 |
| BLCA | 0.194 | 1.10E-02 | 1.08E-01 | 1644 | 1088.59 |
| CESC | 0.363 | 1.10E-02 | 1.81E-01 | 983 | 1062.15 |
| HNSC | 0.177 | 1.20E-02 | 1.87E-01 | 1052 | 1309.96 |
| LGG | 0.198 | 3.80E-02 | 7.41E-02 | 8616 | 381.96 |
| SKCM | 0.121 | 7.60E-02 | 2.21E-01 | 5522 | 992.04 |
| KIRC | 0.121 | 1.30E-01 | 2.28E-01 | 9458 | 946.92 |
| LUSC | 0.102 | 1.30E-01 | 6.44E-01 | 3365 | 1010.7 |
| BRCA | 0.14 | 1.40E-01 | 5.09E-01 | 4507 | 509.52 |
| SARC | 0.14 | 1.80E-01 | 4.60E-01 | 6297 | 899.06 |
| STAD | 0.105 | 2.20E-01 | 5.99E-01 | 6172 | 1609.49 |
| ESCA | 0.17 | 2.20E-01 | 9.72E-01 | 3762 | 1550.85 |
| LAML | 0.135 | 2.30E-01 | 6.23E-01 | 5572 | 590.39 |
| PAAD | -0.116 | 2.90E-01 | 5.04E-01 | 9866 | 1467.06 |
| COAD | 0.099 | 3.50E-01 | 7.29E-01 | 7839 | 1704.31 |
| UCEC | -0.084 | 4.00E-01 | 9.93E-01 | 6684 | 1016.49 |
| GBM | 0.073 | 4.20E-01 | 9.01E-01 | 7797 | 579.52 |
| READ | 0.147 | 5.20E-01 | 9.71E-01 | 8790 | 1771.38 |
| LIHC | -0.049 | 5.70E-01 | 7.83E-01 | 11514 | 2053.19 |
| OV | -0.014 | 8.50E-01 | 9.77E-01 | 14689 | 838.6 |

**Supplementary Table1.** **Cox regression results for CD155**

Cox regression result of the CD155 gene in TCGA pan-cancer datasets. COX coefficient value, positive value indicates high gene expression predicts poor prognoses of patients, negative value represents high gene expression predicts good prognoses. *p*-value, Kaplan-Meier analysis for each cancer, false discovery rate (FDR) correction. *p*-value < 0.05, FDR < 0.05 was considered statistically significant.

Abbreviations: TCGA The Cancer Genome Atlas; UCEC, uterine corpus endometrial carcinoma; STAD, stomach adenocarcinoma ; SKCM, cutaneous melanoma; SARC, sarcoma; PRAD, prostate adenocarcinoma; PAAD, pancreatic ductal adenocarcinoma; OV, ovarian serous cystadenocarcinoma; LUSC, lung squamous cell carcinoma; LUAD, lung adenocarcinoma; LIHC, liver hepatocellular carcinoma; LGG, lower Grade Glioma; KIRP, papillary kidney carcinoma ; KIRC, clear cell kidney carcinoma; HNSC, head and neck squamous cell carcinoma; GBM, glioblastoma multiforme; ESCA, esophageal cancer; COAD, colon and rectal adenocarcinoma; CECA, cervical cancer; BRCA, breast cancer; BLCA, urothelial bladder cancer; AML, acute myeloid leukemia.

**Supplementary Table2. Kaplan plot for PVR in LUAD**

| Patient | Days | Status | Expression | Group |
| --- | --- | --- | --- | --- |
| TCGA-50-5068 | 1499 | Dead | 295.86 | Low |
| TCGA-67-3770 | 610 | Alive | 394.74 | Low |
| TCGA-64-1676 | 1728 | Alive | 415.03 | Low |
| TCGA-73-7499 | 1531 | Dead | 418.62 | Low |
| TCGA-86-7714 | 625 | Dead | 447.37 | Low |
| TCGA-67-3772 | 573 | Alive | 533.71 | Low |
| TCGA-05-5423 | 151 | Alive | 537.73 | Low |
| TCGA-64-1678 | 1189 | Alive | 538.52 | Low |
| TCGA-55-A48X | 689 | Alive | 538.61 | Low |
| TCGA-97-7552 | 1932 | Alive | 538.97 | Low |
| TCGA-05-5428 | 670 | Alive | 556.35 | Low |
| TCGA-64-1680 | 1126 | Alive | 562.03 | Low |
| TCGA-97-A4LX | 614 | Alive | 567.48 | Low |
| TCGA-05-4396 | 303 | Dead | 571.65 | Low |
| TCGA-50-5055 | 1830 | Dead | 577.24 | Low |
| TCGA-44-3398 | 1163 | Alive | 584.58 | Low |
| TCGA-NJ-A55A | 15 | Alive | 587.46 | Low |
| TCGA-49-4488 | 869 | Dead | 587.78 | Low |
| TCGA-05-4434 | 457 | Dead | 588.29 | Low |
| TCGA-67-3773 | 427 | Alive | 598.15 | Low |
| TCGA-44-6148 | 704 | Alive | 604.79 | Low |
| TCGA-35-5375 | 264 | Alive | 613.76 | Low |
| TCGA-44-2655 | 1324 | Alive | 621.78 | Low |
| TCGA-J2-8192 | 739 | Alive | 626.94 | Low |
| TCGA-91-8496 | 505 | Alive | 627.27 | Low |
| TCGA-50-8460 | 829 | Alive | 631.97 | Low |
| TCGA-86-6851 | 179 | Alive | 632.3 | Low |
| TCGA-L4-A4E5 | 578 | Alive | 633.45 | Low |
| TCGA-44-2657 | 1351 | Alive | 634.23 | Low |
| TCGA-50-8457 | 1125 | Alive | 636.04 | Low |
| TCGA-J2-A4AD | 550 | Dead | 637.84 | Low |
| TCGA-62-A46R | 1725 | Dead | 644.28 | Low |
| TCGA-50-5935 | 653 | Dead | 644.91 | Low |
| TCGA-50-5049 | 3094 | Alive | 647.88 | Low |
| TCGA-55-8085 | 904 | Alive | 648.57 | Low |
| TCGA-97-A4M0 | 652 | Alive | 665.15 | Low |
| TCGA-05-4397 | 731 | Dead | 667.26 | Low |
| TCGA-78-7155 | 1171 | Dead | 675.93 | Low |
| TCGA-95-7947 | 477 | Alive | 678.11 | Low |
| TCGA-99-7458 | 747 | Alive | 680.96 | Low |
| TCGA-35-4122 | 225 | Alive | 681.91 | Low |
| TCGA-44-2661 | 1159 | Alive | 682.52 | Low |
| TCGA-55-1596 | 2065 | Alive | 683.24 | Low |
| TCGA-L9-A50W | 442 | Dead | 691.39 | Low |
| TCGA-44-6779 | 500 | Dead | 692.57 | Low |
| TCGA-55-6972 | 1632 | Dead | 706.62 | Low |
| TCGA-62-A470 | 1194 | Dead | 709.36 | Low |
| TCGA-62-8402 | 1498 | Dead | 710.43 | Low |
| TCGA-55-6712 | 171 | Dead | 712.53 | Low |
| TCGA-62-8397 | 1289 | Alive | 715.33 | Low |
| TCGA-73-7498 | 1189 | Alive | 716.27 | Low |
| TCGA-73-4677 | 38 | Dead | 719.91 | Low |
| TCGA-55-7573 | 487 | Alive | 720.58 | Low |
| TCGA-95-7948 | 476 | Alive | 723.68 | Low |
| TCGA-86-8668 | 423 | Alive | 724.7 | Low |
| TCGA-55-A492 | 596 | Alive | 727.37 | Low |
| TCGA-05-5429 | 275 | Dead | 729.31 | Low |
| TCGA-97-8179 | 435 | Alive | 730.31 | Low |
| TCGA-38-4628 | 1492 | Dead | 730.72 | Low |
| TCGA-78-7160 | 697 | Dead | 736.28 | Low |
| TCGA-86-8671 | 839 | Alive | 736.74 | Low |
| TCGA-44-8120 | 260 | Alive | 739.26 | Low |
| TCGA-86-6562 | 376 | Dead | 749.3 | Low |
| TCGA-50-5066 | 1442 | Alive | 751.74 | Low |
| TCGA-78-7156 | 976 | Dead | 753.73 | Low |
| TCGA-97-A4M6 | 568 | Alive | 753.85 | Low |
| TCGA-44-2656 | 1429 | Alive | 760.87 | Low |
| TCGA-78-7147 | 586 | Dead | 760.98 | Low |
| TCGA-50-5944 | 1750 | Alive | 763.53 | Low |
| TCGA-78-7163 | 7248 | Alive | 765.21 | Low |
| TCGA-55-1592 | 701 | Dead | 768.37 | Low |
| TCGA-86-8280 | 701 | Alive | 769.17 | Low |
| TCGA-67-6217 | 422 | Alive | 769.84 | Low |
| TCGA-78-8655 | 2360 | Alive | 770.11 | Low |
| TCGA-05-4403 | 578 | Alive | 773.42 | Low |
| TCGA-44-5645 | 852 | Alive | 775.1 | Low |
| TCGA-38-7271 | 800 | Dead | 775.25 | Low |
| TCGA-J2-A4AG | 988 | Alive | 776.7 | Low |
| TCGA-78-8648 | 1209 | Dead | 779.22 | Low |
| TCGA-44-3919 | 1026 | Dead | 782.76 | Low |
| TCGA-53-7626 | 929 | Dead | 783.62 | Low |
| TCGA-55-6987 | 2137 | Alive | 793.43 | Low |
| TCGA-55-8301 | 534 | Alive | 793.55 | Low |
| TCGA-78-7536 | 244 | Dead | 793.56 | Low |
| TCGA-78-7149 | 3940 | Alive | 797.9 | Low |
| TCGA-64-5778 | 1305 | Alive | 801.25 | Low |
| TCGA-95-8494 | 84 | Alive | 803.7 | Low |
| TCGA-55-8514 | 520 | Alive | 805.81 | Low |
| TCGA-55-8097 | 476 | Alive | 812.26 | Low |
| TCGA-97-7546 | 1285 | Alive | 814.72 | Low |
| TCGA-44-2659 | 1367 | Alive | 830.01 | Low |
| TCGA-55-8513 | 791 | Alive | 830.12 | Low |
| TCGA-S2-AA1A | 513 | Alive | 830.13 | Low |
| TCGA-55-A48Z | 651 | Alive | 833.25 | Low |
| TCGA-MP-A5C7 | 2248 | Alive | 835.36 | Low |
| TCGA-MP-A4T6 | 1790 | Dead | 837.69 | Low |
| TCGA-64-1679 | 2488 | Alive | 840.39 | Low |
| TCGA-50-5045 | 2174 | Dead | 846.87 | Low |
| TCGA-95-8039 | 830 | Alive | 847.78 | Low |
| TCGA-49-4505 | 428 | Dead | 849 | Low |
| TCGA-97-A4M2 | 624 | Alive | 849.12 | Low |
| TCGA-49-AAR2 | 2224 | Alive | 849.47 | Low |
| TCGA-97-8171 | 568 | Alive | 851.28 | Low |
| TCGA-55-8512 | 607 | Dead | 853.02 | Low |
| TCGA-78-7162 | 3169 | Dead | 854.74 | Low |
| TCGA-44-6777 | 987 | Dead | 854.81 | Low |
| TCGA-78-7143 | 4961 | Dead | 857.49 | Low |
| TCGA-78-7167 | 2681 | Dead | 858.84 | Low |
| TCGA-55-7816 | 468 | Dead | 862.37 | Low |
| TCGA-49-AARR | 4992 | Alive | 868.48 | Low |
| TCGA-38-4627 | 1147 | Dead | 870.49 | Low |
| TCGA-49-4490 | 385 | Dead | 870.54 | Low |
| TCGA-86-7954 | 605 | Alive | 873.17 | Low |
| TCGA-05-4249 | 1523 | Alive | 873.47 | Low |
| TCGA-91-A4BC | 44 | Alive | 878.59 | Low |
| TCGA-MN-A4N5 | 84 | Alive | 879.53 | Low |
| TCGA-49-4494 | 1081 | Dead | 881.66 | Low |
| TCGA-62-8395 | 1216 | Alive | 885.57 | Low |
| TCGA-J2-A4AE | 1079 | Alive | 885.93 | Low |
| TCGA-93-7348 | 531 | Alive | 886.98 | Low |
| TCGA-97-8177 | 499 | Alive | 888.74 | Low |
| TCGA-50-5942 | 1847 | Alive | 890.56 | Low |
| TCGA-55-A4DG | 608 | Alive | 890.82 | Low |
| TCGA-55-7995 | 889 | Alive | 891.15 | Low |
| TCGA-35-3615 | 14 | Alive | 891.16 | Low |
| TCGA-44-7659 | 691 | Alive | 893.12 | Low |
| TCGA-05-4430 | 761 | Alive | 894.74 | Low |
| TCGA-86-8279 | 949 | Alive | 902.52 | Low |
| TCGA-78-7539 | 791 | Alive | 903.09 | Low |
| TCGA-97-A4M7 | 629 | Alive | 904.6 | Low |
| TCGA-55-7727 | 119 | Alive | 906.42 | Low |
| TCGA-73-4662 | 2515 | Alive | 907.93 | Low |
| TCGA-49-4507 | 268 | Dead | 908.68 | Low |
| TCGA-49-4486 | 2318 | Dead | 910.03 | Low |
| TCGA-67-4679 | 448 | Alive | 911.2 | Low |
| TCGA-55-8087 | 462 | Alive | 911.34 | Low |
| TCGA-05-5420 | 457 | Alive | 912.03 | Low |
| TCGA-49-AAQV | 677 | Dead | 912.25 | Low |
| TCGA-78-8662 | 3361 | Dead | 918.6 | Low |
| TCGA-93-A4JO | 33 | Dead | 920.5 | Low |
| TCGA-05-4420 | 912 | Alive | 922.96 | Low |
| TCGA-91-A4BD | 603 | Alive | 925.77 | Low |
| TCGA-44-8117 | 385 | Alive | 926.15 | Low |
| TCGA-93-A4JN | 718 | Alive | 931.01 | Low |
| TCGA-05-4402 | 244 | Dead | 931.3 | Low |
| TCGA-05-4384 | 426 | Alive | 934.64 | Low |
| TCGA-86-A4D0 | 116 | Dead | 939.04 | Low |
| TCGA-55-8091 | 600 | Alive | 939.32 | Low |
| TCGA-50-8459 | 1119 | Alive | 946.11 | Low |
| TCGA-55-7724 | 705 | Alive | 946.89 | Low |
| TCGA-55-7281 | 872 | Alive | 953.28 | Low |
| TCGA-97-7553 | 1870 | Alive | 954.54 | Low |
| TCGA-55-1595 | 1479 | Alive | 966.57 | Low |
| TCGA-50-5941 | 1474 | Alive | 970.51 | Low |
| TCGA-97-8547 | 657 | Alive | 970.56 | Low |
| TCGA-62-A46Y | 414 | Dead | 971.43 | Low |
| TCGA-86-7701 | 947 | Alive | 975.76 | Low |
| TCGA-91-6830 | 60 | Alive | 977.01 | Low |
| TCGA-91-7771 | 492 | Alive | 980 | Low |
| TCGA-MN-A4N1 | 827 | Alive | 981.82 | Low |
| TCGA-55-8511 | 552 | Alive | 982.76 | Low |
| TCGA-86-8074 | 24 | Alive | 984.63 | Low |
| TCGA-86-8076 | 993 | Alive | 986.55 | Low |
| TCGA-50-6594 | 370 | Dead | 988.73 | Low |
| TCGA-49-4501 | 1421 | Dead | 990.22 | Low |
| TCGA-55-7728 | 704 | Alive | 990.92 | Low |
| TCGA-55-6980 | 2109 | Alive | 991.16 | Low |
| TCGA-55-6970 | 464 | Dead | 992.29 | Low |
| TCGA-44-6146 | 728 | Alive | 992.33 | Low |
| TCGA-55-7570 | 824 | Alive | 992.59 | Low |
| TCGA-55-A491 | 626 | Alive | 1000.53 | Low |
| TCGA-55-8208 | 674 | Alive | 1004.13 | Low |
| TCGA-78-8640 | 7062 | Alive | 1004.23 | Low |
| TCGA-71-6725 | 256 | Alive | 1007.84 | Low |
| TCGA-L4-A4E6 | 435 | Alive | 1011.16 | Low |
| TCGA-91-6829 | 1258 | Dead | 1013.42 | Low |
| TCGA-44-3396 | 1130 | Alive | 1018.8 | Low |
| TCGA-44-5643 | 1013 | Alive | 1045.63 | Low |
| TCGA-55-8089 | 702 | Dead | 1047.47 | Low |
| TCGA-44-4112 | 808 | Dead | 1048.59 | Low |
| TCGA-38-6178 | 448 | Alive | 1049.14 | Low |
| TCGA-91-6847 | 842 | Alive | 1049.69 | Low |
| TCGA-99-8028 | 1118 | Alive | 1057.7 | Low |
| TCGA-05-4424 | 913 | Alive | 1059.1 | Low |
| TCGA-55-7283 | 609 | Alive | 1061.15 | Low |
| TCGA-64-1681 | 1167 | Dead | 1061.9 | Low |
| TCGA-55-A57B | 546 | Alive | 1065.23 | Low |
| TCGA-95-7562 | 87 | Dead | 1065.98 | Low |
| TCGA-64-5779 | 864 | Alive | 1066.28 | Low |
| TCGA-O1-A52J | 1798 | Dead | 1066.99 | Low |
| TCGA-91-6828 | 323 | Alive | 1072.39 | Low |
| TCGA-55-7994 | 603 | Alive | 1076.79 | Low |
| TCGA-44-7672 | 719 | Alive | 1077.7 | Low |
| TCGA-97-7554 | 775 | Alive | 1078.54 | Low |
| TCGA-44-6147 | 845 | Alive | 1083.61 | Low |
| TCGA-MP-A4TI | 429 | Dead | 1087.63 | Low |
| TCGA-MP-A4SW | 1778 | Dead | 1088.34 | Low |
| TCGA-62-A46V | 2199 | Alive | 1090.82 | Low |
| TCGA-NJ-A4YQ | 1432 | Alive | 1094.79 | Low |
| TCGA-55-8621 | 515 | Alive | 1096.02 | Low |
| TCGA-55-8206 | 888 | Alive | 1097.76 | Low |
| TCGA-49-4512 | 905 | Dead | 1101.46 | Low |
| TCGA-55-8207 | 977 | Alive | 1102.07 | Low |
| TCGA-93-7347 | 683 | Alive | 1104.51 | Low |
| TCGA-55-6975 | 118 | Dead | 1105.62 | Low |
| TCGA-62-A46U | 2067 | Alive | 1109.33 | Low |
| TCGA-49-6742 | 1678 | Alive | 1110.37 | Low |
| TCGA-86-A4P8 | 805 | Alive | 1111.67 | Low |
| TCGA-44-A47G | 351 | Alive | 1115.65 | Low |
| TCGA-38-4629 | 864 | Dead | 1115.78 | Low |
| TCGA-44-6775 | 705 | Alive | 1116.1 | Low |
| TCGA-55-8616 | 48 | Alive | 1116.6 | Low |
| TCGA-44-A4SU | 409 | Dead | 1117.03 | Low |
| TCGA-38-4630 | 1073 | Dead | 1126.08 | Low |
| TCGA-55-8619 | 416 | Alive | 1128.71 | Low |
| TCGA-91-8499 | 36 | Alive | 1130.7 | Low |
| TCGA-67-3771 | 610 | Alive | 1132.25 | Low |
| TCGA-73-4666 | 800 | Alive | 1132.37 | Low |
| TCGA-49-AAR3 | 1893 | Alive | 1132.4 | Low |
| TCGA-95-7039 | 1272 | Alive | 1134.29 | Low |
| TCGA-44-A4SS | 415 | Alive | 1135.38 | Low |
| TCGA-97-A4M1 | 601 | Alive | 1139.99 | Low |
| TCGA-50-5051 | 478 | Dead | 1140.2 | Low |
| TCGA-91-8497 | 434 | Dead | 1140.49 | Low |
| TCGA-97-8174 | 164 | Dead | 1147.35 | Low |
| TCGA-78-7535 | 949 | Dead | 1147.54 | Low |
| TCGA-44-6778 | 1864 | Alive | 1148.48 | Low |
| TCGA-97-8172 | 545 | Alive | 1151.1 | Low |
| TCGA-93-A4JP | 578 | Alive | 1158.21 | Low |
| TCGA-50-6593 | 336 | Dead | 1161.64 | Low |
| TCGA-44-7671 | 889 | Alive | 1161.72 | Low |
| TCGA-49-6744 | 1683 | Alive | 1161.91 | Low |
| TCGA-05-4417 | 455 | Alive | 1162.11 | Low |
| TCGA-86-8672 | 19 | Dead | 1163.55 | Low |
| TCGA-99-8033 | 656 | Dead | 1167.19 | Low |
| TCGA-86-A4P7 | 415 | Alive | 1167.93 | Low |
| TCGA-97-7938 | 18 | Dead | 1169.18 | Low |
| TCGA-55-6985 | 1233 | Alive | 1170.9 | Low |
| TCGA-49-AARN | 1135 | Dead | 1172.39 | Low |
| TCGA-71-8520 | 210 | Dead | 1174.26 | Low |
| TCGA-NJ-A4YF | 2161 | Alive | 1174.36 | Low |
| TCGA-50-5939 | 460 | Dead | 1178.72 | Low |
| TCGA-44-3918 | 1036 | Alive | 1179.25 | Low |
| TCGA-55-8299 | 469 | Dead | 1185.16 | Low |
| TCGA-67-6216 | 141 | Alive | 1186.98 | Low |
| TCGA-73-4670 | 131 | Alive | 1192.29 | Low |
| TCGA-49-AAR0 | 4765 | Alive | 1192.36 | High |
| TCGA-86-7953 | 997 | Alive | 1193.59 | High |
| TCGA-NJ-A4YP | 50 | Alive | 1195.38 | High |
| TCGA-38-A44F | 133 | Alive | 1202.63 | High |
| TCGA-78-7158 | 179 | Dead | 1207.05 | High |
| TCGA-78-7537 | 1622 | Dead | 1211.84 | High |
| TCGA-86-8585 | 353 | Alive | 1211.99 | High |
| TCGA-44-A47A | 466 | Alive | 1214.94 | High |
| TCGA-97-8552 | 626 | Alive | 1223.17 | High |
| TCGA-55-7725 | 442 | Alive | 1230.04 | High |
| TCGA-55-8096 | 719 | Dead | 1235.86 | High |
| TCGA-50-6591 | 119 | Dead | 1235.96 | High |
| TCGA-L9-A444 | 307 | Alive | 1236.9 | High |
| TCGA-86-A456 | 896 | Alive | 1240.28 | High |
| TCGA-69-7980 | 362 | Alive | 1242.9 | High |
| TCGA-49-4514 | 1700 | Alive | 1250.18 | High |
| TCGA-MP-A4T8 | 161 | Dead | 1252.28 | High |
| TCGA-99-8032 | 44 | Alive | 1252.76 | High |
| TCGA-05-4405 | 610 | Alive | 1257.67 | High |
| TCGA-91-6849 | 35 | Alive | 1259.18 | High |
| TCGA-MP-A4T7 | 167 | Dead | 1259.38 | High |
| TCGA-MP-A4TJ | 339 | Dead | 1264.63 | High |
| TCGA-55-7227 | 952 | Dead | 1279.89 | High |
| TCGA-97-A4M5 | 634 | Alive | 1285.18 | High |
| TCGA-55-8092 | 154 | Dead | 1287.43 | High |
| TCGA-NJ-A7XG | 617 | Alive | 1292.27 | High |
| TCGA-55-7914 | 187 | Dead | 1294.35 | High |
| TCGA-55-6983 | 2823 | Alive | 1298.23 | High |
| TCGA-55-7574 | 995 | Dead | 1298.67 | High |
| TCGA-MP-A4TA | 950 | Dead | 1304.75 | High |
| TCGA-NJ-A4YG | 2261 | Alive | 1310.62 | High |
| TCGA-44-6774 | 658 | Alive | 1311.34 | High |
| TCGA-69-7765 | 165 | Alive | 1311.49 | High |
| TCGA-78-7159 | 1974 | Alive | 1312.4 | High |
| TCGA-44-7669 | 574 | Dead | 1313.84 | High |
| TCGA-49-4506 | 999 | Dead | 1314.1 | High |
| TCGA-49-6745 | 522 | Alive | 1318.43 | High |
| TCGA-86-8278 | 944 | Alive | 1324.17 | High |
| TCGA-73-4676 | 281 | Dead | 1325.07 | High |
| TCGA-50-5946 | 1617 | Alive | 1327.76 | High |
| TCGA-05-4425 | 669 | Alive | 1329.04 | High |
| TCGA-38-4626 | 3674 | Alive | 1337.71 | High |
| TCGA-69-8255 | 129 | Alive | 1338.14 | High |
| TCGA-55-8506 | 11 | Alive | 1342.91 | High |
| TCGA-67-6215 | 174 | Alive | 1345.2 | High |
| TCGA-55-7911 | 537 | Alive | 1349.02 | High |
| TCGA-55-1594 | 1178 | Alive | 1358.88 | High |
| TCGA-93-8067 | 186 | Alive | 1362.67 | High |
| TCGA-55-7284 | 243 | Dead | 1363.84 | High |
| TCGA-64-5815 | 866 | Alive | 1364.49 | High |
| TCGA-55-8620 | 375 | Dead | 1365.08 | High |
| TCGA-MP-A4TF | 336 | Dead | 1367.05 | High |
| TCGA-05-5715 | 62 | Alive | 1368.73 | High |
| TCGA-44-6145 | 595 | Alive | 1373.34 | High |
| TCGA-55-8510 | 539 | Alive | 1375.77 | High |
| TCGA-55-8203 | 547 | Alive | 1383.48 | High |
| TCGA-95-A4VP | 605 | Alive | 1386.45 | High |
| TCGA-50-5931 | 434 | Dead | 1387.63 | High |
| TCGA-55-A494 | 481 | Alive | 1389.79 | High |
| TCGA-55-8204 | 515 | Alive | 1398.61 | High |
| TCGA-05-4389 | 1369 | Alive | 1411.41 | High |
| TCGA-MP-A4SV | 2620 | Dead | 1413 | High |
| TCGA-55-7903 | 567 | Alive | 1415.05 | High |
| TCGA-86-A4JF | 737 | Dead | 1417.4 | High |
| TCGA-55-8302 | 478 | Alive | 1421.57 | High |
| TCGA-4B-A93V | 300 | Dead | 1423.58 | High |
| TCGA-86-8674 | 806 | Alive | 1423.83 | High |
| TCGA-55-7910 | 1040 | Alive | 1425 | High |
| TCGA-69-8453 | 788 | Alive | 1430.8 | High |
| TCGA-55-6978 | 176 | Dead | 1434.9 | High |
| TCGA-05-4426 | 791 | Alive | 1445.87 | High |
| TCGA-55-6642 | 2449 | Alive | 1446.92 | High |
| TCGA-78-7148 | 626 | Dead | 1447.49 | High |
| TCGA-78-7166 | 258 | Dead | 1453.86 | High |
| TCGA-86-8669 | 938 | Alive | 1456.84 | High |
| TCGA-78-7633 | 1528 | Dead | 1458.54 | High |
| TCGA-55-6968 | 1293 | Dead | 1461.93 | High |
| TCGA-99-AA5R | 658 | Alive | 1470.14 | High |
| TCGA-35-4123 | 182 | Alive | 1474.53 | High |
| TCGA-69-A59K | 522 | Alive | 1477.86 | High |
| TCGA-64-1677 | 628 | Dead | 1478.12 | High |
| TCGA-53-A4EZ | 1071 | Alive | 1487.56 | High |
| TCGA-55-A4DF | 614 | Dead | 1492.83 | High |
| TCGA-86-7955 | 1072 | Alive | 1500.11 | High |
| TCGA-69-7761 | 186 | Alive | 1500.27 | High |
| TCGA-97-8176 | 468 | Dead | 1501.41 | High |
| TCGA-55-7726 | 652 | Alive | 1509.32 | High |
| TCGA-55-6543 | 435 | Alive | 1511.71 | High |
| TCGA-44-5644 | 863 | Alive | 1514.21 | High |
| TCGA-97-7937 | 564 | Alive | 1523.5 | High |
| TCGA-49-6767 | 677 | Alive | 1527.81 | High |
| TCGA-05-4432 | 761 | Alive | 1532.16 | High |
| TCGA-44-2665 | 1301 | Alive | 1535.58 | High |
| TCGA-95-A4VN | 553 | Alive | 1538.68 | High |
| TCGA-05-4422 | 365 | Alive | 1538.81 | High |
| TCGA-49-4510 | 896 | Dead | 1540.23 | High |
| TCGA-50-5072 | 250 | Dead | 1549.19 | High |
| TCGA-86-8073 | 740 | Alive | 1558.69 | High |
| TCGA-95-A4VK | 651 | Alive | 1560.76 | High |
| TCGA-J2-8194 | 724 | Alive | 1563.45 | High |
| TCGA-55-6986 | 3261 | Alive | 1568.31 | High |
| TCGA-44-6776 | 2616 | Alive | 1573.99 | High |
| TCGA-50-5930 | 282 | Dead | 1575.07 | High |
| TCGA-50-5936 | 257 | Dead | 1577.49 | High |
| TCGA-55-6982 | 995 | Dead | 1581.01 | High |
| TCGA-55-7576 | 670 | Alive | 1584.61 | High |
| TCGA-49-AARQ | 6732 | Alive | 1596.07 | High |
| TCGA-78-7152 | 1215 | Dead | 1602.6 | High |
| TCGA-NJ-A55R | 603 | Alive | 1604.61 | High |
| TCGA-38-4631 | 354 | Dead | 1606.4 | High |
| TCGA-91-6831 | 310 | Alive | 1606.79 | High |
| TCGA-L9-A7SV | 565 | Alive | 1611.84 | High |
| TCGA-97-A4M3 | 540 | Alive | 1613.05 | High |
| TCGA-38-4625 | 2973 | Alive | 1613.35 | High |
| TCGA-73-4659 | 711 | Dead | 1615.61 | High |
| TCGA-73-4658 | 1600 | Dead | 1618.53 | High |
| TCGA-55-6969 | 1239 | Alive | 1626.44 | High |
| TCGA-73-A9RS | 340 | Dead | 1633.8 | High |
| TCGA-97-8175 | 551 | Alive | 1635.48 | High |
| TCGA-55-8508 | 617 | Alive | 1638.37 | High |
| TCGA-MP-A4SY | 1501 | Dead | 1649.46 | High |
| TCGA-49-AARO | 3759 | Alive | 1663.88 | High |
| TCGA-69-8253 | 426 | Alive | 1670.84 | High |
| TCGA-73-4675 | 922 | Dead | 1673.79 | High |
| TCGA-05-4433 | 730 | Alive | 1676.03 | High |
| TCGA-44-8119 | 285 | Alive | 1689.75 | High |
| TCGA-69-7760 | 202 | Alive | 1697.69 | High |
| TCGA-62-A471 | 1246 | Alive | 1697.7 | High |
| TCGA-95-7944 | 377 | Alive | 1698.39 | High |
| TCGA-55-7913 | 561 | Dead | 1715.47 | High |
| TCGA-73-4668 | 467 | Alive | 1719.07 | High |
| TCGA-55-8205 | 599 | Alive | 1723.45 | High |
| TCGA-44-7667 | 1097 | Alive | 1727.28 | High |
| TCGA-55-6981 | 1379 | Dead | 1729.82 | High |
| TCGA-55-7815 | 773 | Alive | 1731.46 | High |
| TCGA-MP-A4T9 | 1265 | Dead | 1733.17 | High |
| TCGA-97-7941 | 484 | Alive | 1759.63 | High |
| TCGA-78-7161 | 291 | Dead | 1762.18 | High |
| TCGA-44-A479 | 486 | Alive | 1779.8 | High |
| TCGA-95-7043 | 503 | Dead | 1784.83 | High |
| TCGA-86-8055 | 124 | Dead | 1785.68 | High |
| TCGA-64-5774 | 2676 | Alive | 1794.1 | High |
| TCGA-55-6984 | 760 | Dead | 1803.47 | High |
| TCGA-MP-A4TE | 896 | Dead | 1818.94 | High |
| TCGA-MP-A4TD | 307 | Dead | 1824.54 | High |
| TCGA-69-7763 | 690 | Alive | 1851.93 | High |
| TCGA-49-4487 | 855 | Dead | 1852.57 | High |
| TCGA-86-8075 | 694 | Dead | 1854.31 | High |
| TCGA-50-6590 | 1288 | Dead | 1857.07 | High |
| TCGA-78-7145 | 826 | Dead | 1866.22 | High |
| TCGA-55-A48Y | 630 | Alive | 1879.73 | High |
| TCGA-44-7660 | 592 | Alive | 1901.8 | High |
| TCGA-55-5899 | 930 | Alive | 1902.56 | High |
| TCGA-69-7979 | 408 | Alive | 1908.67 | High |
| TCGA-69-7974 | 184 | Alive | 1909.85 | High |
| TCGA-50-6592 | 777 | Dead | 1915.51 | High |
| TCGA-67-3774 | 385 | Alive | 1918.57 | High |
| TCGA-44-7661 | 557 | Dead | 1926.61 | High |
| TCGA-05-4382 | 607 | Alive | 1933.58 | High |
| TCGA-55-8505 | 440 | Alive | 1935.77 | High |
| TCGA-55-7907 | 343 | Dead | 1949.96 | High |
| TCGA-55-8615 | 446 | Alive | 1954.01 | High |
| TCGA-05-5425 | 882 | Alive | 1954.75 | High |
| TCGA-50-5932 | 1235 | Dead | 1963.42 | High |
| TCGA-62-8398 | 444 | Dead | 1967.84 | High |
| TCGA-L9-A443 | 193 | Dead | 1975.57 | High |
| TCGA-62-A472 | 910 | Alive | 1976.83 | High |
| TCGA-44-A47B | 287 | Alive | 1982.09 | High |
| TCGA-86-8359 | 444 | Dead | 1983.6 | High |
| TCGA-05-4398 | 1431 | Alive | 1990 | High |
| TCGA-91-6840 | 372 | Alive | 1998.06 | High |
| TCGA-86-8054 | 1148 | Alive | 2004.5 | High |
| TCGA-55-6979 | 237 | Dead | 2005.55 | High |
| TCGA-L9-A8F4 | 476 | Alive | 2030.66 | High |
| TCGA-MP-A4T4 | 2617 | Dead | 2036.73 | High |
| TCGA-49-AARE | 1229 | Dead | 2084.66 | High |
| TCGA-44-2666 | 97 | Dead | 2091.54 | High |
| TCGA-53-7813 | 424 | Alive | 2095.58 | High |
| TCGA-86-8056 | 139 | Alive | 2101.16 | High |
| TCGA-50-6673 | 22 | Dead | 2109.93 | High |
| TCGA-69-8254 | 409 | Alive | 2132.76 | High |
| TCGA-NJ-A55O | 13 | Alive | 2140.61 | High |
| TCGA-MP-A4TK | 582 | Dead | 2162.95 | High |
| TCGA-05-4390 | 1126 | Alive | 2163.82 | High |
| TCGA-78-7153 | 3635 | Alive | 2168.87 | High |
| TCGA-95-7567 | 568 | Alive | 2169.62 | High |
| TCGA-86-8358 | 653 | Alive | 2182.97 | High |
| TCGA-50-7109 | 308 | Dead | 2202.19 | High |
| TCGA-55-8090 | 598 | Dead | 2206.26 | High |
| TCGA-91-6835 | 79 | Alive | 2209.03 | High |
| TCGA-62-8394 | 139 | Dead | 2210.21 | High |
| TCGA-55-8507 | 418 | Alive | 2228.9 | High |
| TCGA-78-7540 | 1197 | Dead | 2239.6 | High |
| TCGA-MP-A4TH | 741 | Alive | 2273.06 | High |
| TCGA-49-6743 | 1621 | Alive | 2322.3 | High |
| TCGA-91-6836 | 417 | Alive | 2325.98 | High |
| TCGA-55-8094 | 541 | Alive | 2344.21 | High |
| TCGA-44-7662 | 218 | Alive | 2349.69 | High |
| TCGA-62-A46P | 594 | Dead | 2351.88 | High |
| TCGA-78-7542 | 321 | Dead | 2354.04 | High |
| TCGA-93-A4JQ | 526 | Alive | 2397.99 | High |
| TCGA-MN-A4N4 | 1175 | Alive | 2425.39 | High |
| TCGA-78-8660 | 321 | Dead | 2441.76 | High |
| TCGA-62-A46S | 1653 | Dead | 2443.71 | High |
| TCGA-MP-A4TC | 74 | Dead | 2467.7 | High |
| TCGA-49-AAR4 | 879 | Dead | 2484.24 | High |
| TCGA-NJ-A4YI | 4 | Dead | 2499.5 | High |
| TCGA-69-7764 | 414 | Alive | 2523.87 | High |
| TCGA-64-5781 | 1559 | Alive | 2536.98 | High |
| TCGA-L9-A743 | 664 | Alive | 2602.46 | High |
| TCGA-69-7973 | 230 | Alive | 2610.31 | High |
| TCGA-L9-A5IP | 58 | Dead | 2627.08 | High |
| TCGA-78-7154 | 593 | Dead | 2655.75 | High |
| TCGA-05-4427 | 791 | Alive | 2683.65 | High |
| TCGA-97-7547 | 1965 | Alive | 2688.7 | High |
| TCGA-49-AAR9 | 260 | Dead | 2701.24 | High |
| TCGA-69-7978 | 134 | Alive | 2714.19 | High |
| TCGA-55-A490 | 99 | Dead | 2741.01 | High |
| TCGA-49-6761 | 354 | Alive | 2760.04 | High |
| TCGA-86-8673 | 862 | Alive | 2783 | High |
| TCGA-78-7220 | 807 | Dead | 2834.13 | High |
| TCGA-86-7711 | 1046 | Dead | 2869.92 | High |
| TCGA-86-7713 | 1157 | Alive | 2903.14 | High |
| TCGA-44-2662 | 1280 | Alive | 2914.44 | High |
| TCGA-50-5933 | 2393 | Dead | 2921.76 | High |
| TCGA-55-8614 | 536 | Alive | 2930.69 | High |
| TCGA-99-8025 | 1060 | Alive | 2967.01 | High |
| TCGA-62-8399 | 2696 | Alive | 3069.59 | High |
| TCGA-50-5044 | 624 | Dead | 3138.18 | High |
| TCGA-83-5908 | 824 | Alive | 3170.11 | High |
| TCGA-78-7146 | 173 | Dead | 3212.44 | High |
| TCGA-05-4415 | 91 | Dead | 3312 | High |
| TCGA-53-7624 | 1043 | Dead | 3328.5 | High |
| TCGA-50-6595 | 189 | Dead | 3363.34 | High |
| TCGA-78-7150 | 666 | Dead | 3651.18 | High |
| TCGA-44-2668 | 761 | Dead | 3675.2 | High |
| TCGA-05-4418 | 274 | Dead | 3861.07 | High |
| TCGA-64-5775 | 62 | Dead | 3917.08 | High |
| TCGA-44-7670 | 882 | Alive | 3948.37 | High |
| TCGA-91-6848 | 224 | Alive | 3963.76 | High |
| TCGA-50-6597 | 1268 | Dead | 3986.8 | High |
| TCGA-62-A46O | 1454 | Dead | 4294.31 | High |
| TCGA-05-4250 | 121 | Dead | 4413.16 | High |
| TCGA-55-A493 | 28 | Alive | 4606.71 | High |
| TCGA-55-6971 | 1400 | Alive | 4661.18 | High |
| TCGA-38-4632 | 1357 | Dead | 7249.86 | High |

Supplementary Table3. Primer sequences for qRT-PCR.

| **Genes** | **Forward Primer (5’-3’)** | **Reverse Primer (5’-3’)** |
| --- | --- | --- |
| *hCDH1* | GCCTCCTGAAAAGAGAGTGGAAG | TGGCAGTGTCTCTCCAAATCCG |
| *hCDH2* | CCTCCAGAGTTTACTGCCATGAC | GTAGGATCTCCGCCACTGATTC |
| *hDSP* | TGACAGACCGCTGGCAAAGGAT | GGCGTTTAGCATCATAGAGCCAC |
| *hTJP1* | GTCCAGAATCTCGGAAAAGTGCC | CTTTCAGCGCACCATACCAACC |
| *hSNAIL (SNAI1)* | TGCCCTCAAGATGCACATCCGA | GGGACAGGAGAAGGGCTTCTC |
| *hSLUG (SNAI2)* | ATCTGCGGCAAGGCGTTTTCCA | GAGCCCTCAGATTTGACCTGTC |
| *hEpCAM* | GCCAGTGTACTTCAGTTGGTGC | CCCTTCAGGTTTTGCTCTTCTCC |
| *hFN1* | ACAACACCGAGGTGACTGAGAC | GGACACAACGATGCTTCCTGAG |
| *hTwist1* | GCCAGGTACATCGACTTCCTCT | TCCATCCTCCAGACCGAGAAGG |
| *hZeb2* | AATGCACAGAGTGTGGCAAGGC | CTGCTGATGTGCGAACTGTAGG |
| *hVimentin* | AGGCAAAGCAGGAGTCCACTGA | ATCTGGCGTTCCAGGGACTCAT |
| *hASMA* | AGGTCATCACCATCGGCAACGA | GCTGTTGTAGGTGGTCTCGTGA |
| *hβGAPDH* | AACGGGAAGCTTGTCATCAATGGAAA | GCATCAGCAGAGGGGGCAGAG |

Supplementary Table4. Primer sequences for conventional RT-PCR.

| **Genes** | **Forward Primer (5’-3’)** | **Reverse Primer (5’-3’)** |
| --- | --- | --- |
| *CD5* | TCCTGTGGACAAACCA ATCAACAC | - |
| *CD15* | - | GAGGCGCTGGCATGCTCTGT |
| *hβ-actin* | AAGAGCTATGAGCTGCCTGA | TACGGATGTCAACGTCACAC |

**References**

1 Ponten, F., Jirstrom, K. & Uhlen, M. The Human Protein Atlas--a tool for pathology. *J Pathol*. **216**, 387-393, (2008).

2 Yi, S. *et al.* Advancing pancreatic cancer therapy by mesothelin-specific nanobody conjugation. *Mol Cancer*. **24**, 124, (2025).

3 Illergard, K., Ardell, D. H. & Elofsson, A. Structure is three to ten times more conserved than sequence--a study of structural response in protein cores. *Proteins*. **77**, 499-508, (2009).

4 Deuss, F. A. *et al.* Structural Basis for CD96 Immune Receptor Recognition of Nectin-like Protein-5, CD155. *Structure*. **27**, 219-228 e213, (2019).

5 Gray, J. J. *et al.* Protein-protein docking with simultaneous optimization of rigid-body displacement and side-chain conformations. *J Mol Biol*. **331**, 281-299, (2003).

6 Alford, R. F. *et al.* The Rosetta All-Atom Energy Function for Macromolecular Modeling and Design. *J Chem Theory Comput*. **13**, 3031-3048, (2017).

7 Ma, Y., Sadoqi, M. & Shao, J. Biodistribution of indocyanine green-loaded nanoparticles with surface modifications of PEG and folic acid. *Int J Pharm*. **436**, 25-31, (2012).

8 Koessinger, A. L. *et al.* Quantitative in vivo bioluminescence imaging of orthotopic patient-derived glioblastoma xenografts. *Sci Rep*. **10**, 15361, (2020).

9 Baury, B. *et al.* Identification of secreted CD155 isoforms. *Biochem Biophys Res Commun*. **309**, 175-182, (2003).
