# Supplementary material for: Targeting CD155 in lung adenocarcinoma: A5 nanobody-based therapeutics for precision treatment and enhanced drug delivery
Source: Signal Transduct Target Ther. 2025 Jul 10;10:218. doi: 10.1038/s41392-025-02301-z (PMC12241526; doi:10.1038/s41392-025-02301-z)
Supplement: Supplementary file 2 — WB uncut [file 41392_2025_2301_MOESM2_ESM.pdf]

Full blot

Fig. 2c

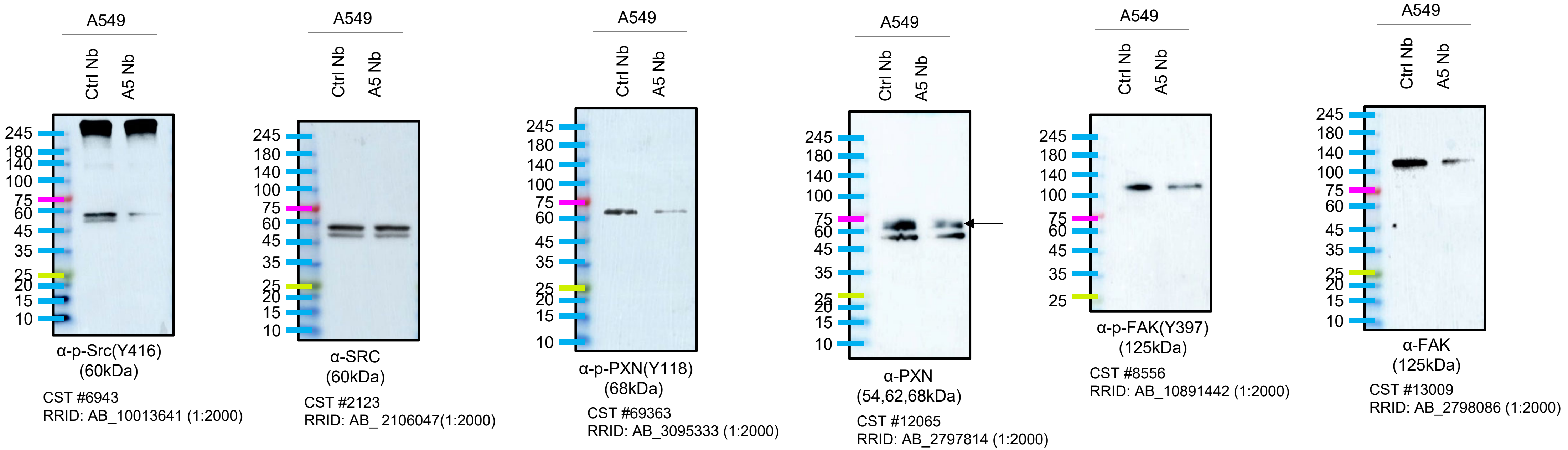

Fig. 2C. Uncut images

Fig. 2c

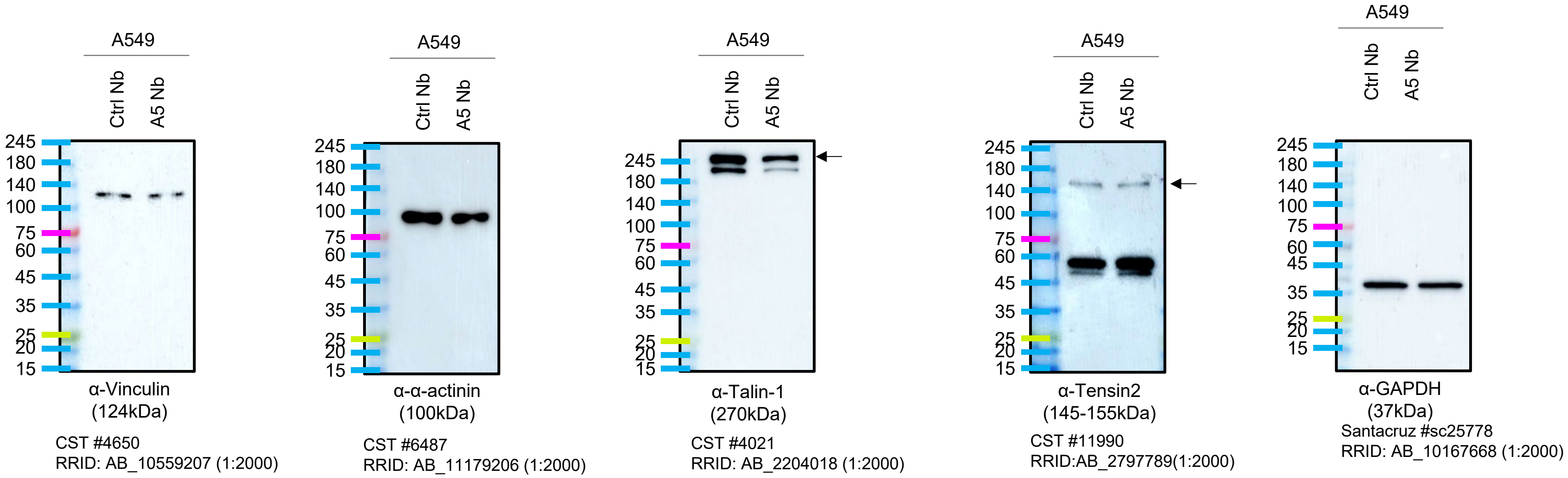

Fig. 2C. Uncut images

Fig. 2C

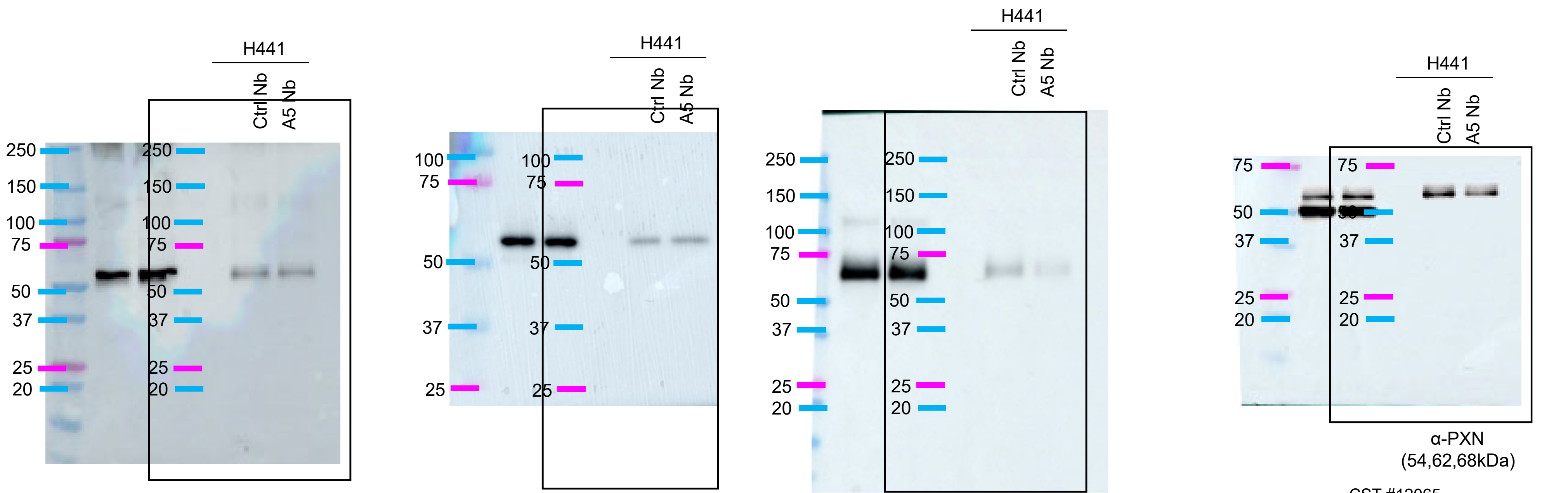

α-p-Src(Y416)
(60kDa)
CST #6943
RRID: AB_10013641 (1:2000)

α-SRC
(60kDa)
CST #2123
RRID: AB_ 2106047(1:2000)

α-p-PXN(Y118)
(68kDa)
CST #69363
RRID: AB_3095333 (1:2000)

CST #12065
RRID: AB_2797814 (1:2000)

Fig. 2C. Uncut images

Fig. 2c

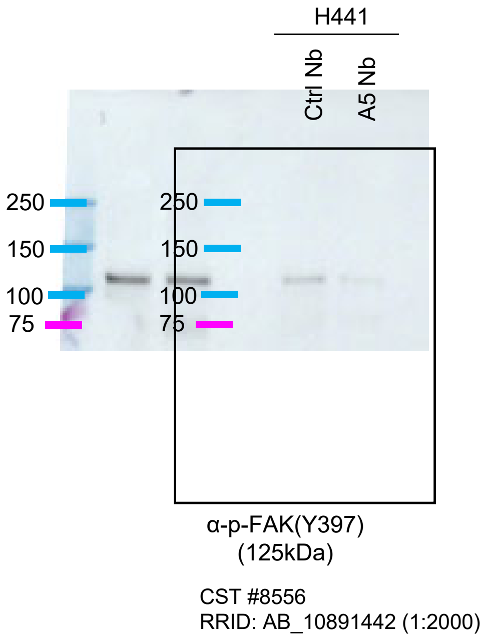

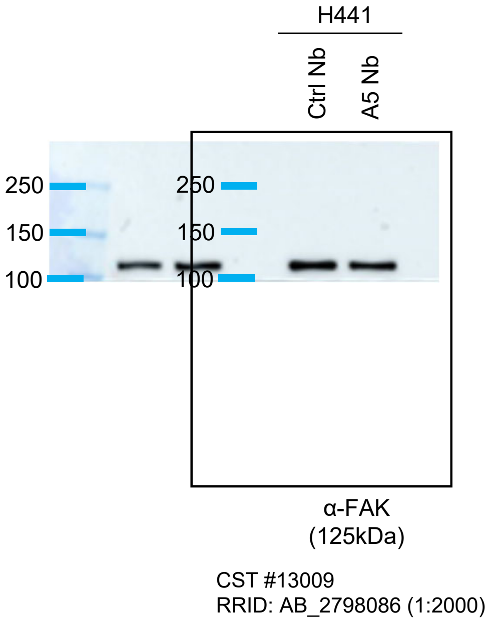

Fig. 2C. Uncut images

Fig. 2c

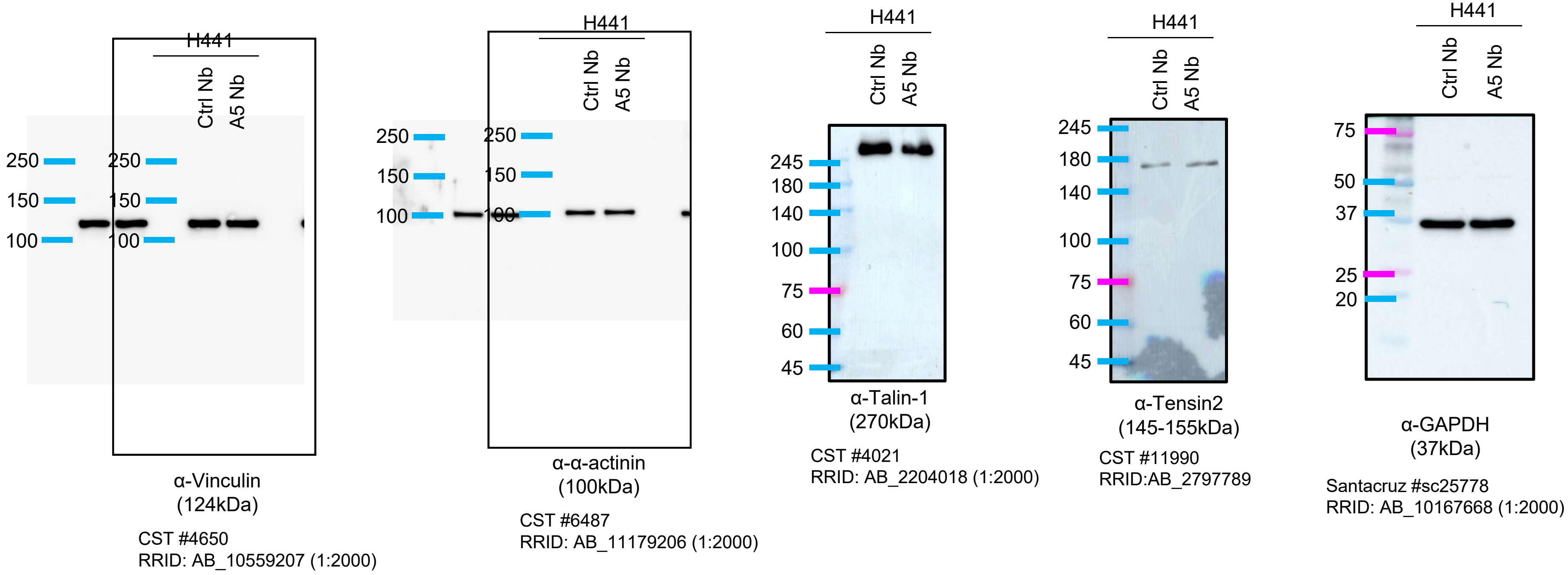

Fig. 2C. Uncut images

Fig.4.i

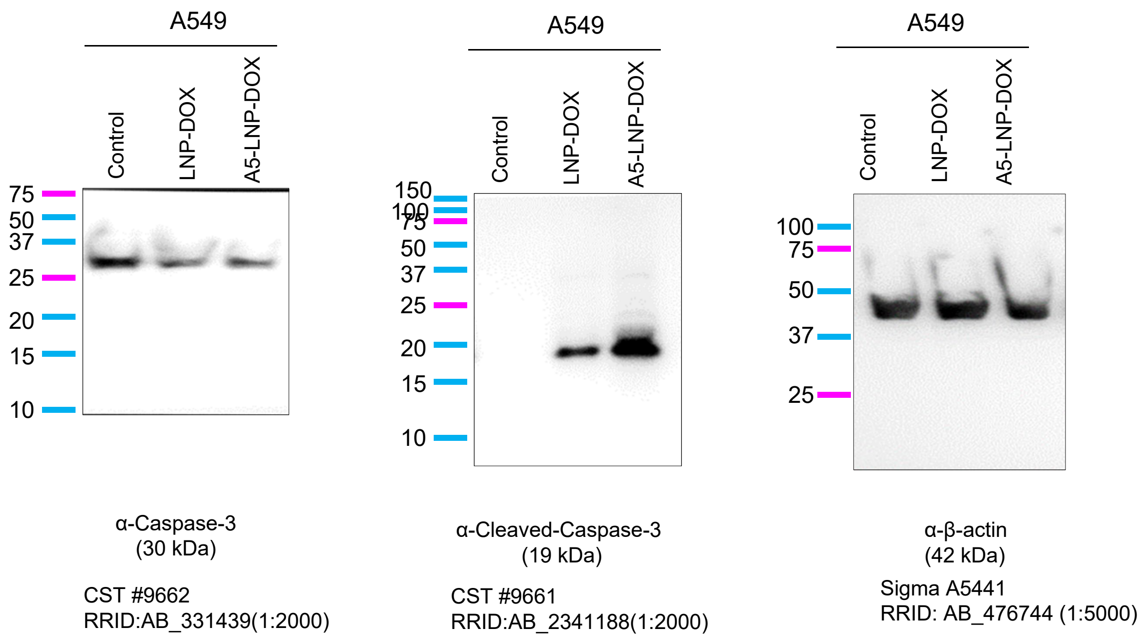

Fig. 4. Uncut images

Fig.6f

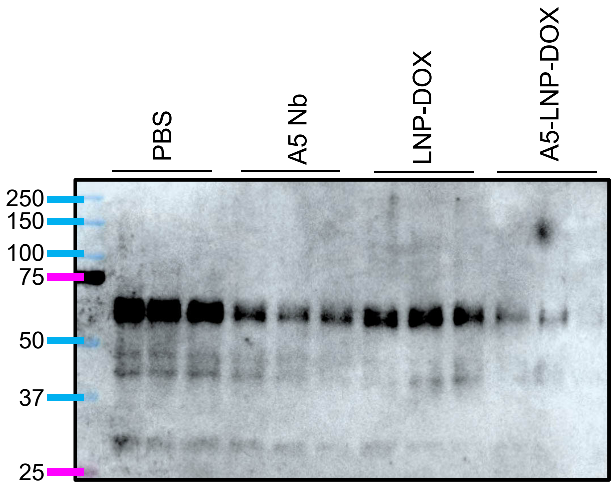

α-PXN
(54,62,68kDa)

CST #12065
RRID: AB_2797814 (1:2000)

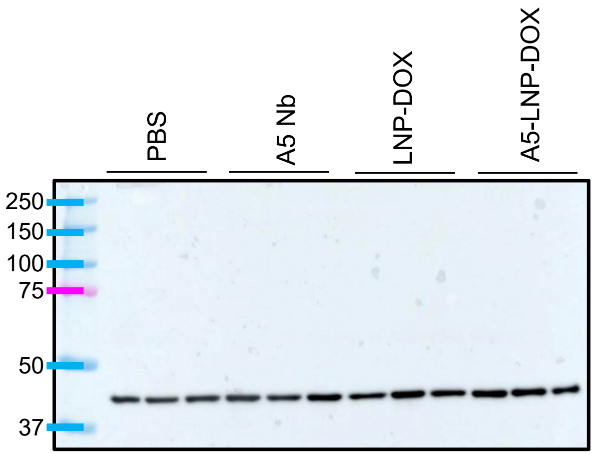

α-β-actin
(42 kDa)

Sigma A5441
RRID: AB_476744 (1:5000)

Fig. 6. Uncut images

Supple Fig. 3C

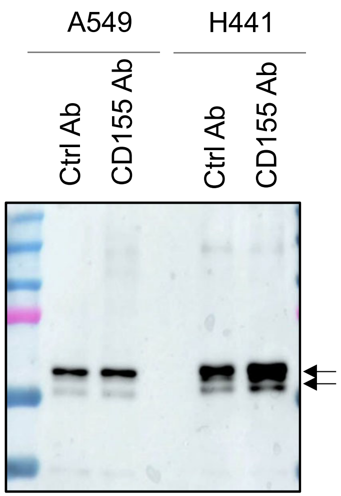

α-p-Src(Y416)
(60kDa)

CST #6943
RRID: AB_10013641 (1:2000)

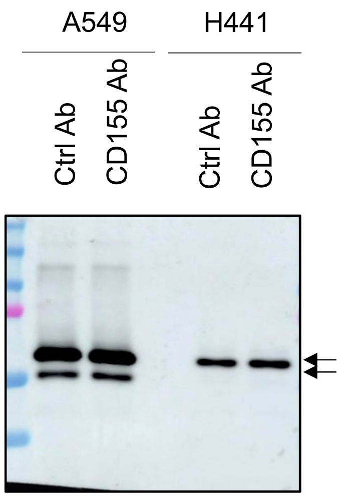

α-SRC
(60kDa)

CST #2123
RRID: AB_ 2106047(1:2000)

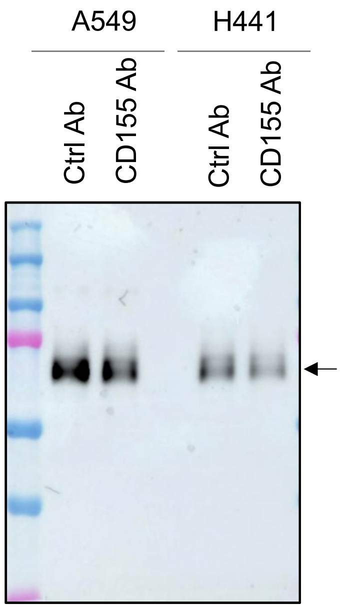

α-p-PXN(Y118)
(68kDa)

CST #69363
RRID: AB_3095333 (1:2000)

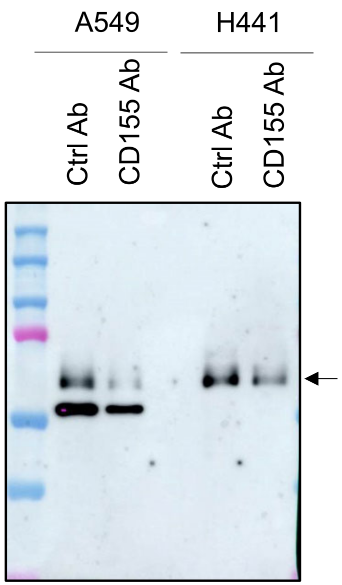

α-PXN
(54,62,68kDa)

CST #12065
RRID: AB_2797814 (1:2000)

Supple Fig. 3C. Uncut images

Supple Fig. 3C

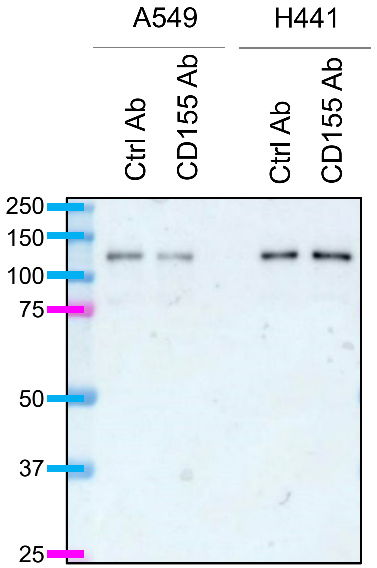

α-p-FAK(Y397)
(125kDa)

CST #8556
RRID: AB_10891442 (1:2000)

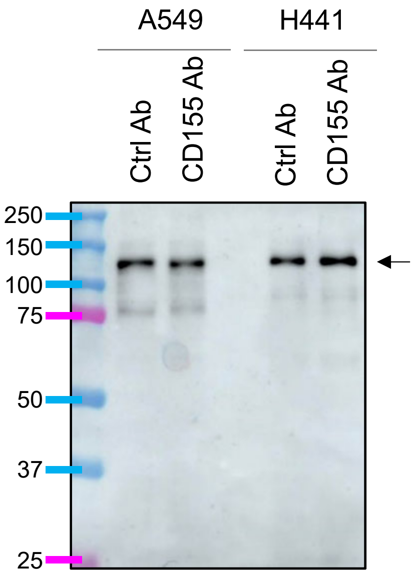

α-FAK
(125kDa)

CST #13009
RRID: AB_2798086 (1:2000)

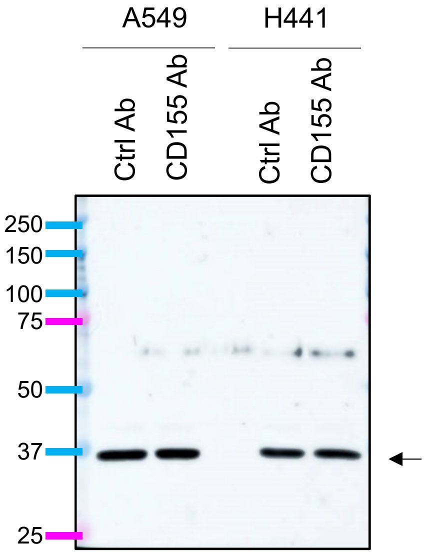

α-GAPDH
(37kDa)
Santacruz #sc25778
RRID: AB_10167668 (1:2000)

Supple Fig. 3C. Uncut images

Supple Fig. 3C

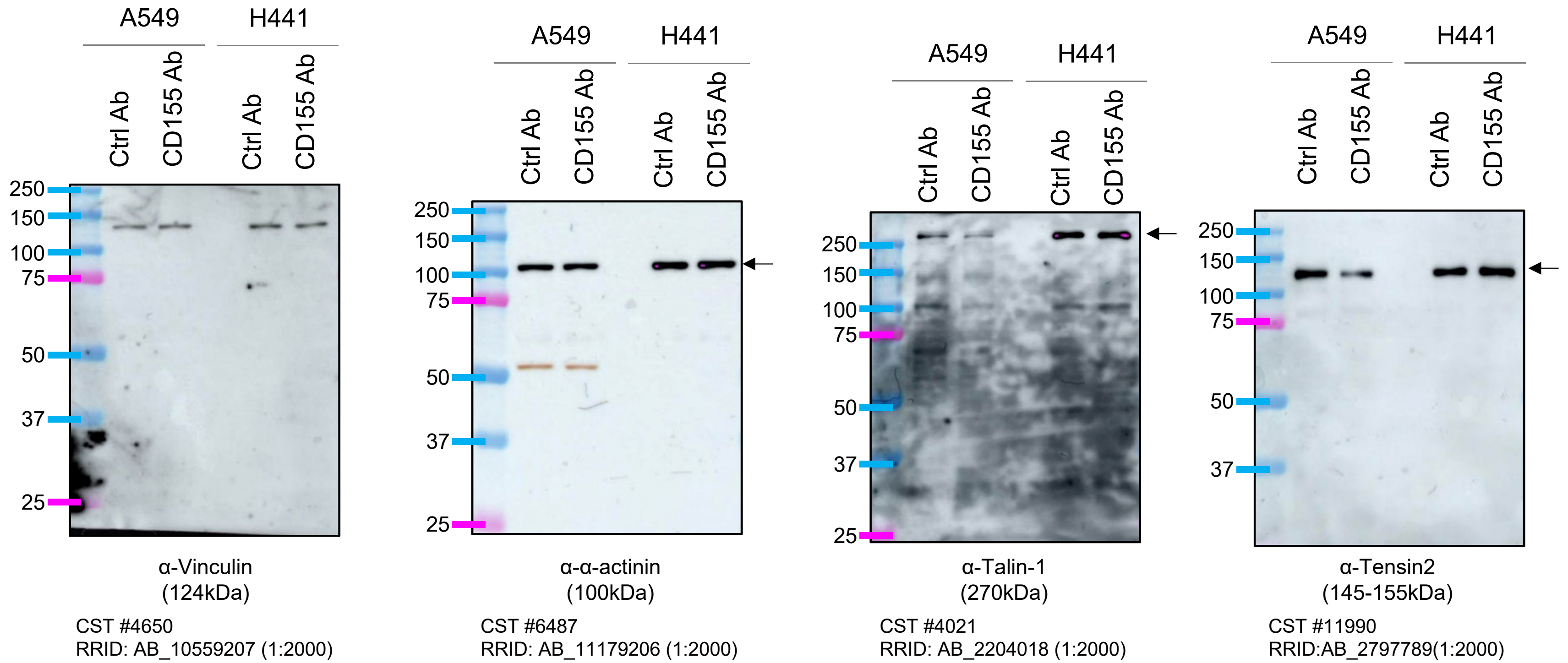

Supple Fig. 3C. Uncut images

Supple Fig. 5a

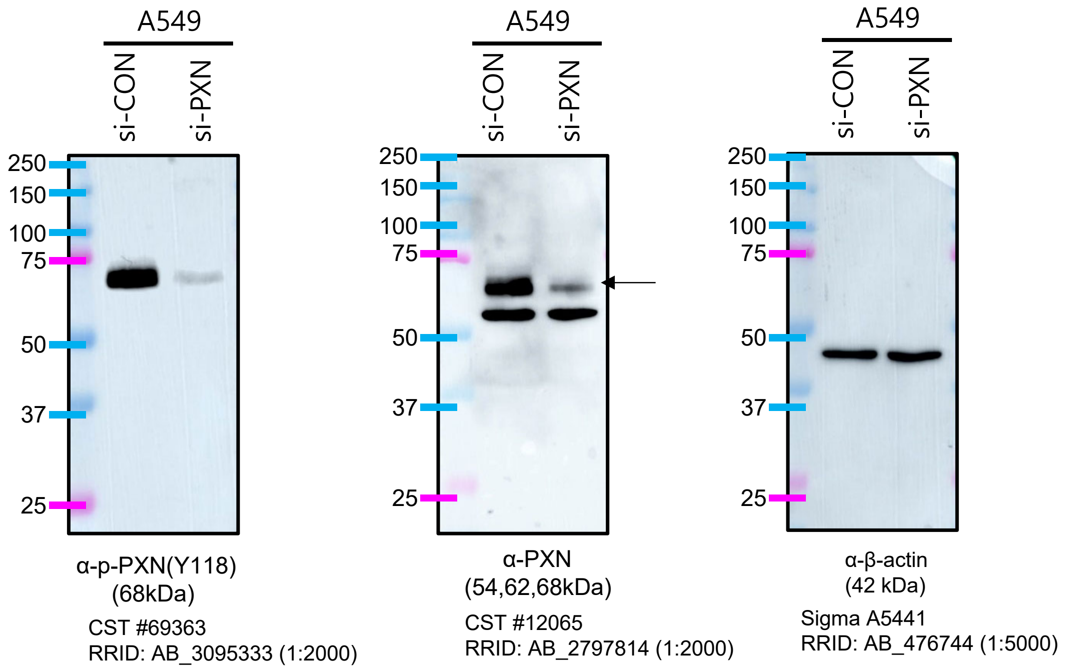

Supple Fig. 5a. Uncut images

Supple Fig. 6a

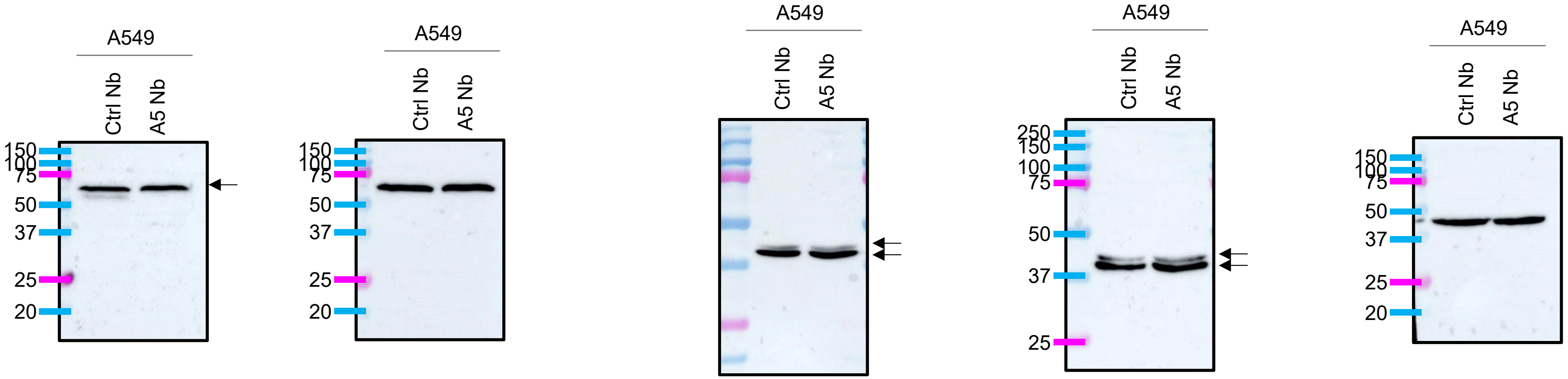

α-p-AKT(Ser473)
(60 kDa)
CST #9271
RRID:AB_329825 (1:2000)

α-AKT
(60 kDa)
CST #9272
RRID:AB_329827 (1:2000)

α-p-ERK1/2(T202/Y204)
(42,44 kDa)
CST #9101
RRID:AB_331646 (1:2000)

α-ERK1/2
(42,44 kDa)
CST #4695
RRID:AB_390779 (1:2000)

α-β-actin
(42 kDa)
Sigma A5441
RRID: AB_476744 (1:5000)

Supple Fig. 6. Uncut images

Supple Fig. 6a

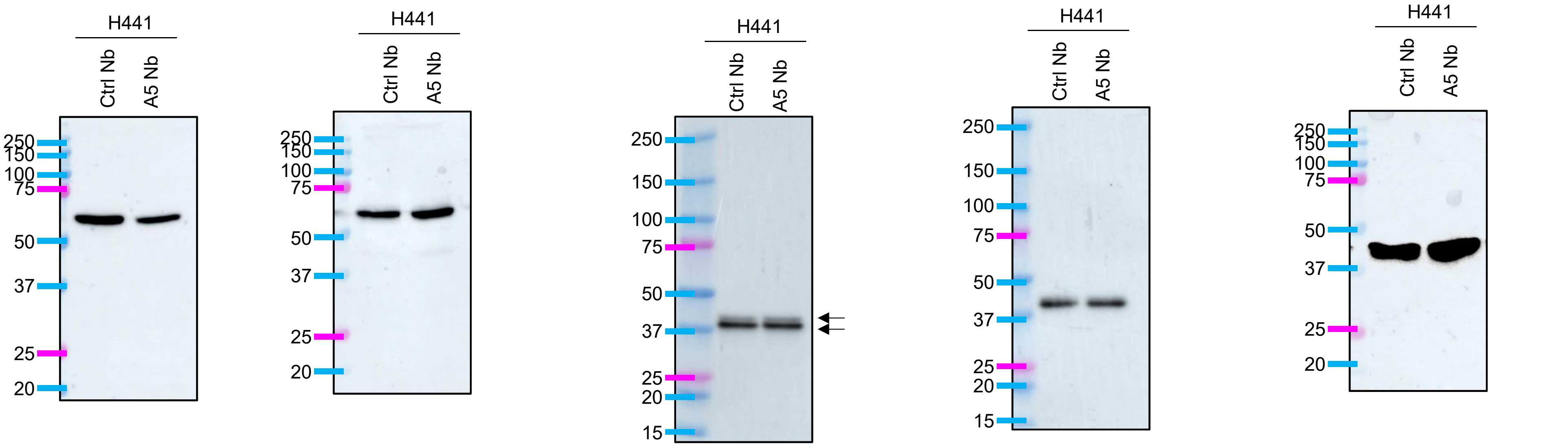

α-p-AKT(Ser473) (60 kDa)
CST #9271
RRID:AB_329825 (1:2000)

α-AKT (60 kDa)
CST #9272
RRID:AB_329827 (1:2000)

α-ERK1/2(T202/Y204) (42,44 kDa)
CST #9101
RRID:AB_331646 (1:2000)

α-ERK1/2 (42,44 kDa
CST #4695
RRID:AB_390779 (1:2000)

α-β-actin (42 kDa)
Sigma A5441
RRID: AB_476744 (1:5000)

Supple Fig. 6. Uncut images

Supple Fig. 6b

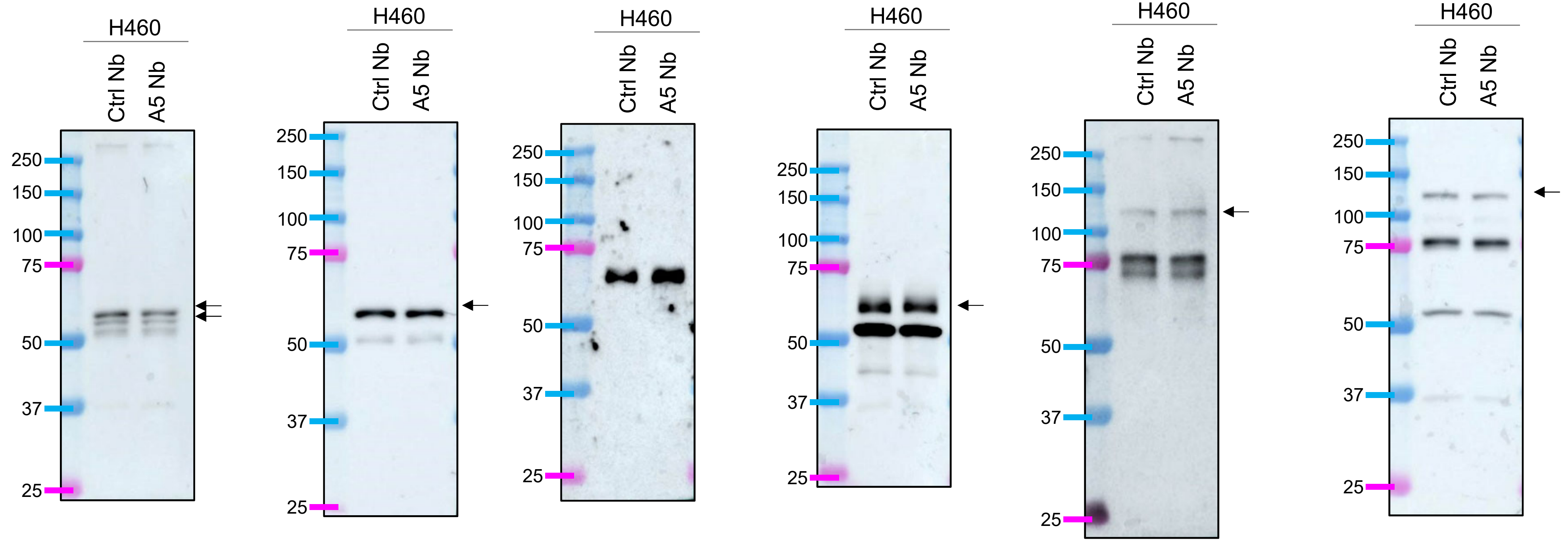

α-p-Src(Y416)
(60kDa)
CST #6943
RRID: AB_10013641 (1:2000)

α-SRC
(60kDa)
CST #2123
RRID: AB_ 2106047(1:2000)

α-p-PXN(Y118)
(68kDa)
CST #69363
RRID: AB_3095333 (1:2000)

α-PXN
(54,62,68kDa)
CST #12065
RRID: AB_2797814 (1:2000)

α-p-FAK(Y397)
(125kDa)
CST #8556
RRID: AB_10891442 (1:2000)

α-FAK
(125kDa)
CST #13009
RRID: AB_2798086 (1:2000)

Supple Fig. 6. Uncut images

Supple Fig. 6b

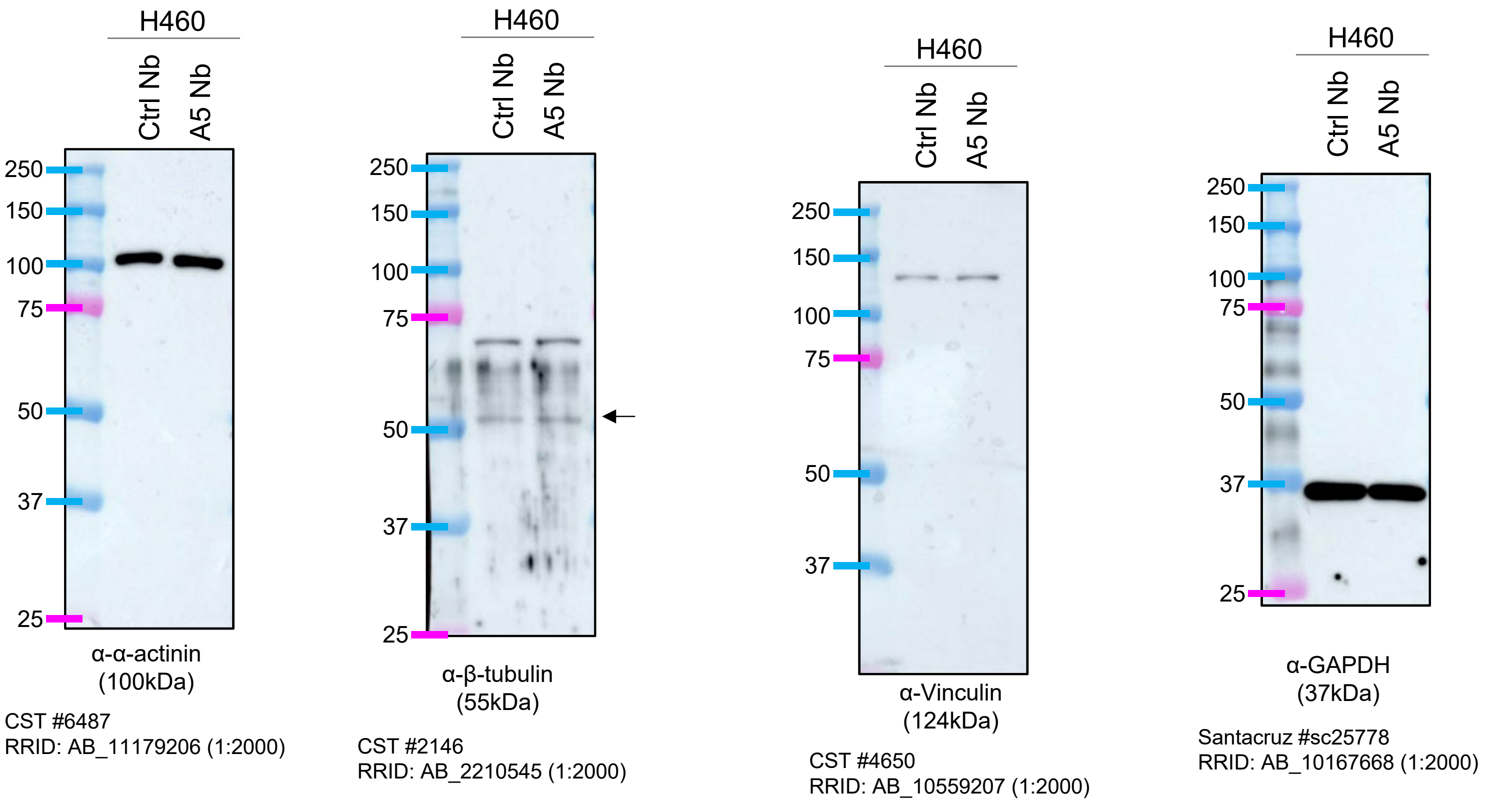

Supple Fig. 6. Uncut images

Supple Fig. 6b

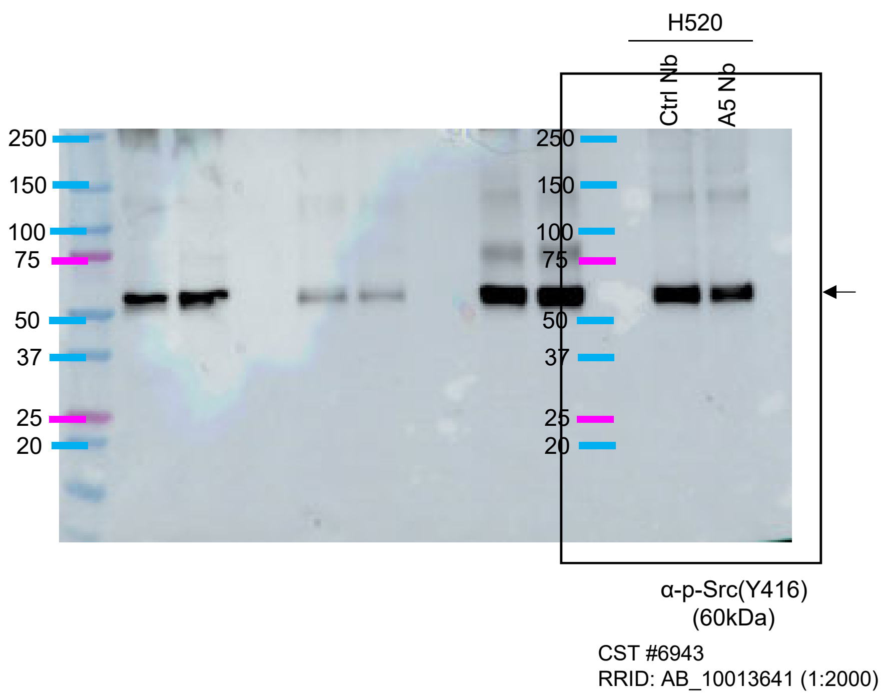

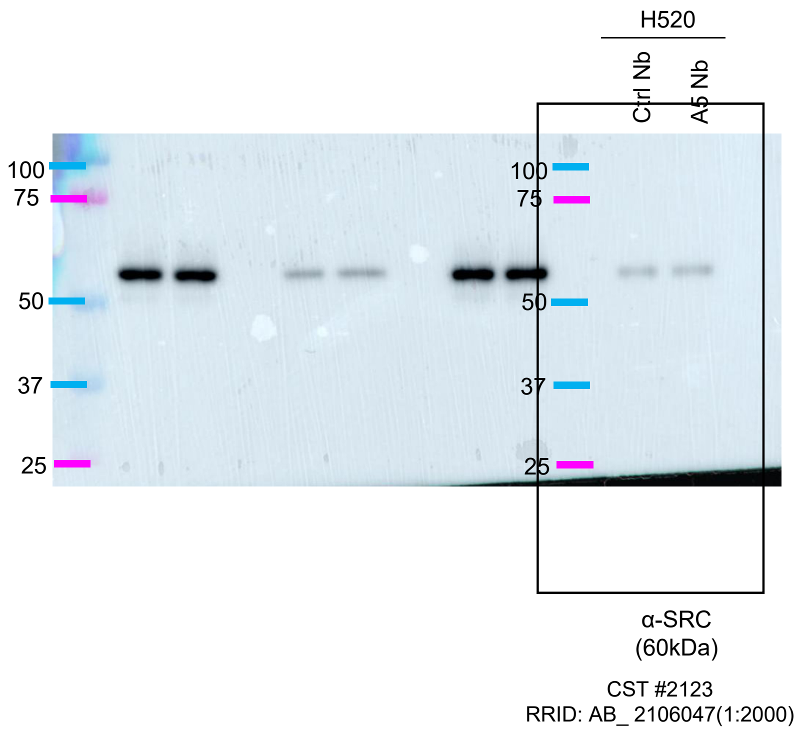

Supple Fig. 6. Uncut images

Supple Fig. 6b

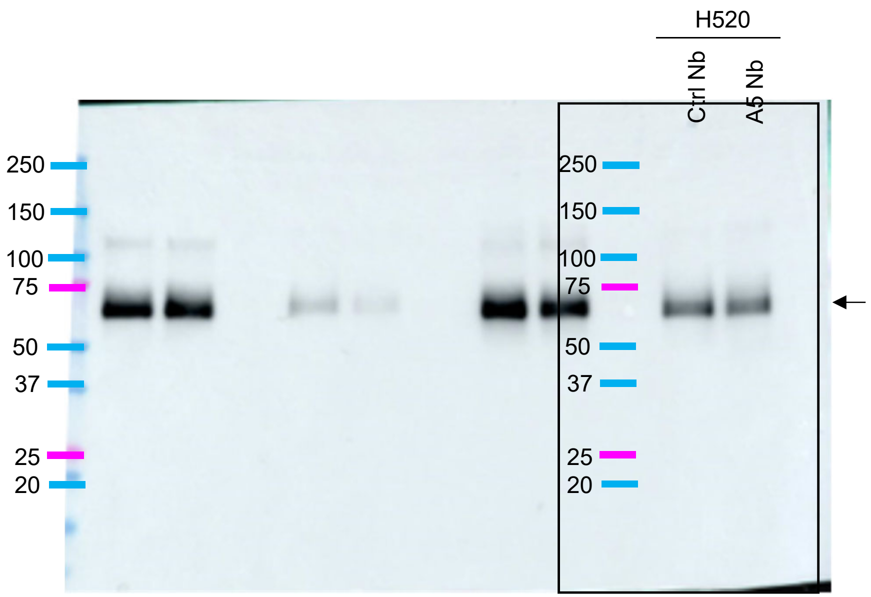

α-p-PXN(Y118)
(68kDa)

CST #69363
RRID: AB_3095333 (1:2000)

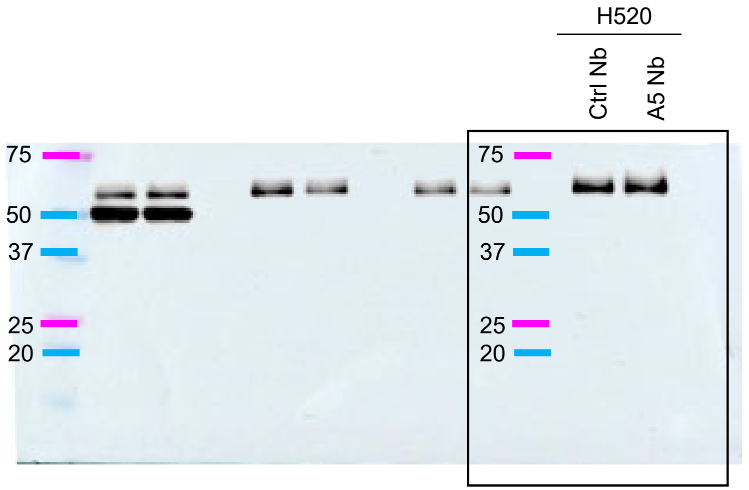

α-PXN
(54,62,68kDa)

CST #12065
RRID: AB_2797814 (1:2000)

Supple Fig. 6. Uncut images

Supple Fig. 6b

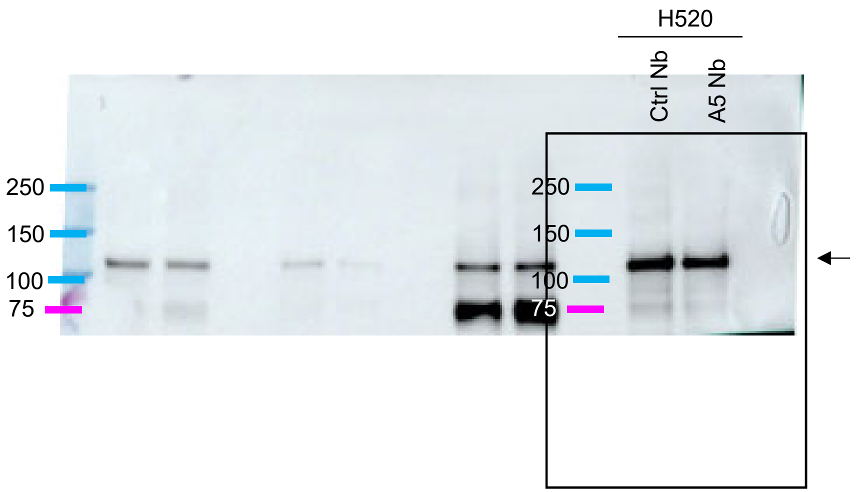

α-p-FAK(Y397)
(125kDa)

CST #8556
RRID: AB_10891442 (1:2000)

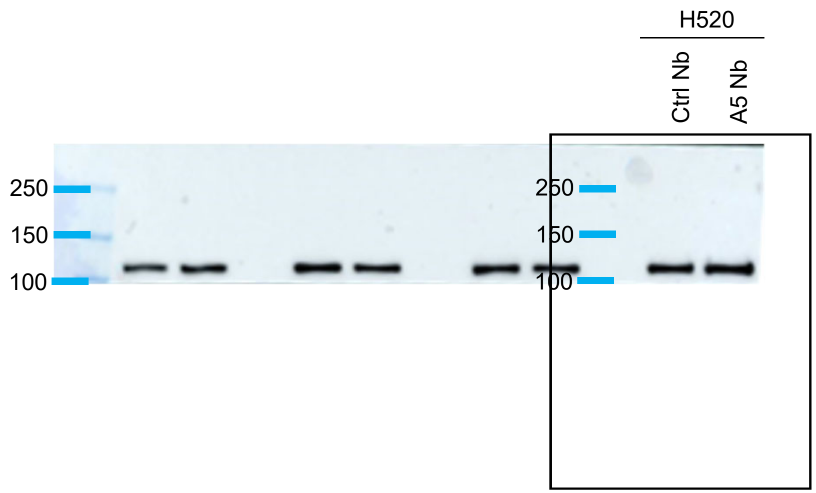

α-FAK
(125kDa)

CST #13009
RRID: AB_2798086 (1:2000)

Supple Fig. 6. Uncut images

Supple Fig. 6b

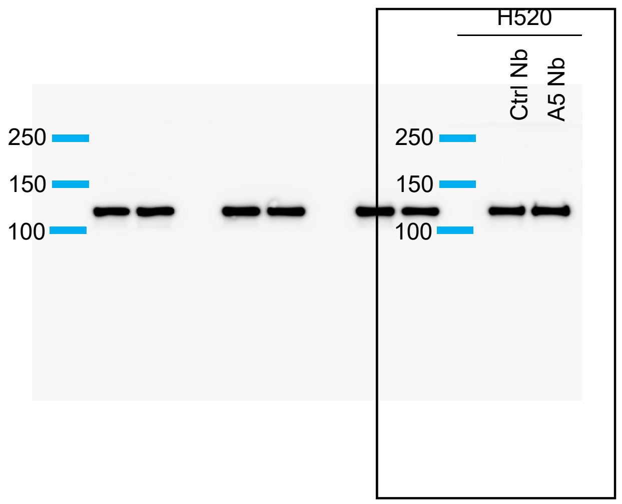

α-Vinculin
(124kDa)

CST #4650
RRID: AB_10559207 (1:2000)

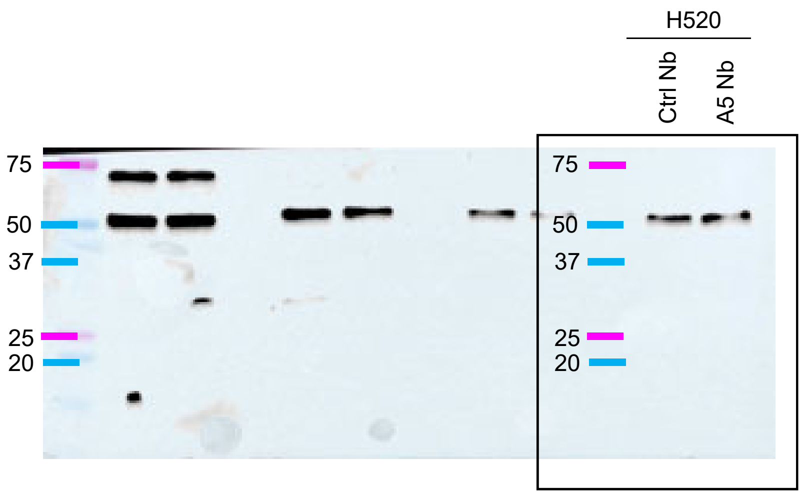

α-β-tubulin
(55kDa)

CST #2146
RRID: AB_2210545 (1:2000)

Supple Fig. 6. Uncut images

Supple Fig. 6b

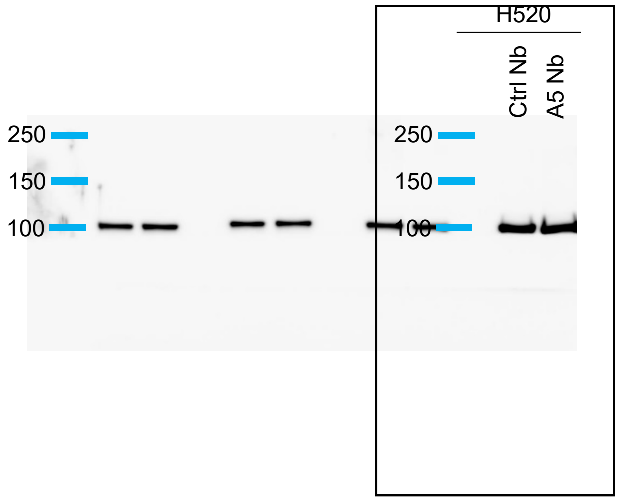

α-α-actinin
(100kDa)

CST #6487
RRID: AB_11179206 (1:2000)

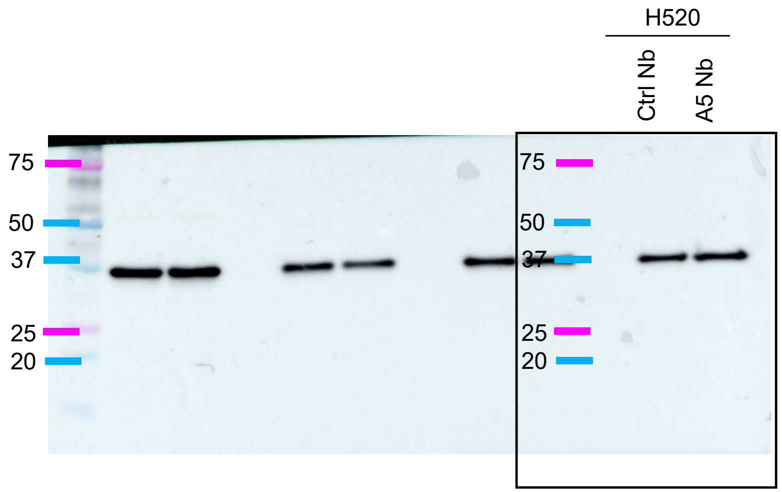

α-GAPDH
(37kDa)

Santacruz #sc25778
RRID: AB_10167668 (1:2000)

Supple Fig. 6. Uncut images

Supple Fig. 6c

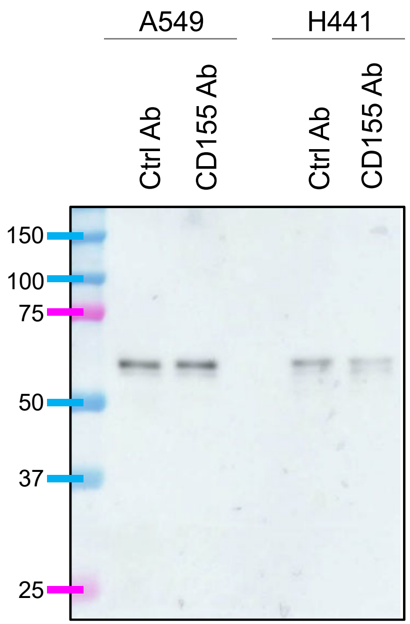

α-p-AKT(Ser473)
(60 kDa)
CST #9271
RRID:AB_329825 (1:2000)

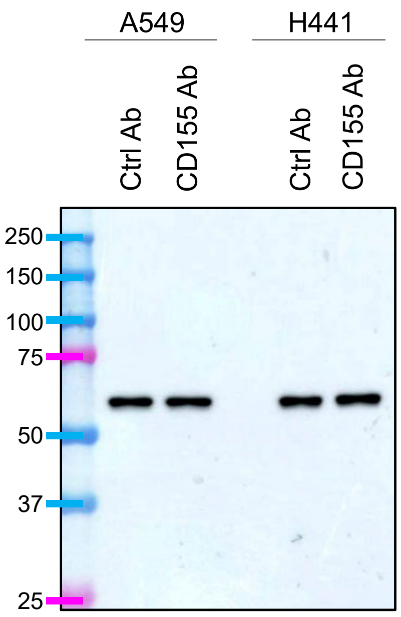

α-AKT
(60 kDa)
CST #9272
RRID:AB_329827 (1:2000)

Supple Fig. 6. Uncut images

Supple Fig. 6c

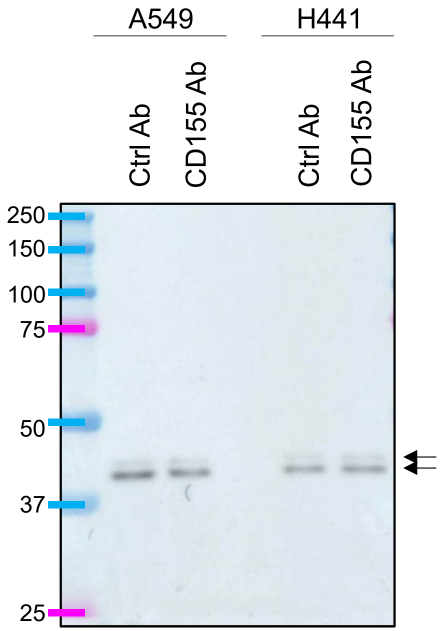

α-p-ERK1/2(T202/Y204)
(42,44 kDa)
CST #9101
RRID:AB_331646 (1:2000)

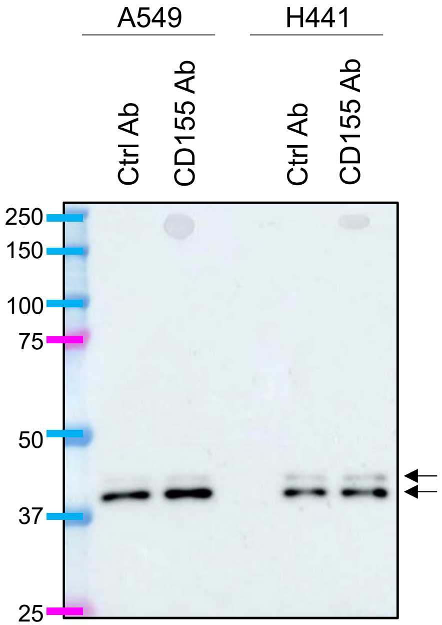

α-ERK1/2
(42,44 kDa)
CST #4695
RRID:AB_390779 (1:2000)

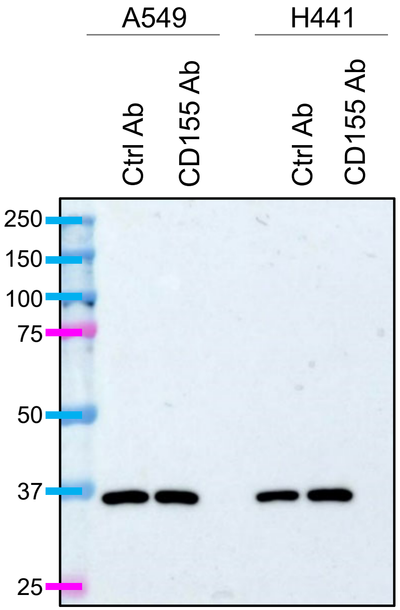

α-GAPDH
(37kDa)
Santacruz #sc25778
RRID: AB_10167668 (1:2000)

Supple Fig. 6. Uncut images

Supple Fig. 6d

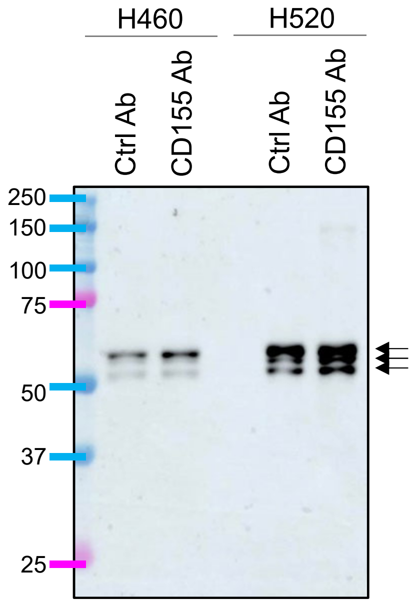

α-p-Src(Y416)
(60kDa)

CST #6943
RRID: AB_10013641 (1:2000)

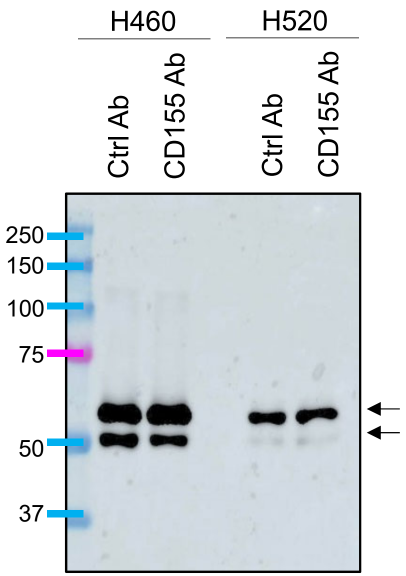

α-SRC
(60kDa)

CST #2123
RRID: AB_ 2106047(1:2000)

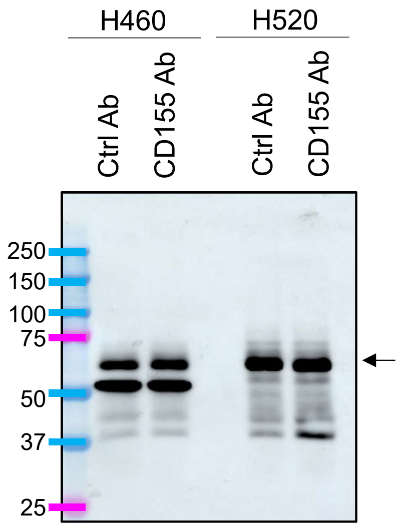

α-p-PXN(Y118)
(68kDa)

CST #69363
RRID: AB_3095333 (1:2000)

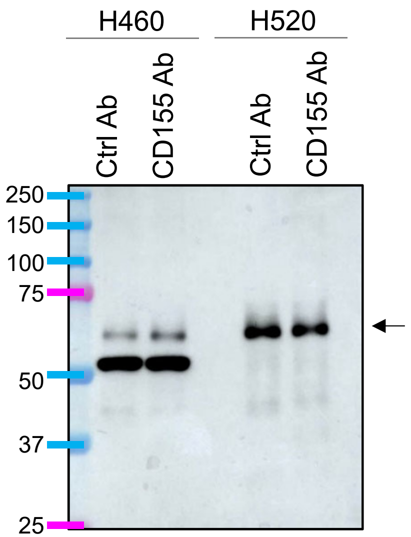

α-PXN
(54,62,68kDa)

CST #12065
RRID: AB_2797814 (1:2000)

Supple Fig. 6. Uncut images

Supple Fig. 6d

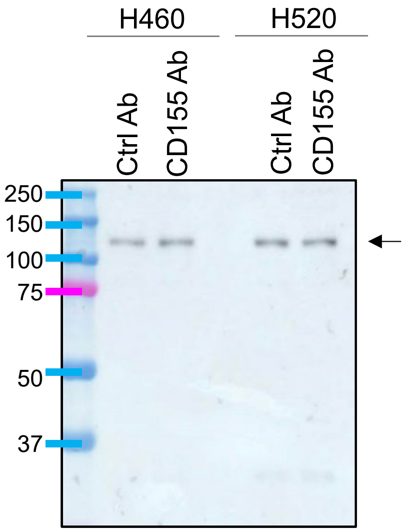

α-p-FAK(Y397)
(125kDa)

CST #8556
RRID: AB_10891442 (1:2000)

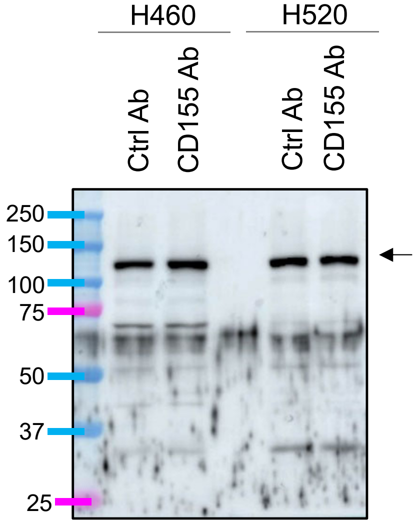

α-FAK
(125kDa)

CST #13009
RRID: AB_2798086 (1:2000)

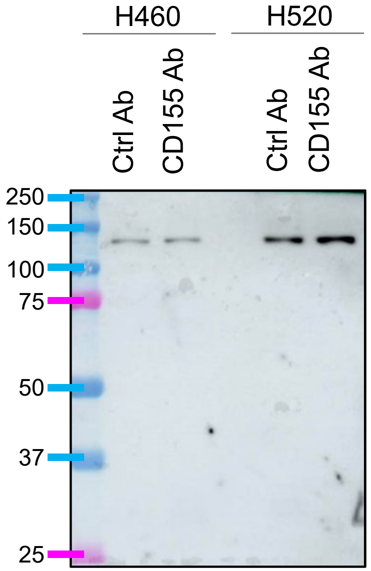

α-Vinculin
(124kDa)

CST #4650
RRID: AB_10559207 (1:2000)

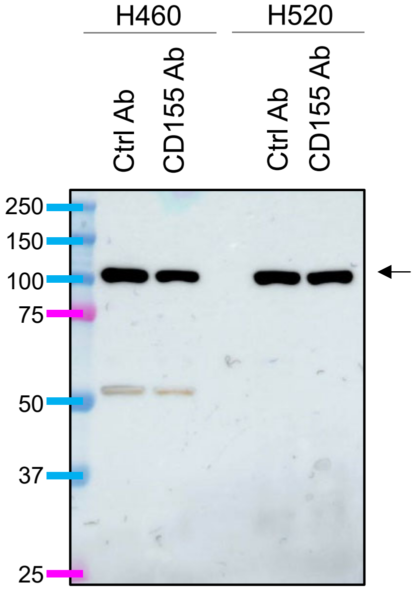

α-α-actinin
(100kDa)

CST #6487
RRID: AB_11179206 (1:2000)

Supple Fig. 6. Uncut images

Supple Fig. 6d

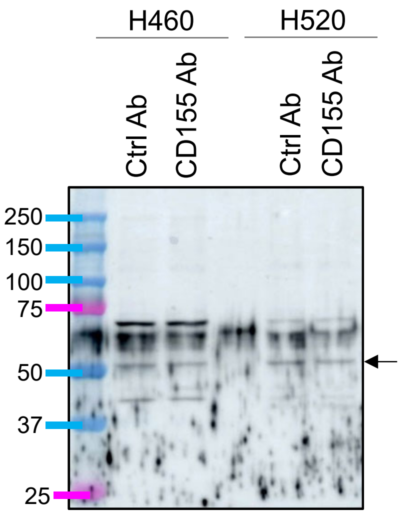

α-β-tubulin
(55kDa)

CST #2146
RRID: AB_2210545 (1:2000)

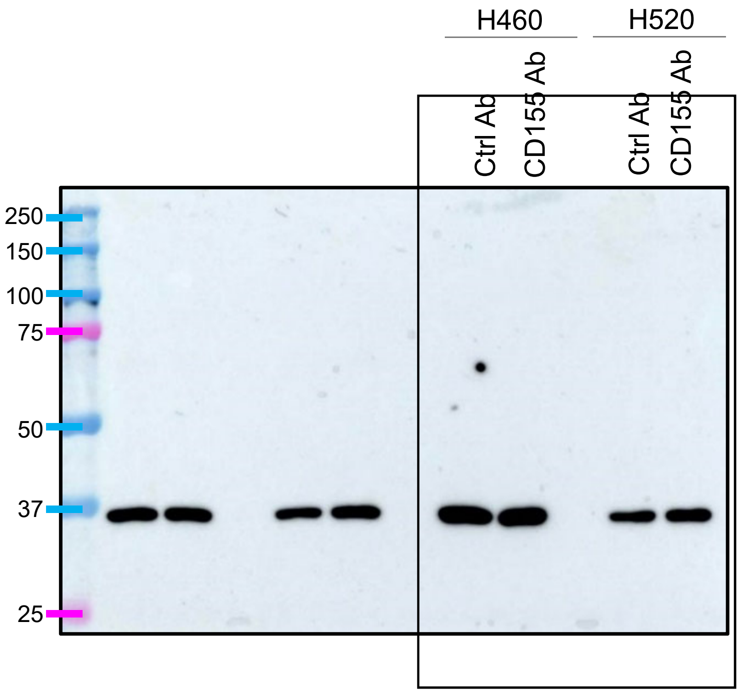

α-GAPDH
(37kDa)

Santacruz #sc25778
RRID: AB_10167668 (1:2000)

Supple Fig. 6. Uncut images

Supple Fig. 7e

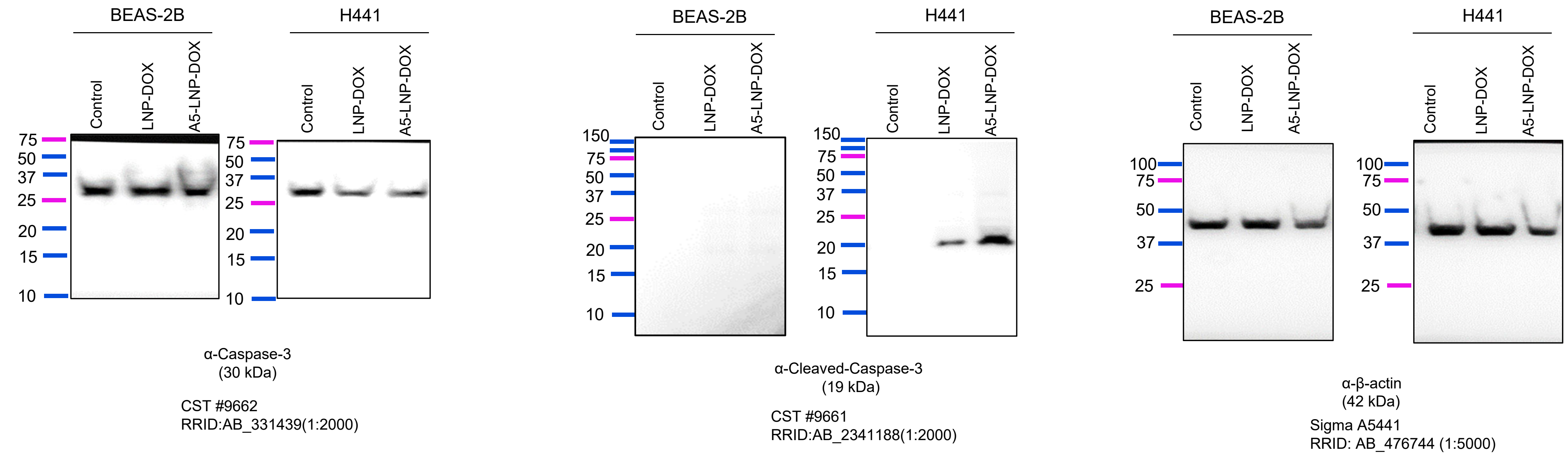

Supple Fig. 7. Uncut images

Supple Fig. 9d

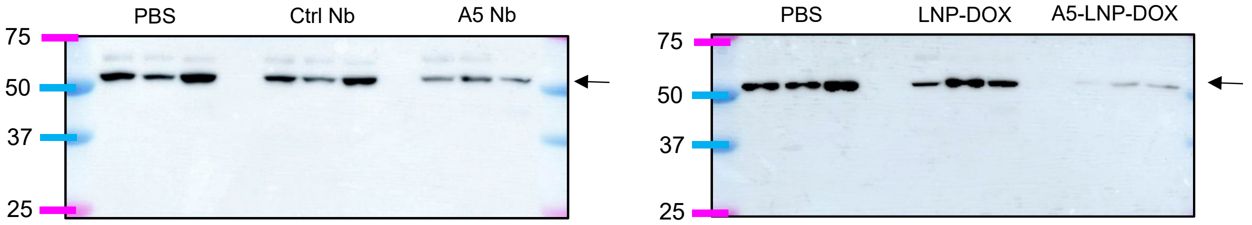

α-PXN
(54,62,68kDa)

CST #12065
RRID: AB_2797814 (1:2000)

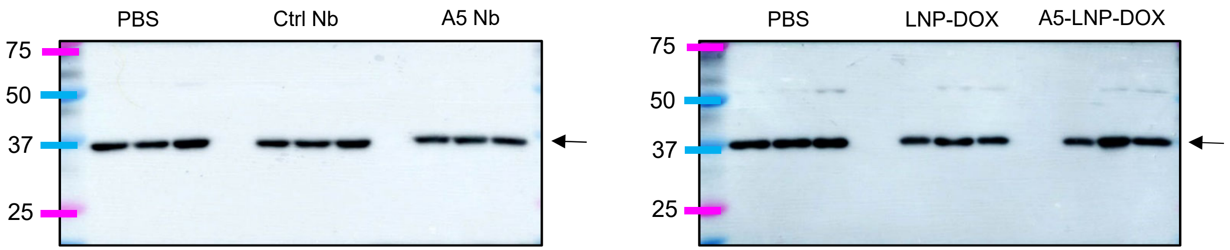

α-GAPDH
(37kDa)

Santacruz #sc25778
RRID: AB_10167668 (1:2000)

Supple Fig. 9. Uncut images
